# Influence of the Glass Transition Temperature and the Density of Crosslinking Groups on the Reversibility of Diels-Alder Polymer Networks

**DOI:** 10.3390/polym13081189

**Published:** 2021-04-07

**Authors:** Merlina Thiessen, Volker Abetz

**Affiliations:** 1 Institute of Physical Chemistry, Universität Hamburg, Grindelallee 117, 20146 Hamburg, Germany; merlina.thiessen@chemie.uni-hamburg.de; 2 Institute of Membrane Research, Helmholtz-Zentrum Hereon, Max-Planck-Straße 1, 21502 Geesthacht, Germany

**Keywords:** Diels-Alder, polymer network, thermoreversibility

## Abstract

The interest in self-healing, recyclable, and adaptable polymers is growing. This work addresses the reversibility of crosslink formation based on Diels-Alder reaction in copolymer networks containing furfuryl and maleimide groups, which represent the “diene” and the “dienophile,” respectively. The copolymers are synthesized by atom transfer radical polymerization (ATRP) and free radical polymerization. The diene bearing copolymers are crosslinked either with a small molecule containing two dienophiles or with a dienophile bearing copolymer. The influence of the crosslinking temperature on the Diels-Alder reaction is analyzed. Furthermore, the influence of the glass transition temperature and the influence of the density of crosslinking groups on the thermo-reversibility of crosslinking are investigated by temperature dependent infrared spectroscopy and differential scanning calorimetry. It is shown that the reversibility of crosslinking is strongly influenced by the glass transition temperature of the system.

## 1. Introduction

**Figure 1:**
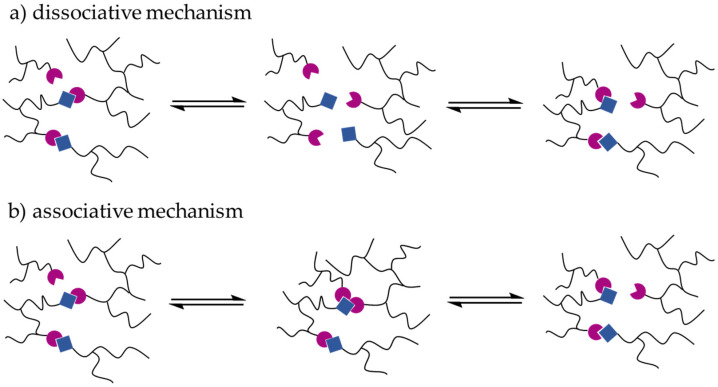
Difference between a covalent adaptable network (CAN) with a dissociative (**a**) and an associative mechanism (**b**). In dissociative CANs, the network loses its integrity while, in associative CANs, an exchange reaction takes place so that the polymer network is kept. The blue-colored and magenta-colored shapes serve to represent the crosslinking points of the CAN.

Polymer materials with adaptable properties are of increasing interest. An important aspect is their sustainable use if they are showing self-healing properties and are recyclable without degradation and loss of properties. Stimuli-responsive polymers are able to change properties by various physical, chemical, or biochemical stimuli. Examples for stimuli are temperature [1], pH [2], mechanical load [3], light [4], or the redox potential [5]. In this work, the goal is the development of thermoreversible polymer networks that the melt allows at high temperatures and, therefore, become processable and, after cooling, form again a hard and insoluble polymer network. In order to synthesize polymers with such properties, there are basically two different ways:

(i) Polymers can be either crosslinked via non-covalent interactions following the concepts of “supramolecular chemistry” [6,7]. Supramolecular polymer networks building blocks are held together by relatively weak intermolecular or intramolecular interactions, such as hydrogen bonds [8,9,10,11,12], π-π interactions [13,14], or metal-ligand interactions [15,16,17]. An advantage of these types of interactions is that they open and close fast upon increasing and lowering temperature. In principle, thermoplastics and thermoplastic elastomers based on semicrystalline polymers or hard-soft segmented and microphase separated block copolymers with at least two hard blocks can be considered as thermo-reversible networks, where processability occurs above the melting point or the order-disorder transition. However, these systems typically still have a high melt viscosity because the molecular weights are high. However, low viscosity of the polymer melt can be achieved if low molecular weight, unentangled polymers can be used, which facilitates the production and processing of products.
(ii) Polymers can also be covalently, reversibly crosslinked leading to “covalent adaptable networks” (CANs) [18]. Dissociative and associative CANs can be distinguished. This subdivision is based on the type of exchange reaction of the crosslinks. If the network is broken during the reaction, it is called a dissociative CAN (Figure 1a), like in the case of the Diels-Alder reaction. If the network is only changed during an exchange reaction but remains permanently crosslinked, it is called an associative CAN (Figure 1b) [19]. Vitrimers are typical examples for associative CANs [19].

**Figure 2:**
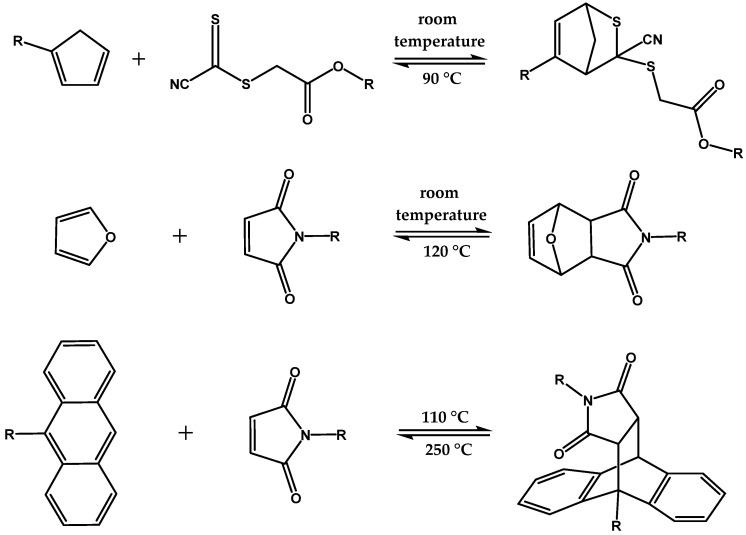
Various Diels-Alder motifs that can be incorporated into a polymer, with their respective products and the corresponding temperatures for the Diels-Alder and retro-Diels-Alder reaction.

The Diels-Alder reaction of a diene with a dienophile is one of the best known thermo-reversible chemical reactions. At low temperatures, a [4+2] cycloaddition occurs and, at elevated temperatures, a cyclo-reversion occurs, which is called a retro-Diels-Alder reaction [20]. In addition to the application of the Diels-Alder reaction in natural product synthesis, the use of Diels-Alder motifs in polymer chemistry is becoming increasingly important. The Diels-Alder reaction is useful for the synthesis of thermo-reversible polymer networks, as a wide range of temperatures can be utilized using various diene and dienophile bearing groups (Figure 2) [18,21,22,23,24].

**Figure 3:**
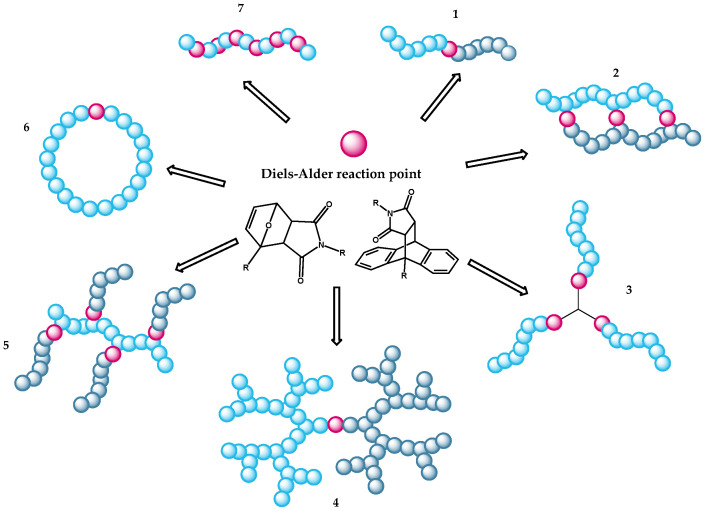
Possible macromolecular architectures that can be formed via a Diels-Alder reaction at special topological locations. The magenta spheres serve as a representation of the points where bonds are created by a Diels-Alder reaction. The bright and dark blue spheres are supposed to represent polymers. In addition to linear polymers (7), block copolymers (1), rings (6) or polymer networks (2), as described in this work, can be created.

Functionalization of different polymers with different dienes and dienophiles results in materials with a wide temperature range of the opening and closing of the polymer network [20,25]. In addition, polymers with other topologies can be developed (Figure 3). Polymers such as polystyrene [26,27], polyketones [28,29], polymethacrylates [30,31], or natural rubber [32,33,34,35] can be functionalized with Diels-Alder motifs. One advantage of thermoreversible crosslinking is the simplified processing of the polymer at high temperatures. Furthermore, such systems can show self-healing properties due to the reversible bond cleavage described above.

The reversibility of the Diels-Alder reaction of furan and maleimide has been discussed before [24,25,26,27,28,29,30,31,32,33,34,35]. It was shown that a de-crosslinking of the system takes place above a temperature of 120 °C [36]. The thermoreversibility of crosslinks in a furfuryl bearing polymer crosslinked with a bismaleimide was confirmed by differential scanning calorimetry (DSC), rheological measurements, or by temperature-dependent Fourier transform infrared spectroscopy (FTIR). Furthermore, it is known that the flexibilities of the polymer [37,38] and the bismaleimide linker [39] have an influence on the self-healing properties of this type of Diels-Alder systems. While Zeng et al. discussed the influence of the mobility of the bismaleimide crosslinker on a bio-based poly(2,5-furandimethylene succinate) [39], Engel, Schäfer, and Kickelbick described the influence of the glass transition temperature on the healing capacity of Diels-Alder polymer nanocomposites with surface functionalized silica nanoparticles [37,38]. In comparison to these former studies, this work deals with the crosslinking of two different polymers via the Diels-Alder reaction and examines their thermo-reversible properties.

The general motivation of the work is the development of polymer networks with thermo-reversible crosslinking points based on low molecular weight polymers in order to achieve easy processible materials at a higher temperature with a self-healing capability. The Diels-Alder reaction offers a reversible covalent linkage, which can be controlled by temperature. In this way, strong polymer networks can be formed even at room temperature, which become a liquid by an increase in temperature. In order to better understand the effects of the polymer on reversibility of the crosslinks, different factors were investigated. In addition to the influence of the crosslinking temperature and the density of crosslinking groups, the influence of the glass transition temperature on the reversibility of the chosen crosslinked systems was investigated.

## 2. Materials and Methods

### 2.1. Materials

The furan-protected maleimide monomer was synthesized in a three-step reaction and the following chemicals were used. For the first step, maleic anhydride (99%, Sigma-Aldrich, Schnelldorf, Germany) and furan (≥99% with 0.025 wt% 3,5-di-*tert*-butyl-4-hydroxytoluene as inhibitor, Sigma-Aldrich) were dissolved in diethyl ether (99.5%, Grüssing, Filsum, Germany). For the next step, the product from step 1 was dissolved in methanol (99.9%, Acros, Fairlawn, NJ, USA) and ethanolamine (99.5%, Sigma-Aldrich, Schnelldorf, Germany) was added. The product was washed in isopropyl alcohol (99.5%, VWR chemicals, Darmstadt, Germany). In the third step, the product from step 2 and triethylamine (99%, Grüssing) was dissolved in dichloromethane (99.9% with amylene as stabilizer, Acros) and methacryloyl chloride (97% with 200 ppm monoethyl ether hydroquinone as stabilizer) was added. For purification, the product was washed with a concentrated aqueous sodium bicarbonate (99%, Grüssing) solution, demineralized water, and dried over magnesium sulfate (99%, Grüssing). Afterward, the crude oil was filtered by a silica column with ethyl acetate and dichloromethane as eluents. For stabilization of the product, 3,5-di-*tert*-butyl-4- hydroxytoluene (BHT, 99.8%, Acros) was added.

Prior to synthesis, the monomers were filtered via basic alumina (≥98%, Honeywell Fluka, Seelze, Germany) to remove the inhibitors. Homopolymers and copolymers with a diene function were synthesized by atom transfer radical polymerization (ATRP) from furfuryl methacrylate (FMA, 97% with 200 ppm monoethyl ether hydroquinone as inhibitor, Sigma-Aldrich), methyl methacrylate (MMA, 99% with ≤30 ppm monoethyl ether hydroquinone as inhibitor, Merck, Darmstadt, Germany), and *n*-butyl acrylate (BA, ≥99% with 10–60 ppm monoethyl ether hydroquinone as inhibitor, Merck) in anisole (≥99%, Merck). Copper (I) bromide (≥98%, Fluka, München, Germany), *N*,*N*,*N*′,*N*″,*N*″-Pentamethyldiethylene-triamine (PMDETA, 98%, TCI Chemicals, Eschborn, Germany) and ethyl α-bromoisobutyrate (EBiB, 98%, Sigma-Aldrich) were used as the initiator system. The diene bearing homopolymer was precipitated in *n*-hexane (98%, VWR Chemicals) and the copolymers were precipitated in methanol (technical grade). The copolymer with a dienophile function was synthesized from maleimide methacrylate (MIMA, own synthesis) and methyl methacrylate (99% with ≤30 ppm monoethyl ether hydroquinone as inhibitor, Merck) in tetrahydrofuran (THF, >99%, with 3,5-di-*tert*-butyl-4-hydroxytoluene as stabilizer, VWR chemicals) with 2,2′-Azobis(2-methylpropionitrile) (98%, Sigma-Aldrich). The copolymer was precipitated in methanol (technical grade).

For crosslinking of the diene bearing copolymers with a low molecular weight dienophile, 1,1′-(methylendi-4,1-phenylene) bismaleimide (BMI, >96%, TCI Chemicals) was used. As solvents, dichloromethane (DCM, 99.9% with amylene as stabilizer, Acros) and anisole (≥99%, Merck), were used.

### 2.2. Synthesis of Furan Protected Maleimide Methacrylate (MIMA)

**Figure 4:**
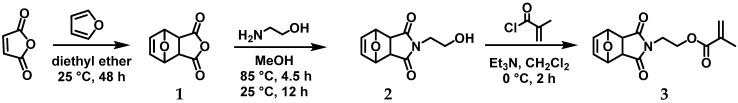
Reaction scheme of the furan protected maleimide methacrylate.

The synthesis of maleimide methacrylate (Figure 4) was modified from literature [40,41]. Maleic anhydride (1 Eq.) was dissolved in diethyl ether and furan (5 Eq.) was added under stirring. The reaction mixture was stirred for 48 h at room temperature. The white crystallites 1 were filtered and washed with diethyl ether before drying. Product 1 (1 Eq.) was then dissolved in methanol and cooled with an ice bath. Ethanolamine (1.04 Eq.) was added dropwise to the reaction mixture. Afterwards, the reaction mixture was stirred for 4.5 h at 85 °C and 12 h at room temperature. The white crystallites 2 were filtered and washed with cold isopropyl alcohol before drying. The alcohol 2 (1 Eq.) and triethylamine (1.2 Eq.) were dissolved in dry dichloromethane and cooled at 0 °C. Afterward, methacryloyl chloride (1.05 Eq.) was added dropwise and the reaction mixture was stirred for 2 h at 0 °C. The reaction mixture was diluted and extracted with dichloromethane and washed three times with sodium bicarbonate (aq.) and demineralized water. The combined organic layers were dried over magnesium sulfate. After removal of the solvent, the crude oil 3 was cleaned through a short silica column (ethyl acetate: dichloromethane = 4:5). Before removing the solvent, 3,5-di-*tert*-butyl-4-hydroxytoluene was added as an inhibitor.

### 2.3. Synthesis of Furfuryl Bearing Polymers by ATRP

The polymerization was performed under exclusion of oxygen. Prior to polymerization, the monomers were filtered via basic alumina to remove the inhibitors.

**Figure 5:**
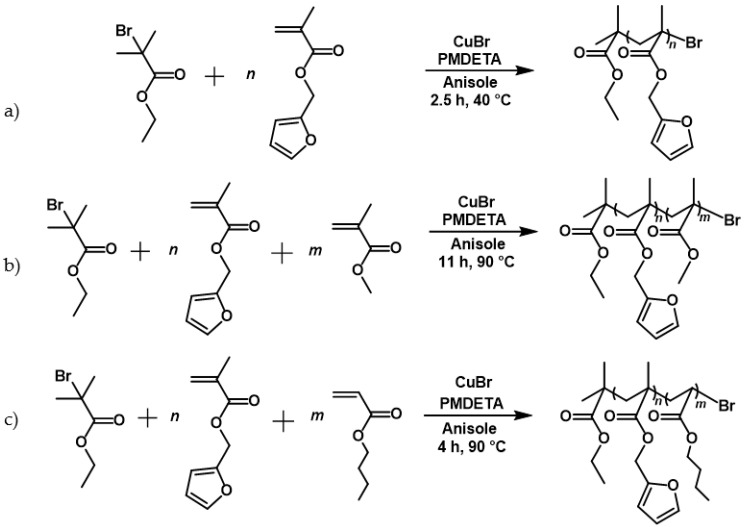
Reaction scheme of: (**a**) the homo-polymerization of furfuryl methacrylate. Atom transfer radical polymerization (ATRP) conditions: monomer:initiator:catalyst:ligand is 50:1:0.3:0.3 in 70 wt% anisole. (**b**) The copolymerization of methyl methacrylate and furfuryl methacrylate. ATRP conditions: monomer:initiator:catalyst:ligand is 100:1:1:1 in 50 wt% anisole and (**c**) the copolymerization of butyl acrylate and furfuryl methacrylate. ATRP conditions: monomer:initiator:catalyst:ligand is 100:1:1:1 in 50 wt% anisole.

The monomers (FMA, MMA, and BA), the solvent (anisole), and PMDETA were placed in a three-necked flask and degassed once. Copper(I)bromide was then added, and the solution was degassed twice. After the initiator (EBiB) was added, the reaction solution was stirred with a preheated oil bath for the appropriate reaction time under nitrogen. The polymerization was quenched with air and cooling in an ice bath. The solution was diluted with tetrahydrofuran and copper(I)bromide was removed via a neutral alumina column. The polymer solution was concentrated by rotary evaporation, and then it was precipitated three times in cold methanol (copolymers with BA and MMA) or *n*-hexane (FMA homopolymer) and dried under reduced pressure. The dried polymers were dissolved in tetrahydrofuran before each precipitation. The conditions and the reaction scheme of ATRP are shown in Figure 5.

### 2.4. Synthesis of Maleimide Bearing Copolymer by Free Radical Polymerization

As before, the reaction was performed under exclusion of oxygen. Prior to polymerization, the monomers were filtered via basic alumina to remove the inhibitors.

The free radical polymerization of the furan protected methyl methacrylate-*co*-maleimide methacrylate copolymer was done according to literature [42]. First, a solution of methyl methacrylate (2 Eq.) and the furan protected maleimide methacrylate (1 Eq.) in dry tetrahydrofuran (weight ratio: monomer:solvent 1:20) was prepared. After adding 2,2′-azobisisobutyronitrile (AIBN, 0.1 Eq.) to the solution, it was degassed two times. The reaction solution was stirred for 7.5 h at 65 °C under nitrogen. The reaction was stopped by quenching with air and cooling the reaction solution with an ice bath. The polymer solution was concentrated by rotary evaporation, and then it was precipitated three times in cold methanol and dried under reduced pressure. The dried polymer was dissolved in tetrahydrofuran before each precipitation.

**Figure 6:**
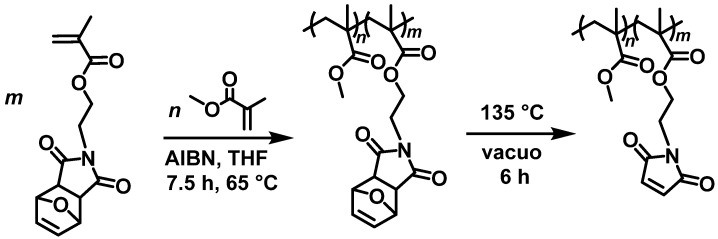
Reaction scheme of the copolymerization of methyl methacrylate and furan-protected maleimide methacrylate and deprotection of the maleimide side groups.

For deprotection of the maleimide ring, the polymer was heated at 135 °C for 6 h under vacuum. The reaction scheme is shown in Figure 6.

### 2.5. Crosslinking of Furfuryl Bearing Polymers

**Figure 7:**
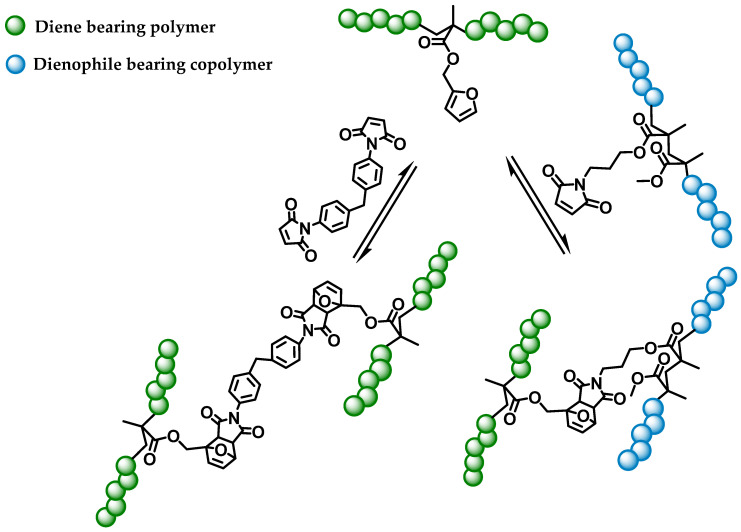
Crosslinking paths via the Diels-Alder reaction of furfuryl and maleimide bearing groups.

Figure 7 shows the two ways of crosslinking of the furfuryl (diene) containing homo- and copolymers. This happens by a small crosslinker molecule, a bismaleimide, or by a dienophile-containing copolymer.

#### 2.5.1. Crosslinking at Room Temperature

For crosslinking at room temperature, the furfuryl-bearing polymer, and the crosslinker BMI or the furfuryl-bearing and maleimide-bearing polymers are dissolved in dichloromethane (5 wt%) while stirring. The crosslinker was used in different motif ratios (furfuryl-groups in polymer: maleimide groups in BMI; 1:0.5; 1:1; 1:2), and the polymers in a ratio of 1:1 (furfuryl-groups in polymer: maleimide-groups in polymer). The reaction solutions were stirred for at least 3 days.

#### 2.5.2. Crosslinking at 120 °C

For crosslinking at 120 °C, the furfuryl-bearing polymer, and the crosslinker BMI or the furfuryl-bearing and maleimide-bearing polymers are dissolved in dichloromethane (3 wt%) while stirring. The crosslinker was used in different motif ratios (furfuryl-groups in polymer: maleimide groups in BMI, 1:1, 1:2). The polymers are in a ratio of 1:1 (furfuryl-groups in polymer: maleimide-groups in polymer). The reaction solutions were stirred for 2 h at room temperature and then 2 h at 120 °C (oil bath temperature) under reflux. Afterward, the reaction solutions were stirred for another 12 h at room temperature.

#### 2.5.3. Crosslinking at 120 °C and 165 °C

For crosslinking at 120 °C and 165 °C, the furfuryl-bearing polymer, and the crosslinker BMI or the furfuryl-bearing and maleimide-bearing polymers are dissolved in anisole (13 wt%) while stirring. The crosslinker was used in the motif ratio 1:2 (furfuryl-groups in polymer: maleimide groups in BMI) and the polymers were used in a ratio of 1:1 (furfuryl-groups in polymer: maleimide-groups in polymer). The reaction solutions were stirred for 12 h at 120 °C (oil bath temperature) and then 6 h at 165 °C (oil bath temperature) under reflux.

### 2.6. Characterization

#### 2.6.1. Size Exclusion Chromatography

The apparent number average molecular weight *M*_n_ of the homopolymers and copolymers as well as the dispersity index (*Đ*) were determined by size exclusion chromatography (SEC, calibrated to PMMA). All SEC measurements were performed at 30 °C in THF on a PSS Agilent 1260 Infinity system equipped with a PSS Security auto injector, and a PSS Security isocratic pump with a flow rate of 1 mL/min (PSS Polymer Standards Service GmbH, Mainz, Germany). One pre-column and three analytical columns with porosities of 103, 105, and 106 Å-consisting of modified styrene-divinylbenzene-copolymer gels as stationary phase (PSS Polymer Standards Service GmbH, Mainz, Germany)-were used. As an internal standard, toluene was added. Detection of toluene was performed with a UV-Vis wavelength detector at 260 nm (PSS Security, light source: deuterium lamp, wavelength range of 190−600 nm). For polymer samples with concentrations of 1 mg/mL and an injection volume of 100 µL, a refractive index detector (PSS Security differential-refractometer-detector) was used. Data processing was done with WinGPC UniChrom (PSS Polymer Standards Service GmbH, Mainz, Germany).

#### 2.6.2. Nuclear Magnetic Resonance Spectroscopy

Proton nuclear magnetic resonance (^1^H NMR) spectra of all monomers and polymers were recorded on a Bruker AVANCE II (Bruker BioSpin GmbH, Karlsruhe, Germany) at 400 MHz. Tetramethylsilane (TMS) was used as an internal standard, and chloroform-d_1_ (CDCl_3_) as a solvent. Sample concentration was 10–20 mg/mL. Measurements were recorded at 300 K. Data processing was carried out with MestReNova (Version 9.0.1, Mestrelab Research S.L., Santiago de Compostela, Spain).

#### 2.6.3. Fourier Transform Infrared Spectroscopy

Fourier transform infrared spectroscopy (FTIR) was performed on a Bruker FTIR Vertex 70 (Bruker Optik GmbH, Ettlingen, Germany). Measuring software was Opus 7.5. All samples were measured in the wavenumber range of 6000–400 cm^−1^ with a resolution of 2 cm^−1^ and 32 scans. For temperature-dependent FTIR experiments, samples were prepared via solution casting of a crosslinked polymer film (0.1 mL of crosslinked polymer solution in DCM or anisole) onto a 6 mm by 1 mm potassium bromide plate. Films were dried for 16 h under vacuum at 40 °C. The temperature profile was chosen as follows: first, a heating and cooling cycle from 30 °C to 110 °C to 30 °C, and, then, a second heating and cooling cycle, from 30 °C to 150 °C to 30 °C, and, finally, from 30 °C to 190 °C to 30 °C. Temperature steps were 10 °C with a 15 min isothermal hold between successive steps in the temperature cycle to ensure equilibrium. A background measurement without a specimen in the sample holder was carried out at 30 °C and subtracted from the recorded data by the Bruker software OPUS. No additional data processing was implemented.

#### 2.6.4. Differential Scanning Calorimetry

To determine the glass transition temperature *T*_g_ of the homopolymers and the statistical copolymers as well as the opening and closing process of the Diels-Alder crosslinked polymer networks, differential scanning calorimetry (DSC) was carried out using a DSC 204 F1 Phoenix (NETZSCH-Gerätebau GmbH, Selb, Germany). Furthermore, 10–15 mg polymer or crosslinked polymer were weighed into an aluminum crucible, slightly pressed, and then closed afterwards. The measurements were performed at 1 bar under nitrogen atmosphere (flow rate of 20 mL/min) in the temperature range between −80 °C (for *n*-butyl acrylate containing copolymers) or −20 °C and 200 °C. Heating and cooling rates were 5 °C/min. During the first heating interval, the thermal history of samples was erased by heating up the samples from −80 or −20 °C to 110 °C, followed by cooling them down to −80 or −20 °C. In the second and third interval, they were heated to 150 °C. After cooling down to −80 or −20 °C, in the fourth interval, they were heated to 200 °C. The thermal properties were analyzed using the DSC data of the second, third, and fourth heating. Data processing was performed by Proteus analysis (NETZSCH-Gerätebau GmbH, Selb, Germany).

## 3. Results and Discussion

In this section, the results of the polymer synthesis as well as the various crosslinking processes are described and discussed. The crosslinked furfuryl bearing low molecular weight polymers are analyzed for their temperature-dependent, reversible behavior using temperature-dependent FTIR spectroscopy and differential scanning calorimetry (DSC). Detailed information about the monomer and polymer synthesis can be found in the Supporting Information (SI).

### 3.1. Polymer Synthesis

Polymers with different amounts of Diels-Alder motifs were synthesized by radical polymerization. For this purpose, a furfuryl-bearing monomer (FMA) as the diene motif and a maleimide-bearing monomer (MIMA) as the dienophile motif were selected as Diels-Alder groups. As a basic framework, an acrylate or a methacrylate unit is used, as this leads to polymers with a broad range of glass transition temperatures from well below the crosslinking temperature up to the retro-Diels-Alder reaction temperature, opening of the network, at around 120 °C. Moreover, both kinds of monomers are suitable for ATRP. To estimate the influence of the density of crosslinking groups, in addition to an FMA homopolymer, statistical copolymers with FMA as diene components were synthesized. Besides, the commercially available BMI as a statistical copolymer containing MIMA was synthesized as a dienophile component. Table 1 shows the polymers synthesized with their apparent molecular weights (*M*_n_, *M*_w_), their dispersity (*Ð*), their degree of functionalization with the respective Diels-Alder functional comonomer, (*D*_f_), and their glass transition temperature (*T*_g_, determined in the middle of the endothermic transition by DSC). The first three polymers were synthesized by ATRP. Since ATRP failed to achieve a sufficient molecular weight of the maleimide bearing copolymer, free radical polymerization was used for this copolymer with a very slightly increased dispersity index compared to the other copolymers synthesized by ATRP. The polymers are labelled as P(A_x_-*co*-B_y_)^z^ where A and B correspond to the respective abbreviations of the monomers. The subscripts x and y represent the weight percentage of the respective monomer in the polymer determined by ^1^H-NMR spectroscopy. The total apparent number averaged molecular mass of the polymer determined by size exclusion chromatography that is indicated by the superscript z in kDa.

### 3.2. Crosslinking of Furfuryl Bearing Polymers

The different ways of crosslinking carried out in this study are presented in Figure 7. In literature, the BMI crosslinker was used in a motif ratio of 1:2 (furfuryl-groups in polymer: maleimide groups in BMI) [30,43]. However, since there are two maleimide groups present in BMI, a ratio of 1:1 (furfuryl-groups in polymer: maleimide groups in BMI) should be sufficient. Therefore, different motif ratios were tested to observe an influence of the density of crosslinking groups. When crosslinking two different copolymers with each other, a motif ratio of 1:1 was used, with one carrying a furfuryl group and the other carrying a maleimide group. To find the appropriate crosslinking conditions for the different polymers, several routes were chosen. Besides the influence of temperature on the rate of crosslinking, the influence of density of crosslinking groups was investigated. Additionally, the influence of the glass transition temperature on the cyclo-reversion was investigated at the same time. The formation of a crosslinked structure could be visually confirmed by the finite swelling of the polymer network. Thus, all crosslinked polymers were first subjected to a swelling test. This was followed by temperature-dependent FTIR spectroscopy and differential scanning calorimetry to confirm the thermo-reversible behavior of the crosslinks.

In the next subchapters, the results of crosslinking for PFMA^5^, P(MMA_89_-*co*-FMA_11_)^24^ and P(BA_60_-*co*-FMA_40_)^10^ with BMI, as well as with P(MMA_74_-*co*-MIMA_26_)^9^, are described and discussed in terms of the influences of density of crosslinking groups and glass transition temperature. Furthermore, the self-healing capacity of the crosslinked polymers will be discussed.

#### 3.2.1. Visual Observation of Gelation

Table 2 shows the results of the visual gelation test of the furfuryl bearing polymers. In addition to the parameters of the crosslinking reaction such as the motif ratio between diene and dienophile, reaction time, and crosslinking temperature, the visual appearance of the systems is described.

**Figure 8:**
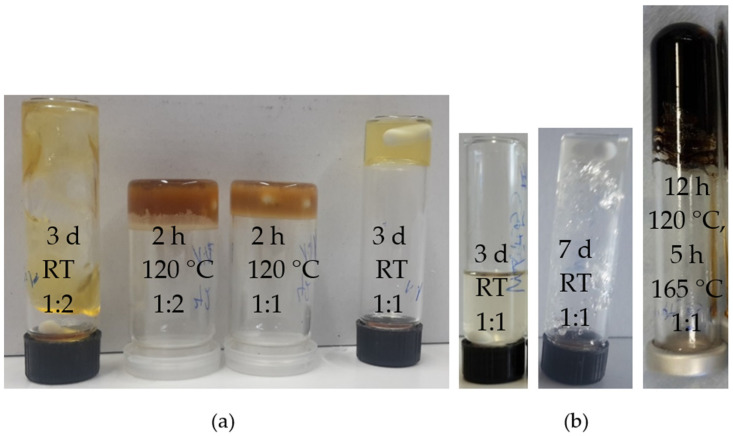
Crosslinked PFMA^5^. (**a**) PFMA^5^ with BMI crosslinked at two different temperatures (room temperature (RT) and 120 °C) and two different motif ratios (1:2 and 1:1) in DCM. (**b**) Crosslinked PFMA^5^ with P(MMA_74_-*co*-MIMA_26_)^9^ at two different temperatures (room temperature (RT) in DCM and 120 °C; 165 °C in anisole) and the gelation process at room temperature in DCM after 3 days and 7 days.

Figure 8 shows the crosslinked samples of the PFMA^5^. All other images of the crosslinked furfuryl bearing polymers can be found in the SI.

When looking at the results of the crosslinked polymer networks, it becomes apparent that all investigated motif ratios between diene and dienophile lead to a crosslinked polymer network whether a crosslinker molecule such as BMI is used or the dienophile bearing copolymer (P(MMA_74_-*co*-MIMA_26_)^9^). Figure 8a shows that there is a visible difference in the crosslinked PFMA^5^ with BMI for the different motif ratios at room temperature. Thus, the gel formed at a motif ratio of 1:2 (one diene group to two dienophile groups) is less stable (i.e., less crosslinked) than the gel formed at a motif ratio of 1:1 (one diene group to one dienophile group). This is evident from the flowability of the gel with a motif ratio of 1:2 at room temperature. This suggests that a stoichiometric ratio of the two functional groups (1:1) is the better choice for crosslinking, as a maximum number of crosslinks can be achieved, even if not a full conversion of the functional groups may be obtained due to topological confinements by the chains. If a ratio of 1:2 is used, half of the dienophile groups remain unreacted, thus, leading to fewer crosslinks. Furthermore, not all samples show a swollen crosslinked product. Some products showed gelation only after some solvent had evaporated.

A difference can be seen here in the density of crosslinking groups. The crosslinked PFMA^5^, which has a degree of functionalization of 100 wt% and, thus, the highest percentage of diene motifs in the polymers studied, shows a gel under all investigated crosslinking conditions, even without removal of the solvent. One explanation for this could be the high proportion of diene motifs, which enables the formation of a percolating network already at low conversion of functional groups. In contrast, the P(MMA_89_-*co*-FMA_11_)^24^ has the lowest proportion of diene motifs in the test series with only 11 wt%. The comparison among each other shows no differences in the motif ratio of BMI, but a difference in the viscosity of the crosslinked P(MMA_89_-*co*-FMA_11_)^24^. While the P(MMA_89_-*co*-FMA_11_)^24^ crosslinked with BMI treated at room temperature remained a liquid, the P(MMA_89_-*co*-FMA_11_)^24^ crosslinked with BMI at 120 °C formed a swollen gel. This may be explained by the kinetics, which is slower at room temperature compared to 120 °C. However, the sample crosslinked with P(MMA_74_-*co*-MIMA_26_)^9^ at room temperature is the only one that shows a very stable gel after only three days, despite the low density of crosslinking groups. This may be explained by the fact that crosslinking between two different copolymers occurs more easily compared to crosslinking of a low-functional copolymer with a small molecule like BMI, due to entropic confinements. In contrast, the P(MMA_89_-*co*-FMA_11_)^24^ crosslinked with P(MMA_74_-*co*-MIMA_26_)^9^ after heating (165 °C), even compared to the crosslinked PFMA^5^ with P(MMA_74_-*co*-MIMA_26_)^9^, shows no gelation. However, if part of the solvent evaporated, a clear gel is formed at room temperature. With a degree of functionalization of 40 wt%, P(BA_60_-*co*-FMA_40_)^10^ has an intermediate density of crosslinking groups. In contrast to the previous crosslinked PFMA^5^, the BMI crosslinked P(BA_60_-*co*-FMA_40_)^10^ did not show gelation at room temperature but remained liquid. After removing the solvent, the sample could not be redissolved. Therefore, gelation had occurred. In the case of P(BA_60_-*co*-FMA_40_)^10^ crosslinked with P(MMA_74_-*co*-MIMA_26_)^9^), the first comparison shows a color difference between the crosslinking at higher temperatures and at room temperature. No gelation is observed after three days for the copolymers crosslinked at room temperature, but, if the system is stirred further, a gel is formed after seven days. The same observation could be made for the crosslinking process of PFMA^5^. The polymer that has been crosslinked at higher temperatures shows no gelation after the reaction time has elapsed, but, if part of the solvent evaporated, the product forms a gel at room temperature. In this case, a particular swollen crosslinked polymeric network seems to be present.

Another observation that can be made is that the crosslinking temperature has a major influence on the crosslinking process, i.e., the Diels-Alder reaction. This shows that the Diels-Alder reaction can be accelerated by increasing the reaction temperature. It can be stated that the higher the reaction temperature is, the faster the reaction is, regardless of the number of crosslinks. However, it can also be seen that, if the reaction temperature is too high for an extra-long period, the crosslinked polymeric network can decompose. For example, the case of the PFMA^5^ crosslinked with the P(MMA_74_-*co*-MIMA_26_)^9^ in Figure 8b shows a yellow to dark brown/black color at a temperature of 165 °C. From these studies, it follows that the crosslinking reaction can be controlled in time with help of the reaction temperature. The higher the reaction temperature is, the faster the Diels-Alder reaction is. For the reaction system of furan and maleimide, a crosslinking temperature of 120 °C has proven to be optimal.

#### 3.2.2. Thermo-Reversibility of Gelation

In this part, the thermo-reversibility of the Diels-Alder reaction in the networks is studied by DSC and temperature-dependent FTIR. An opening of the Diels-Alder system (retro-Diels-Alder reaction) can be observed in the DSC by an endothermic peak in the heating curve at around 120 °C for the furfuryl–maleimide system. Ideally, the reoccurring Diels-Alder reaction causes an exothermic peak in the following cooling curve. In addition to DSC, the crosslinked polymers were investigated by temperature-dependent FTIR spectroscopy. This method is very sensitive for specific absorptions of the different molecular structures formed by the Diels-Alder and retro-Diels-Alder reaction. In addition, in a step-wise changing temperature profile at each temperature, the sample can be equilibrated, which was achieved by keeping the sample for 15 min before measuring the spectrum. The sample was heated above the retro-Diels-Alder reaction temperature of 120 °C and cooled down by applying the step-wise temperature protocol twice to demonstrate that several crosslinking cycles of the system are possible. The spectra of the crosslinked polymers show changes of the absorptions at 1750 cm^−1^, 1510 cm^−1^, and between 1050 and 550 cm^−1^ (SI). The wavenumber range from 1750 cm^−1^ to 1050 cm^−1^ shows four main absorption bands at 1750 cm^−1^, 1510 cm^−1^, 1380 cm^−1^, and a double band at 1180 and 1140 cm^−1^. The absorption at 1750 cm^−1^ is assigned to the stretching vibration of the carbonyl group. The absorption band at 1510 cm^−1^ can be assigned to the stretching vibration of the carbon-carbon double bond. The absorption at 1380 cm^−1^ corresponds to the stretching vibration of the carbon-nitrogen bond of the maleimide ring. The double absorption bands at 1180 cm^−1^ and 1140 cm^−1^ can be assigned to the carbon-oxygen stretching vibration of the ether bridgehead as well as the anhydride vibration. The ether vibration decreases with an increasing temperature. This is due to the retro-Diels-Alder reaction. The bicyclic system is broken down and the furfuryl ring is formed. This changes the chemical environment of the ether bond and, thus, influences its absorption. Since the same absorption band becomes more intense again when cooling, it can be assumed that a Diels-Alder reaction has taken place. The wavenumber range from 1050 to 550 cm^−1^ supports the statement of thermo-reversibility of the Diels-Alder polymer system. Here, the wavenumbers at 980 and 880 cm^−1^, which can be assigned to the double bond deformation mode, as well as the wavenumbers of the free maleimide ring (845, 710, and 680 cm^−1^) are decisive for this statement. The deformation mode of the free maleimide ring only becomes apparent after the system has been heated. At the same time, the intensity of the signals decreases again when the system cools down.

**Figure 9:**
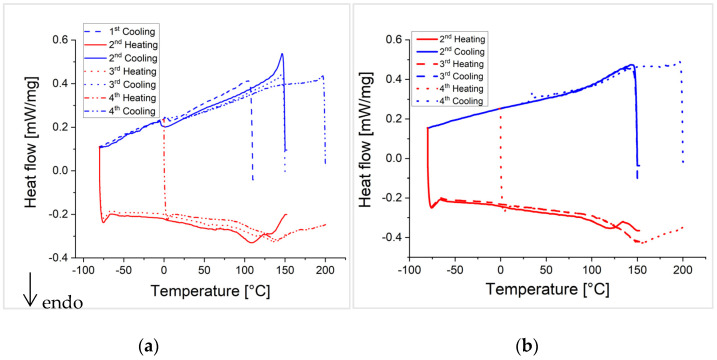
DSC heating (red) and cooling (blue) curves of the crosslinked P(BA_60_-*co*-FMA_40_)^10^. (**a**) Crosslinked P(BA_60_-*co*-FMA_40_)^10^ with BMI in a motif ratio of 1:1. The endothermic peak indicates the retro-Diels-Alder reaction at around 90 °C in all three heating curves. (**b**) Crosslinked P(BA_60_-*co*-FMA_40_)^10^ with P(MMA_74_-*co*-MIMA_26_)^9^. The endothermic peak indicates the retro-Diels-Alder reaction starting at around 100 °C in the third and fourth heating curve.

**Figure 10:**
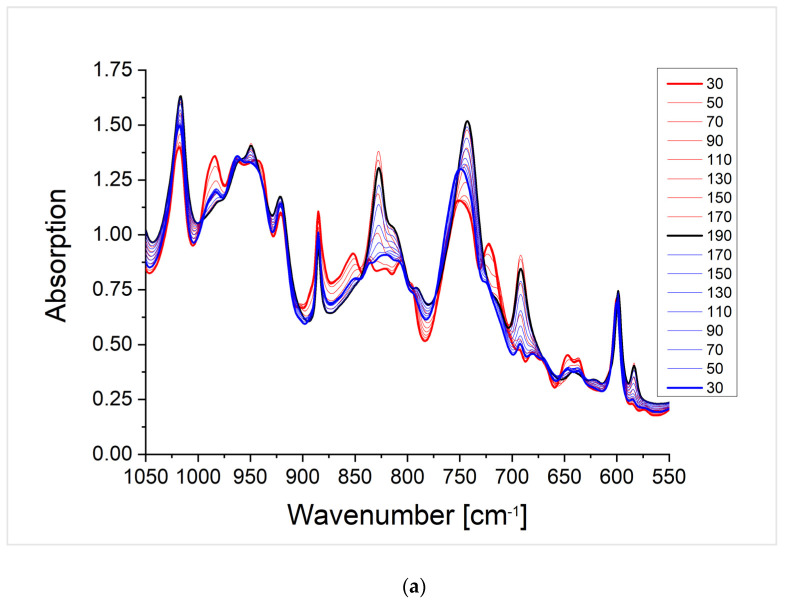

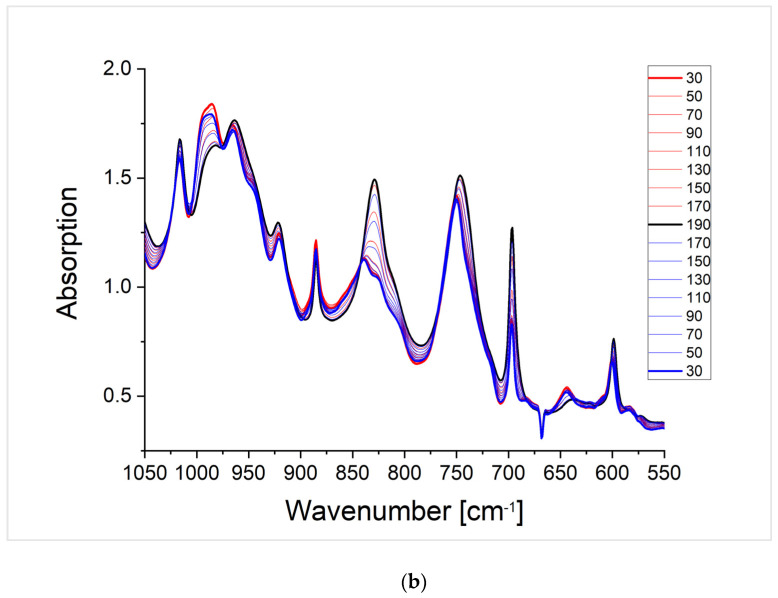
Temperature dependent FTIR spectra of the crosslinked P(BA_60_-*co*-FMA_40_)^10^ with BMI (**a**) and with P(MMA_74_-*co*-MIMA_26_)^9^ (**b**). Spectra of the heating cycle (red), spectrum of the maximum temperature (black), and spectra of the cooling cycle (blue). The entire temperature cycle from 30-190-30 °C in the wavenumber range from 1050 to 550 cm^−1^ is shown, as the ring deformation mode of the maleimide ring formed in the retro-Diels-Alder reaction can be easily observed.

**Figure 11:**
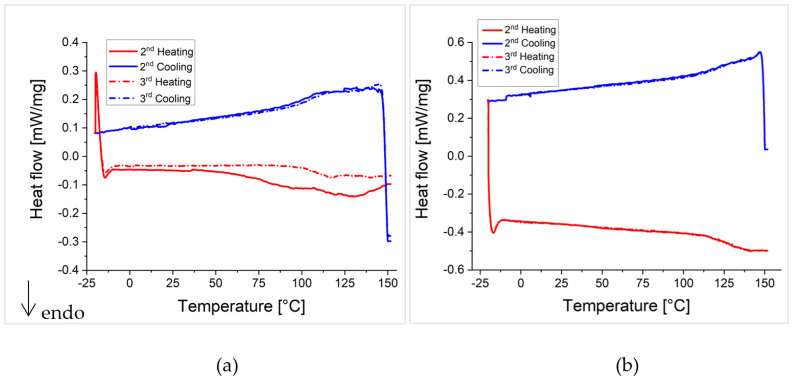
DSC heating (red) and cooling (blue) curves of the crosslinked P(MMA_89_-*co*-FMA_11_)^24^. (**a**) Crosslinked P(MMA_89_-*co*-FMA_11_)^24^ with BMI in a motif ratio of 1:1. The endothermic step indicates the glass transition temperature around 110 °C in the 3^rd^ heating curve. (**b**) Crosslinked P(MMA_89_-*co*-FMA_11_)^24^ with P(MMA_74_-*co*-MIMA_26_)^9^. The glass transition of the two copolymers occurs at around 120 °C.

**Figure 12:**
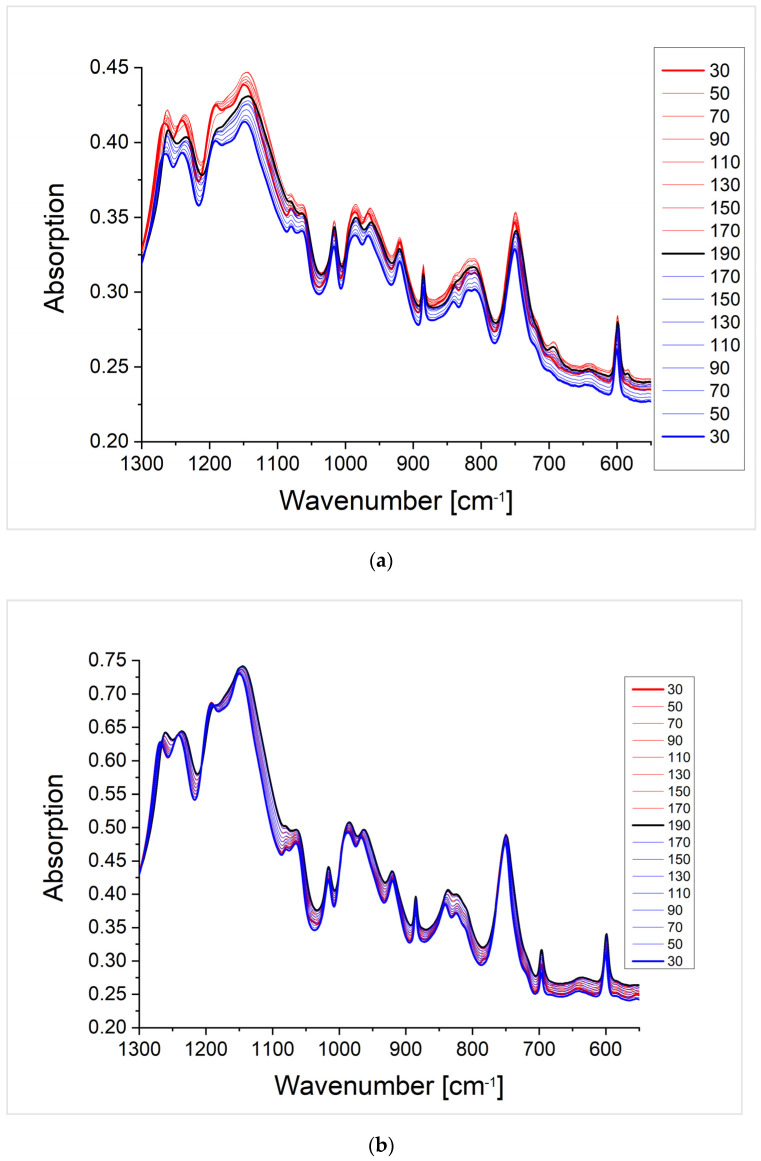
Temperature dependent FTIR spectra of the crosslinked P(MMA_89_-*co*-FMA_11_)^24^ with BMI (**a**) and P(MMA_74_-*co*-MIMA_26_)^9^ (**b**). Spectra of the heating cycle (red), spectrum of the maximum temperature (black), and spectra of the cooling cycle (blue). The entire temperature cycle from 30-190-30 °C is shown in the wavenumber range from 1300 to 550 cm^−1^, as, here, the ring deformation mode of the maleimide ring formed in the retro-Diels-Alder reaction can be observed.

Since the same temperatures in the DSC curves as well as in the FTIR spectra are of interest for all crosslinked polymer networks, the results of the investigations of P(MMA_89_-*co*-FMA_11_)^24^ with a high glass transition temperature (109 °C) and a low degree of functionalization (11 wt%) as well as P(BA_60_-*co*-FMA_40_)^10^ with a low glass transition temperature (−17 °C) and an intermediate degree of functionalization (40 wt%) crosslinked with BMI and P(MMA_74_-*co*-MIMA_26_)^9^ are shown in the following Figure 9, Figure 10, Figure 11 and Figure 12 and will be discussed in detail. The results of the crosslinked PFMA^5^, as well as all other spectra of P(MMA_89_-*co*-FMA_11_)^24^ and P(BA_60_-*co*-FMA_40_)^10^, can be found in the Supporting Informations (SI).

The crosslinked polymer systems where the glass transition temperature is below (Figure 9 P(BA_60_-*co*-FMA_40_)^10^) or near the Diels-Alder reaction temperature (Figures S35–S39 in the SI; PFMA^5^), show the dissociation of the network by the retro-Diels-Alder reaction, which is indicated by an endothermic peak in all heating curves of the DSC measurements. The DSC measurements of P(BA_60_-*co*-FMA_40_)^10^ crosslinked with BMI or P(MMA_74_-*co*-MIMA_26_)^9^ show Diels-Alder reactions upon cooling in all cycles (Figure 9). This is likely due to the low glass transition temperature of these systems. In the case of the crosslinked PFMA^5^, a Diels-Alder reaction can be observed only in some DSC cooling curves (e.g., Figure S36 4th cooling curve in the SI). This indicates that even during a slightly higher glass transition temperature than the reaction temperature, crosslinking is affected. The reversibility of the Diels-Alder reaction is also confirmed by temperature-dependent FTIR spectra shown for the two crosslinked P(BA_60_-*co*-FMA_40_)^10^) systems in Figure 10 and for the PFMA^5^ in the SI. It is shown that the change in the absorption intensity of the crosslinked PFMA^5^ (Figure S42 in the SI) indicates a lower mobility compared to the spectra of the P(BA_60_-*co*-FMA_40_)^10^ (Figure 10) with a lower glass transition temperature because of the high density of crosslinking groups of the PFMA^5^.

In contrast, the DSC measurements of the crosslinked polymer system with a glass transition temperature well above the crosslinking temperature show an endothermic transition only in the first heating cycle of the BMI crosslinked P(MMA_89_-*co*-FMA_11_)^24^ (Figure 11a). The other heating curves (Figure 11b) do not show a retro-Diels-Alder reaction, what indicates a suppression of the Diels-Alder reaction during cooling by the glass transition, which occurs at a high temperature. Note that the initial crosslinking of all systems took place in the presence of a solvent, which means that all systems were well above the glass transition temperature during the first crosslinking. Therefore, only the glass transition temperature can be observed in Figure 11. In addition, the temperature-dependent FTIR spectra in Figure 12 display only a weak change of the characteristic bands when compared to Figure 10. In the case of P(BA_60_-*co*-FMA_40_)^10^, a thermo-reversibility can be observed by the corresponding FTIR absorption bands in both the BMI and the P(MMA_74_-*co*-MIMA_26_)^9^ crosslinked samples (Figure 10). The P(BA_60_-*co*-FMA_40_)^10^ crosslinked with BMI shows an even better de-crosslinking and crosslinking than P(BA_60_-*co*-FMA_40_)^10^ crosslinked with P(MMA_74_-*co*-MIMA_26_)^9^. Here, absorption bands increase and decrease with an increasing temperature and reach their previous intensity with falling temperature again. This result supports the statement that thermo-reversibility strongly depends on the glass transition temperature. To further check the influence of the glass transition temperature on the reversibility of the Diels-Alder reaction in this system, various amounts of anisole as a plasticizer were added and it could be confirmed by DSC that the Diels-Alder reaction became reversible when the system was sufficiently plasticized (results shown in SI).

One observable difference in the DSC measurements of the crosslinked polymers is the onset of the endothermic transition, i.e., the retro-Diels-Alder reaction. The onset of the retro-Diels-Alder reaction in P(MMA_74_-*co*-MIMA_26_)^9^ crosslinked P(BA_60_-*co*-FMA_40_)^10^ occurs at a lower temperature compared to PFMA^5^. In addition, the temperature position of the maxima of the endothermic transitions (maximum heat flow) are different. It occurs at 140 °C for the BMI crosslinked PFMA^5^, while it is located at 160 °C for the P(MMA_74_-*co*-MIMA_26_)^9^ crosslinked PFMA^5^. A reason for this difference can be the influence of the higher glass transition temperature of the P(MMA_74_-*co*-MIMA_26_)^9^, which shifts the maximum heat flow to higher temperatures. For P(BA_60_-*co*-FMA_40_)^10^, the maximum heat flow shifts by 10 °C to 140 °C for the BMI crosslinked copolymer and to 150 °C for the crosslinked P(MMA_74_-*co*-MIMA_26_)^9^ in contrast to the maximum heat flow of the analyzed crosslinked PFMA^5^. Next to the influence of the glass transition temperature, the influence of the density of crosslinking groups is discussed. The comparison of the DSC curves of the two PFMA^5^ crosslinked with BMI (Figures S35 and S36 in the SI), as well as those crosslinked with P(MMA_74_-*co*-MIMA_26_)^9^, shows that the third heating curve in case of the PFMA^5^ crosslinked with a motif ratio of 1:1 drops significantly more than for the other two samples. This indicates that a higher density of crosslinking groups relate to a higher crosslink density, which, in turn, leads to a steeper course and larger endothermic transition when the crosslinks open upon heating. Since the DSC curves (Figure 11) of crosslinked P(MMA_89_-*co*-FMA_11_)^24^ show no opening (retro-Diels-Alder reaction) except for one of the heating curves (Figure 11b, 2nd heating curve), no statement about the endothermic transition can be made. The comparison of the DSC curves of P(BA_60_-*co*-FMA_40_)^10^ (Figure 9) shows that the retro-Diels-Alder reaction can be observed somewhat earlier at 100 °C instead of 120 °C for crosslinked PFMA^5^, but also a flatter endothermic transition peak can be observed. Given the lower density of crosslinking groups of the crosslinked P(BA_60_-*co*-FMA_40_)^10^, this flatter curve indicates that the density of crosslinking groups has an influence on the course of the heating curve. By comparing the DSC measurements of the different polymeric systems, it can be seen that the glass transition temperature of the polymer has a higher influence on the thermo-reversibility of the Diels-Alder reaction, than the density of crosslinking groups. However, these measurements also show that it is difficult to observe the renewed Diels-Alder reaction in the DSC cooling curve through an exothermic transition peak.

#### 3.2.3. Self-Healing Ability of Crosslinked Furfuryl Bearing Polymers

The previous results on the thermo-reversibility of the Diels-Alder reaction between furan and maleimide should show whether the polymers of this study could serve as self-healing crosslinked materials. Therefore, the relative amount of thermo-reversibility of the Diels-Alder reaction was estimated from the retro-Diels-Alder endothermic peak in the DSC traces. The second and third heating cycles were chosen for calculation of the self-healing capacity. More precisely, the second and third heating cycle were used, as they displayed the system with an erased thermal history and erased influence of the solvent present during the sample preparation. The self-healing capacity was determined from the ratio of the endothermic transitions of the third and second heating cycles. A ratio of one indicates a complete healing of the crosslinked system. The following Table 3 shows the results of all crosslinked bulk polymer systems and some plasticized polymer systems of this study.

**Figure 13:**
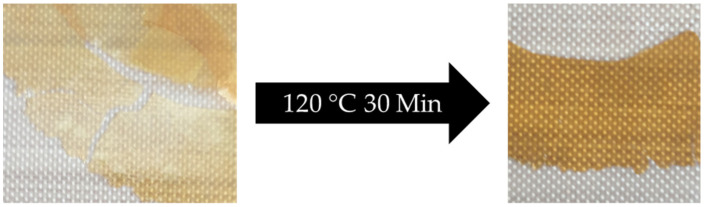
Reshaping of cracked P(BA_60_-*co*-FMA_40_)^10^ crosslinked with BMI with a motif ratio of 1:1 at room temperature. Broken polymeric film on the **left side** and cured polymeric film on the **right side**.

A comparison of the self-healing capacities shows that the crosslinked polymers with the highest flexibility also show the highest self-healing capacities, independent of the crosslinking agent used. In addition to the PFMA^5^ crosslinked with BMI at high temperatures, the crosslinked P(BA_60_-*co*-FMA_40_)^10^ shows the highest self-healing capacities with a ratio of 1. As an example, the self-healing ability of BMI crosslinked P(BA_60_-*co*-FMA_40_)^10^ at room temperature with a motif ratio of 1:1 is shown in Figure 13.

The samples of the crosslinked P(MMA_89_-*co*-FMA_11_)^24^, on the other hand, show no self-healing, except for two samples measured with a plasticizer. Here, the samples with 10 and 25 wt% of anisole show a self-healing capacity of 0.84 and 0.6. These values are lower than the values that can be achieved with a crosslinked polymer with a lower glass transition temperature (P(BA_60_-*co*-FMA_40_)^10^) or a higher density of crosslinking groups (PFMA^5^). When looking at the results of the swelling test, it is noticeable that the samples with higher amounts of plasticizer (25 and 50 wt% anisole) have a lower self-healing capacity (0.6 and 0). Actually, it should be assumed that, with a higher proportion of plasticizer, the mobility of the chains increases and, thus, also the self-healing capacity. This could not be observed. In contrast, the sample with the highest anisole content (50 wt%) shows a similar thermal behavior in DSC like the unswollen samples of crosslinked P(MMA_89_-*co*-FMA_11_)^24^. Further investigations showed, however, that the crosslinked P(MMA_89_-*co*-FMA_11_)^24^ can only adsorb a certain amount of solvent. Thus, for the samples with a higher proportion of anisole, the swollen crosslinked polymer is present besides an excess of anisole. This excess of anisole dominates the heat flow and, thus, it is not possible to observe the behavior of the crosslinked and swollen P(MMA_89_-*co*-FMA_11_)^24^ (Figure S51 in the SI). Clearly, the mobility of the polymer backbone influences the self-healing. A higher self-healing capacity can be achieved if the polymer chains or the crosslinking agent exhibit a certain degree of mobility.

## 4. Conclusions

Thermo-reversible Diels-Alder polymer networks were prepared by following two different crosslinking paths. On the one hand, furfuryl bearing polymers were crosslinked with BMI and, on the other hand, with a maleimide bearing multifunctional polymer. Temperature-dependent FTIR spectroscopy and differential scanning calorimetry showed that the glass transition temperature has a major influence on the thermo-reversibility of the Diels-Alder reaction. In polymers with a glass transition temperature above room temperature (e.g., P(MMA_89_-*co*-FMA_11_)^24^), only the retro-Diels-Alder reaction could be observed during the first heating. In contrast, thermo-reversible behavior was observed in crosslinked polymers with a glass transition temperature below room temperature (e.g., P(BA_60_-*co*-FMA_40_)^10^). However, if a plasticizer was added to crosslinked P(MMA_89_-*co*-FMA_11_)^24^ to decrease the glass transition temperature, the thermo-reversible behavior of the Diels-Alder reaction could be observed. In order to enable thermo-reversibility in materials with a higher glass transition temperature, a diene or dienophile system with a correspondingly higher crosslinking and de-crosslinking temperature is required, such as an anthracene motif. The results presented in this study support the statement of Kickelbick and Zeng that the mobility of the chains (i.e., the glass transition temperature) or the crosslinker have an influence on the thermo-reversibility of the Diels-Alder system [36,37,38]. Contrary to the strong influence of the glass transition temperature, it was also shown that no great influence of the density of crosslinking groups on thermo-reversibility can be observed. Crosslinking occurred faster at higher temperatures, as long as they are below the retro-Diels-Alder reaction temperature (120 °C for furfuryl and maleimide). This study successfully demonstrates that the formation of thermo-reversible polymer networks is easy by introducing Diels-Alder motifs to suitable (co)polymers, which are accessible by any suitable (co)polymerization such as free radical or controlled radical polymerization.

## Figures and Tables

**Table 1:** Molecular weight (*M*_n_/*M*_w_), dispersity (*Ð*), degree of functionalization (*D*_f_), and glass transitions temperature (*T*_g_, determined in the middle of the endothermic transition) of the three different furfuryl-bearing polymers and the maleimide-bearing copolymer.

Polymer	*M*_n_ [kDa]	*M*_w_ [kDa]	*Ð*	*D*_f_ [wt%]	*T*_g_ [°C]
PFMA^5^	4.7	6.6	1.4	100	39
P(MMA_89_-*co*-FMA_11_)^24^	24.0	40.0	1.7	11	109
P(BA_60_-*co*-FMA_40_)^10^	10.4	18.1	1.7	40	−17
P(MMA_74_-*co*-MIMA_26_)^9^	9.0	16.2	1.8	26	135

**Table 2:** Conditions for the crosslinking of the diene bearing components (PFMA^5^, P(MMA_89_-*co*-FMA_11_)^24^, P(BA_60_-*co*-FMA_40_)^10^) with one of the dienophile-bearing components (BMI, P(MMA_74_-*co*-MIMA_26_)^9^) such as a motif ratio between diene and dienophile, rection time, and crosslinking temperature as well as visual gelation and color of the product. Gelation results marked with a star only showed gelation after removal of the solvent used during the crosslinking reaction.

**Crosslinking** **Components**	**PFMA^5^ + BMI**				**PFMA^5^ + P(MMA_74_-*co*-MIMA_26_)^24^**			
**Motif Ratio**	**1:2**		**1:1**		**1:1**			
Reaction Temperature [°C]	25	120, 25	25	120, 25	25		120, 165	
Reaction time [h]	72	2, 12	72	2, 12	168		12, 5	
solvent	DCM	DCM	DCM	DCM	DCM		anisole	
gelation	+	+	+	+	+		+	
color	yellow	caramel, turbid	yellow	caramel, turbid	light yellow		black/dark brown	
**Crosslinking** **Components**	**P(MMA_89_-*co*-FMA_11_)^9^ + BMI**						**P(MMA_89_-*co*-FMA_11_)^9^ + P(MMA_74_-*co*-MIMA_26_)^24^**	
**Motif Ratio**	**1:2**		**1:1**		**1:0.5**		**1:1**	
Reaction Temperature [°C]	25	120, 25	25	120, 25	25	120, 25	25	120, 165
Reaction time [h]	72	2, 12	72	2, 12	72	2, 12	72	12, 5
solvent	DCM	DCM	DCM	DCM	DCM	DCM	DCM	anisole
gelation	+ *	+	+ *	+	+ *	+	+	+ *
color	light yellow	yellow	light yellow	yellow	light yellow	yellow	light yellow, turbid	yellow
**Crosslinking** **Components**	**P(BA_60_-*co*-FMA_40_)^10^ + BMI**						**P(BA_60_-*co*-FMA_40_)^10^ + P(MMA_74_-*co*-MIMA_26_)^24^**	
**Motif Ratio**	**1:2**		**1:1**		**1:0.5**		**1:1**	
Reaction Temperature [°C]	25	120, 25	25	120, 25	25	120, 25	25	120, 165
Reaction time [h]	72	2, 12	72	2, 12	72	2, 12	168	12, 5
solvent	DCM	DCM	DCM	DCM	DCM	DCM	DCM	anisole
gelation	+ *	+ *	+ *	+ *	+ *	+ *	+	+ *
color	light yellow	light yellow	light yellow	light yellow	light yellow	light yellow	light yellow	dark yellow

**Table 3:** Ratios of the endotherms of the retro-Diels-Alder reaction during the second and third heating.

Polymer with Crosslinking Agent and Conditions	rDA_3_/rDA_2_
PFMA^5^ with BMI; 1:1 at rt	0.90
PFMA^5^ with BMI; 1:2 at rt	0.91
PFMA^5^ with BMI; 1:1 at 120 and 165 °C	1.0
PFMA^5^ with BMI; 1:2 at 120 and 165 °C	1.0
PFMA^5^ with P(MMA_74_-*co*-MIMA_26_)^9^; 1:1 at rt	0.89
P(MMA_89_-*co*-FMA_11_)^24^ with BMI; 1:1 at rt	0
P(MMA_89_-*co*-FMA_11_)^24^ with BMI; 1:1 at 120 °C	0
P(MMA_89_-*co*-FMA_11_)^24^ with BMI; 1:1 at rt; 5 wt% anisole	0
P(MMA_89_-*co*-FMA_11_)^24^ with BMI; 1:1 at rt; 10 wt% anisole	0.84
P(MMA_89_-*co*-FMA_11_)^24^ with BMI; 1:1 at rt; 25 wt% anisole	0.60
P(MMA_89_-*co*-FMA_11_)^24^ with BMI; 1:1 at rt; 50 wt% anisole	0
P(MMA_89_-*co*-FMA_11_)^24^ with P(MMA_74_-*co*-MIMA_26_)^9^; 1:1 at rt	0
P(BA_60_-*co*-FMA_40_)^10^ with BMI; 1:1 at rt	1.0
P(BA_60_-*co*-FMA_40_)^10^ with BMI; 1:2 at rt	0.98
P(BA_60_-*co*-FMA_40_)^10^ with P(MMA_74_-*co*-MIMA_26_)^9^; 1:1 at rt	0.99

## Supplementary Materials

The following are available online at https://www.mdpi.com/article/10.3390/polym13081189/s1, Figures S1–S6: ^1^H, ^13^C-NMR spectra of the furan protected maleimide methacrylate monomer synthesis (MIMA, 1–3) in CDCl_3_, S7–S13: ^1^H-NMR spectra, SEC measurements, DSC measurements and determination of the degree of functionalization of the polymers bearing furfuryl groups, S14–S18: ^1^H-NMR spectra, SEC measurements, DSC measurements and determination of the degree of functionalization of the polymer bearing maleimide groups, S19–S21: DSC measurement, ^1^H-NMR spectra and FTIR spectra of the deprotection of the maleimide bearing copolymer, S22–S23: Visual observations of gelation of the other copolymers, S24–S34: DSC and FTIR spectra of the crosslinked P(BA_60_-*co*-FMA_40_)^10^, S35–S42: DSC and FTIR spectra of the crosslinked PFMA^5^, S43–S49: DSC and FTIR spectra of the crosslinked P(MMA_89_-*co*-FMA_11_)^24^, S50–S51: DSC measurements of the swelling test with the plasticizer anisole. Table S1: Glass transition temperatures (*T*_g_) determined by onset and in the middle of the endothermic transition of the three different furfuryl bearing polymers, Table S2: Glass transition temperature (*T*_g_) determined by onset and in the middle of the endothermic transition of the maleimide bearing copolymer.
