# Supplementary material for: Influence of the Glass Transition Temperature and the Density of Crosslinking Groups on the Reversibility of Diels-Alder Polymer Networks
Source: Polymers (Basel). 2021 Apr 7;13(8):1189. doi: 10.3390/polym13081189 (PMC8067813; doi:10.3390/polym13081189)
Supplement: Supplementary file 1 [file polymers-13-01189-s001.pdf]

Supporting Information

Influence of the Glass Transition Temperature and the Density of Crosslinking Groupson the Reversibility of Diels-Alder Polymer Networks.

Merlina Thiessen[a], Volker Abetz[a,b]

[a] Institute of Physical Chemistry, Universität Hamburg, Grindelallee 117, 20146 Hamburg, Germany; merlina.thiessen@chemie.uni-hamburg.de
[b] Institute of Membrane Research, Helmholtz-Zentrum Hereon, Max-Planck-Straße 1, 21502 Geesthacht, Germany

Correspondence to: Volker Abetz (E-mail: volker.abetz@hereon.de).

1. Monomer Synthesis

^{1}H, ^{13}C-NMR spectra of the furan protected maleimide methacrylate monomer synthesis (MIMA,1–3).

MIMA-1

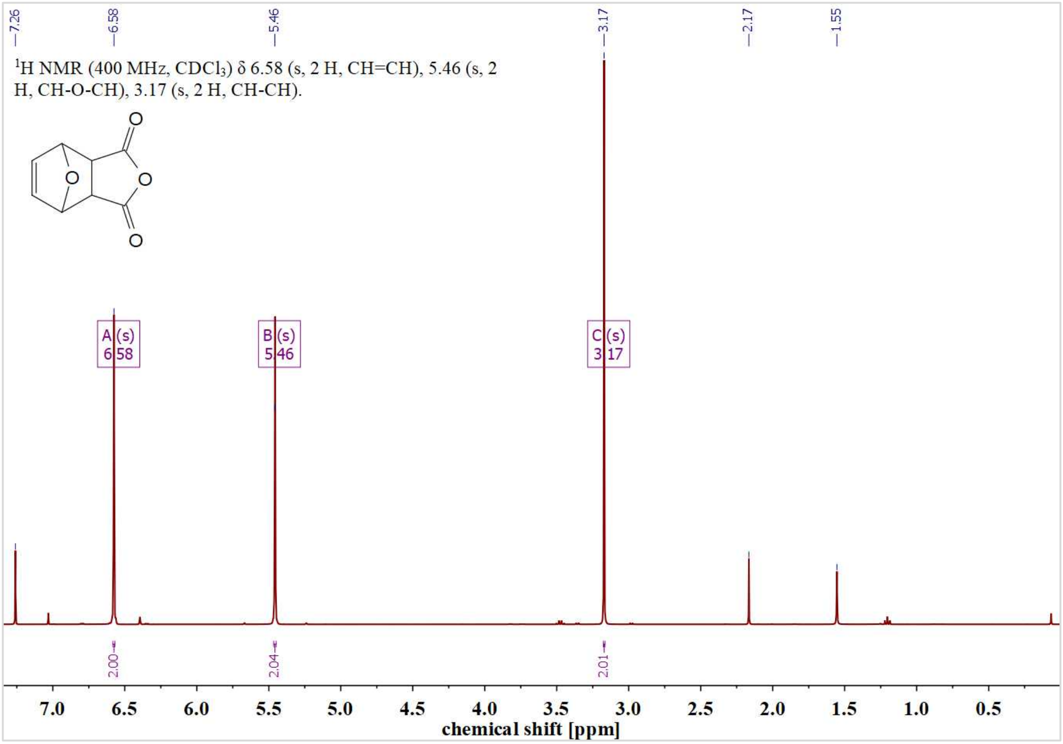

Figure S1. ^{1}H-NMR spectrum of the compound **1** of the furan protected maleimide methacrylate monomer in $CDCl_3$.

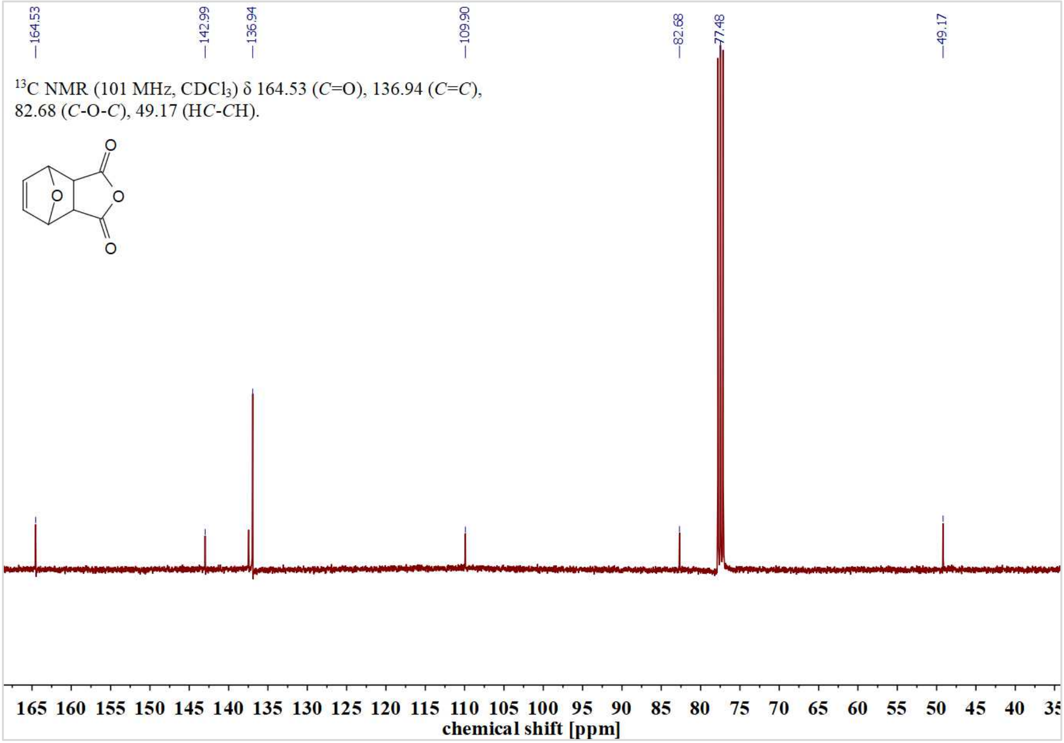

Figure S2. ^{13}C-NMR spectrum of the compound **1** of the furan protected maleimide methacrylate monomer in $CDCl_3$.

MIMA-2

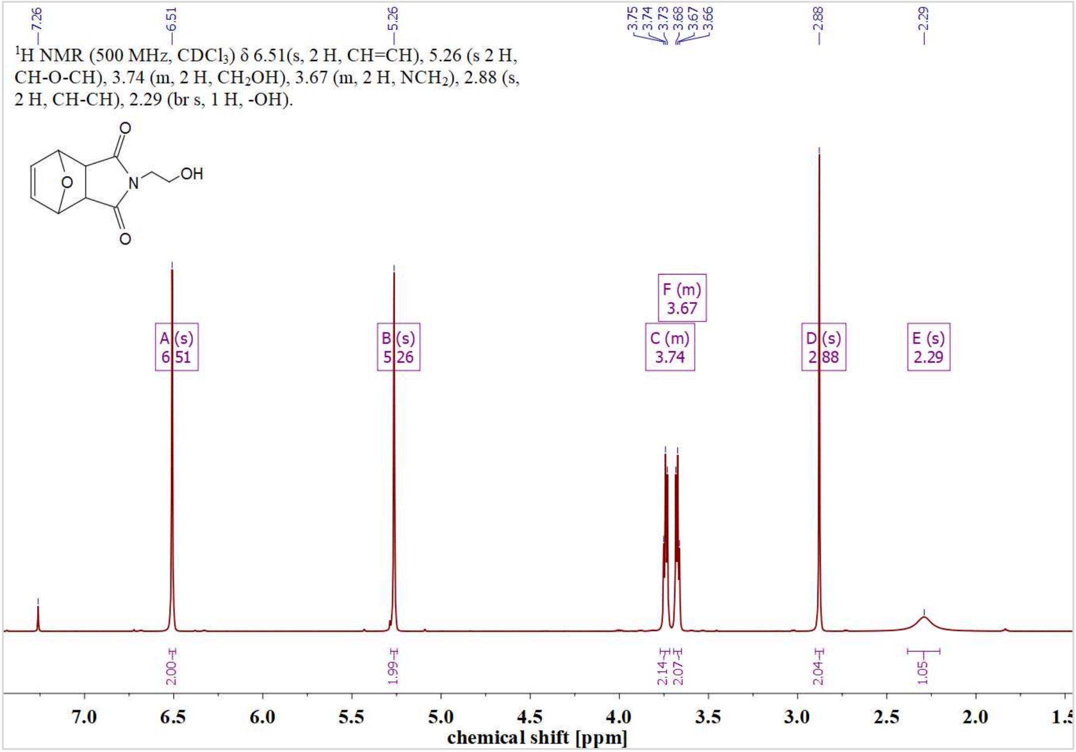

Figure S3. ^{1}H-NMR spectrum of the compound **2** of the furan protected maleimide methacrylate monomer in $CDCl_3$.

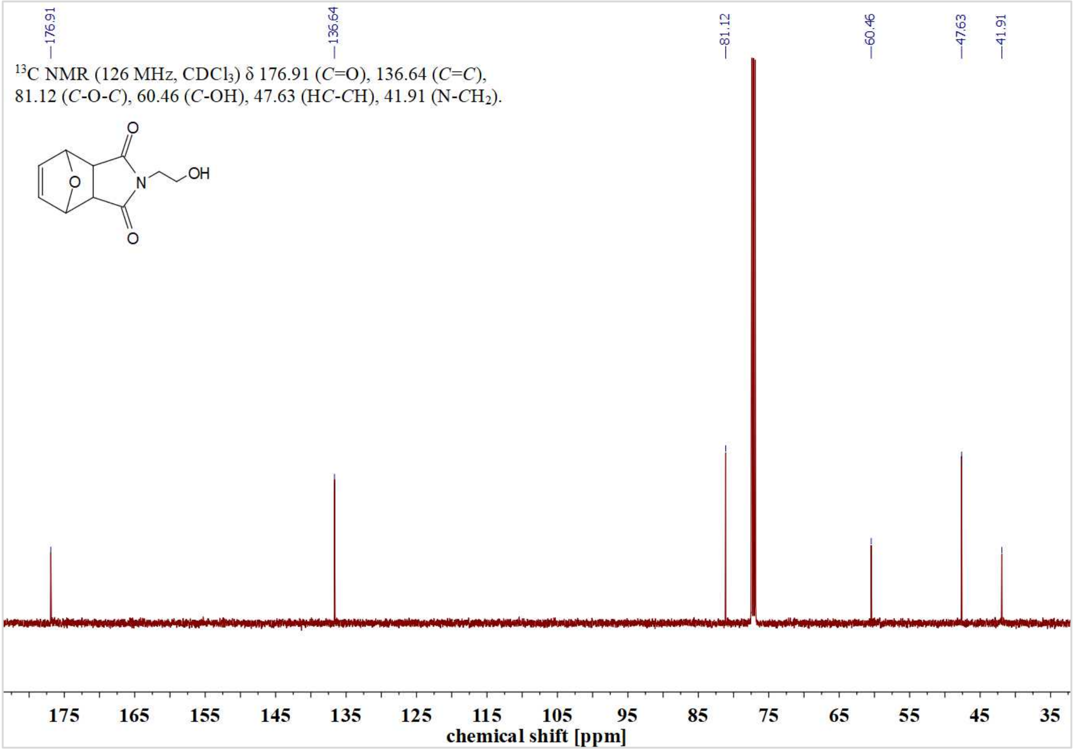

Figure S4. ^{13}C-NMR spectrum of the compound **2** of the furan protected maleimide methacrylate monomer in $CDCl_3$.

MIMA-3

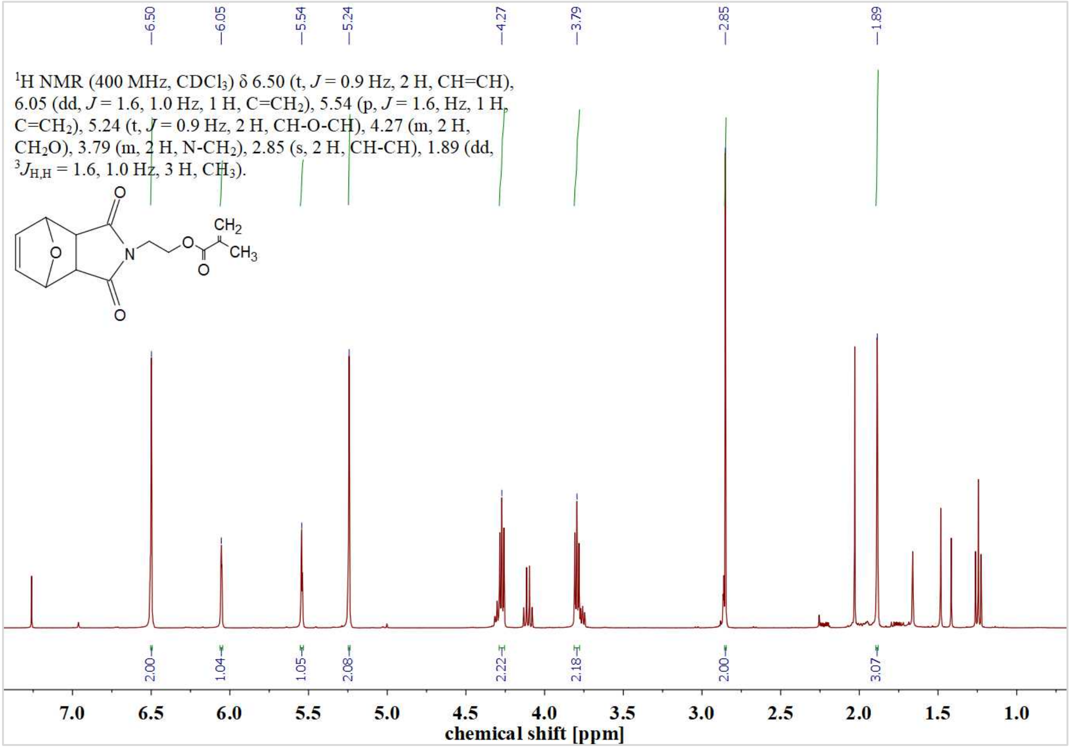

Figure S5. ^{1}H-NMR spectrum of the compound **3** of the furan protected maleimide methacrylate monomer in $CDCl_3$.

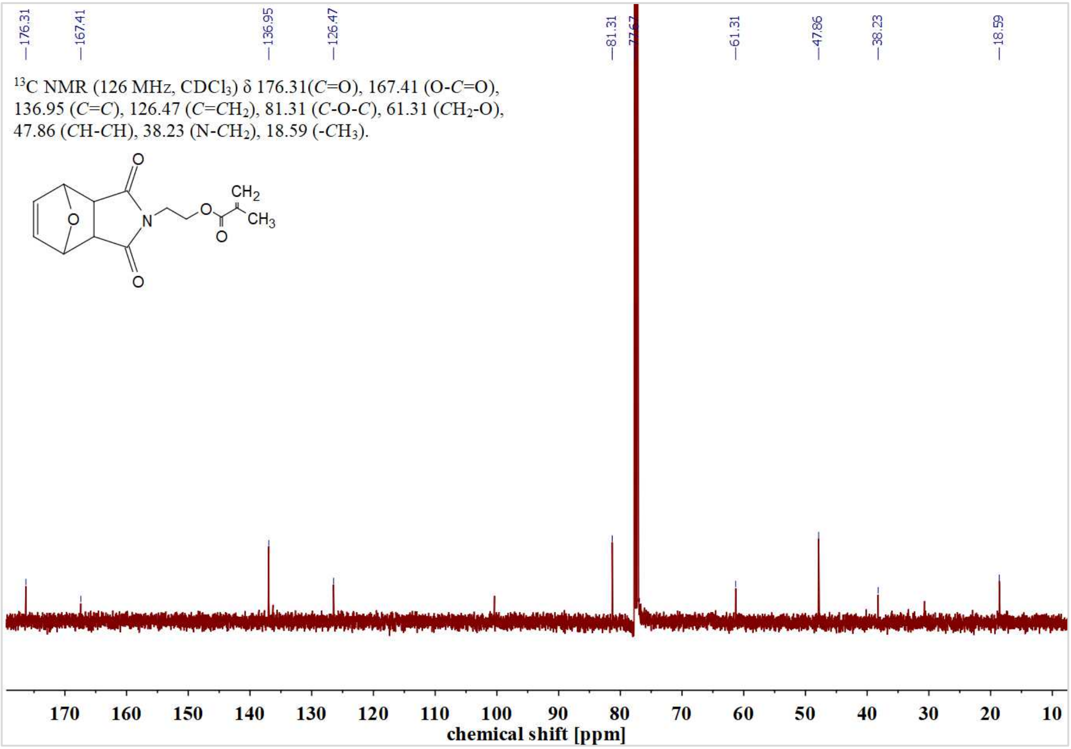

Figure S6. ^{13}C-NMR spectrum of the compound **3** of the furan protected maleimide methacrylate monomer in $CDCl_3$.

2. Polymer synthesis

2.1 ATRP Synthesis of polymers bearing furfuryl groups

^{1}H-NMR spectra, SEC measurements, DSC measurements and determination of the degree of functionalization of the polymers bearing furfuryl groups.

PFMA[5]

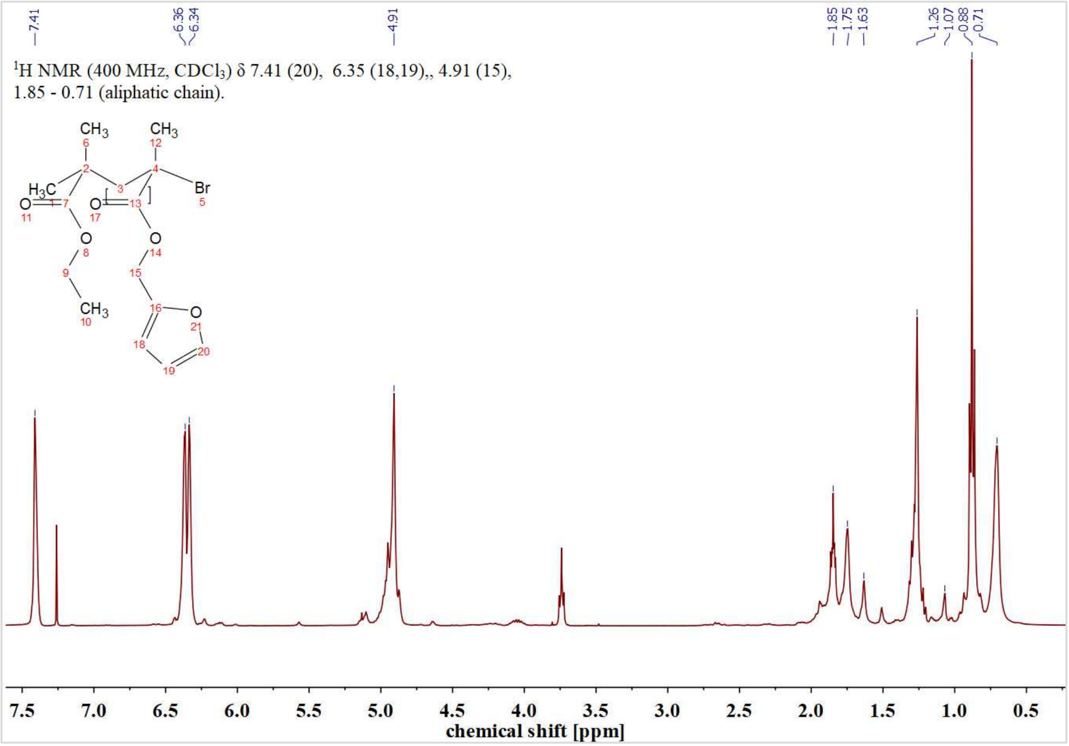

Figure S7. ^{1}H-NMR spectrum of the homopolymer PFMA[5] in $CDCl_3$.

P(MMA₈₉-co-FMA₁₁)[24]

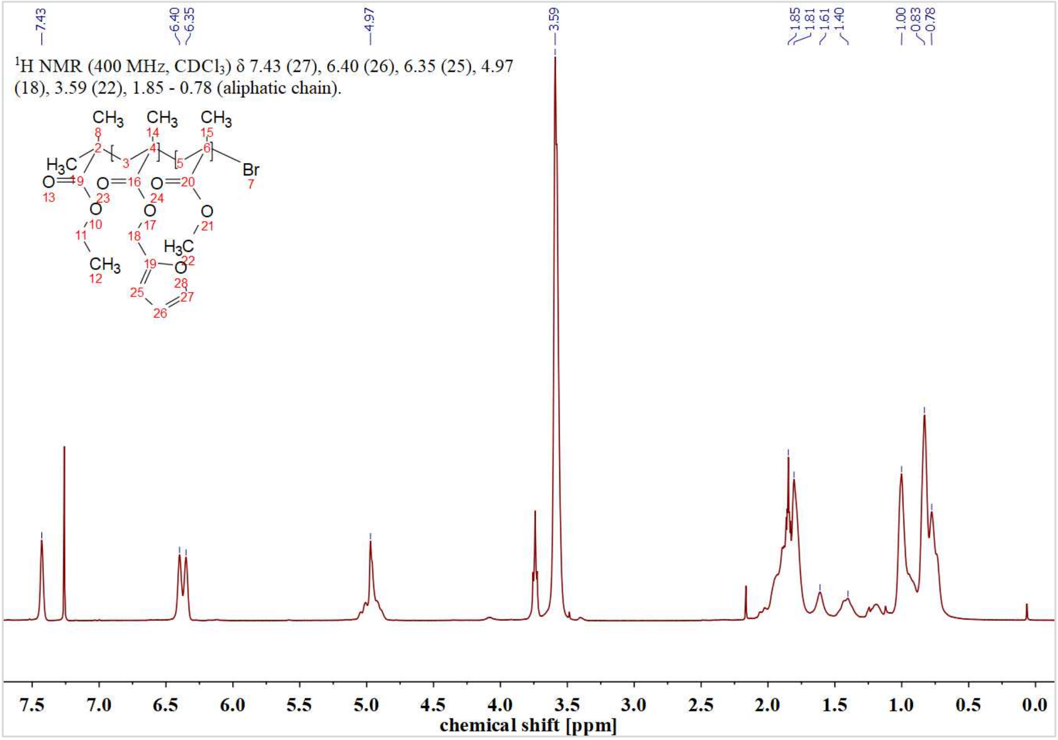

Figure S8. ^{1}H-NMR spectrum of the copolymer P(MMA$_{89}$-*co*-FMA$_{11}$)[24] in $CDCl_3$.

P(BA₆₀-co-FMA₄₀)[10]

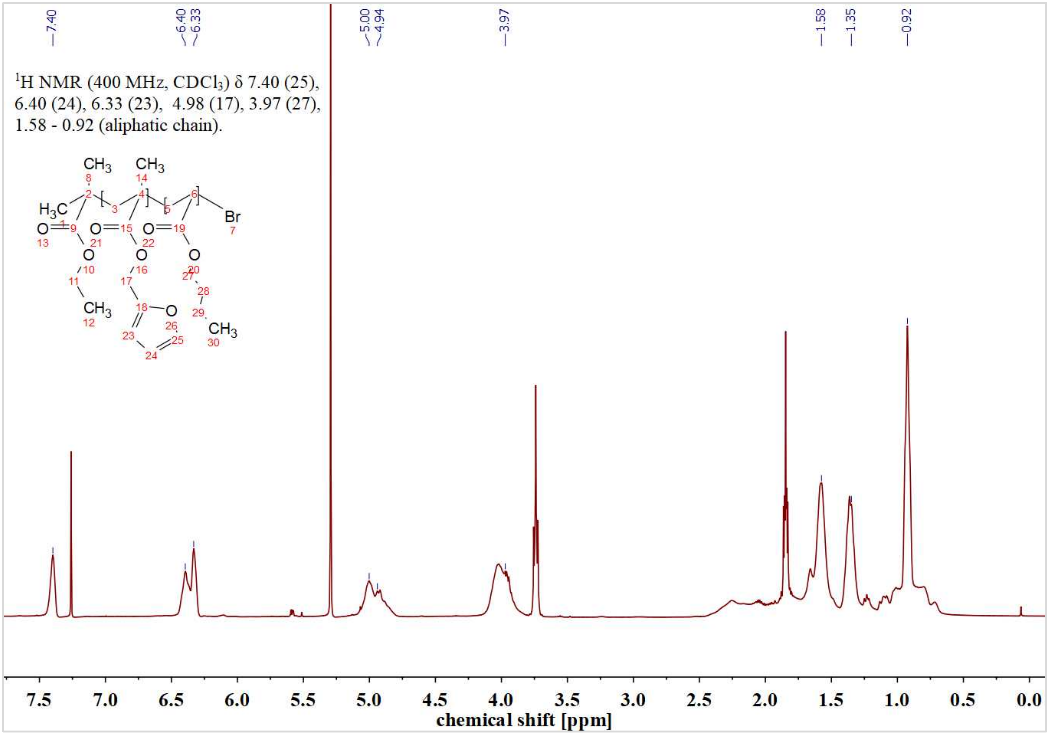

Figure S9. ^{1}H-NMR spectrum of the copolymer P(BA$_{60}$-co-FMA$_{40}$)[10] in $CDCl_3$.

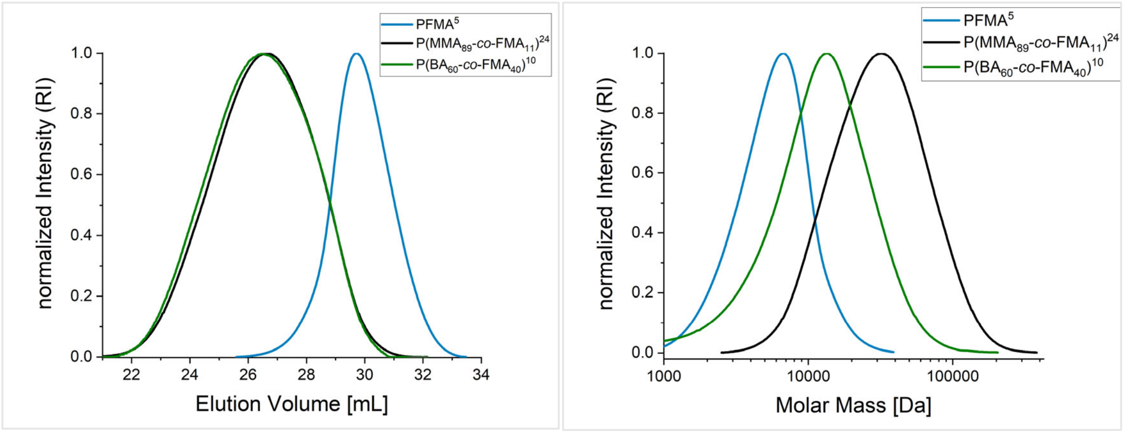

(a) (b)

Figure S10. SEC traces of the furfuryl bearing polymers. a) Elution volume and b) molar mass of PFMA[5] (blue), P(MMA_{89}-*co*-FMA_{11})[24] (black) and P(BA_{60}-co-FMA_{40})[10] (green).

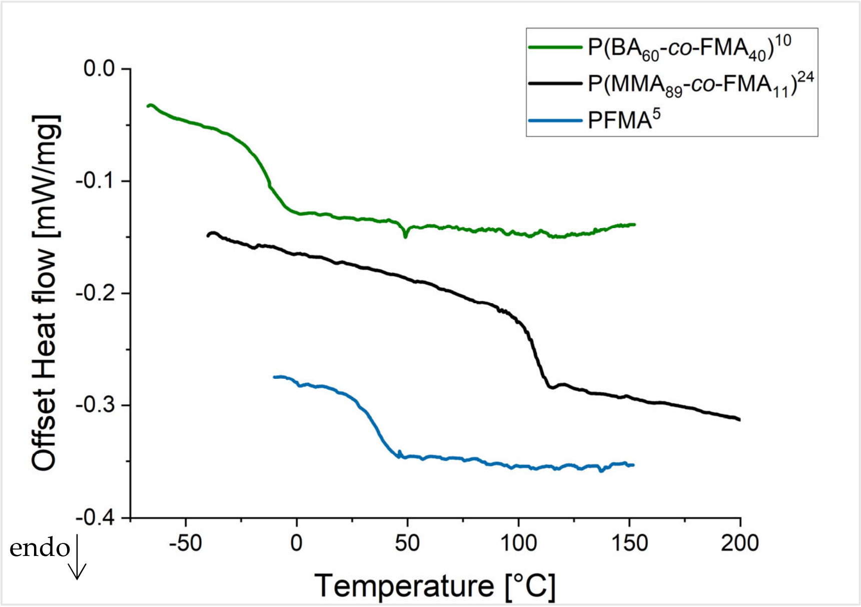

Figure S11. DSC traces of the furfuryl bearing polymers PFMA[5] (blue), P(MMA_{89}-*co*-FMA_{11})[24] (black) and P(BA_{60}-co-FMA_{40})[10] (green) for the determination of the glass transition temperature.

Table S1. Glass transition temperatures (T_g) determined by onset and in the middle of the endothermic transition of the three different furfuryl bearing polymers.

Polymer	T_g onset [°C]	T_g middle [°C]
PFMA[5]	18	39
P(MMA$_{89}$-*co*-FMA$_{11}$)[24]	93	109
P(BA$_{60}$-*co*-FMA$_{40}$)[10]	-34	-17

Calculation of the degree of functionalization (D_f) of the furfuryl bearing polymers

The proportions of monomer units or the degree of functionalization were calculated from the integrals of the ^{1}H-NMR spectrum (Fig. S12). For the P(MMA$_{89}$-*co*-FMA$_{11}$)[24] (Fig. S12a) the integrals of the signals at 4.9 ppm, corresponding to the methylene group of the furfuryl methacrylate (-O-CH$_2$) and at 3.6 ppm, corresponding to the methylene group of the methyl methacrylate (-O-CH$_3$), were used. For the P(BA$_{60}$-*co*-FMA$_{40}$)[10] (Fig. S12b) the integrals of the signals at 4.9 ppm, corresponding to the methylene group of the furfuryl methacrylate (-O-CH$_2$) and at 4.0 ppm, corresponding to the methylene group next to the oxygen of the *n*-butyl acrylate (-O-CH$_2$), were used.

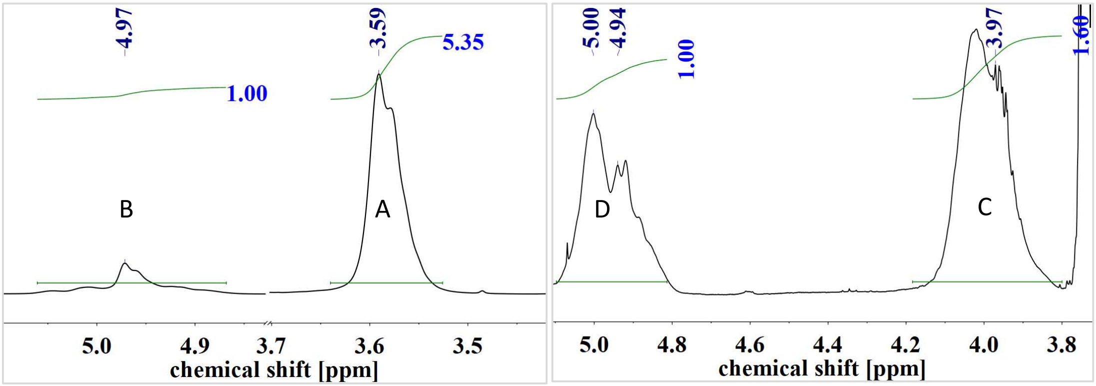

(a) (b)

Figure S12. Section of the ^{1}H-NMR spectrum of the copolymers P(MMA$_{89}$-*co*-FMA$_{11}$)[24] (a) and P(BA$_{60}$-*co*-FMA$_{40}$)[10] (b) in $CDCl_3$ for the determination of the degree of functionalization. Shown are the respective peaks for (a) 4.97 and 3.59 ppm and for (b) 4.97 and 3.97 ppm with their specific integral.

For the calculation of the degree of functionalization following equations (1 (MMA), 2 (BA)) were used.

$$D_f = \frac{2\,B}{2\,B + 3\,A} \quad (1)$$

$$D_f = \frac{2\,D}{2\,D + 3\,C} \quad (2)$$

In the equation D_f stands for the degree of functionalization and A – D for the integrals of the corresponding peaks from the ^{1}H-NMR spectra (Fig. S12).

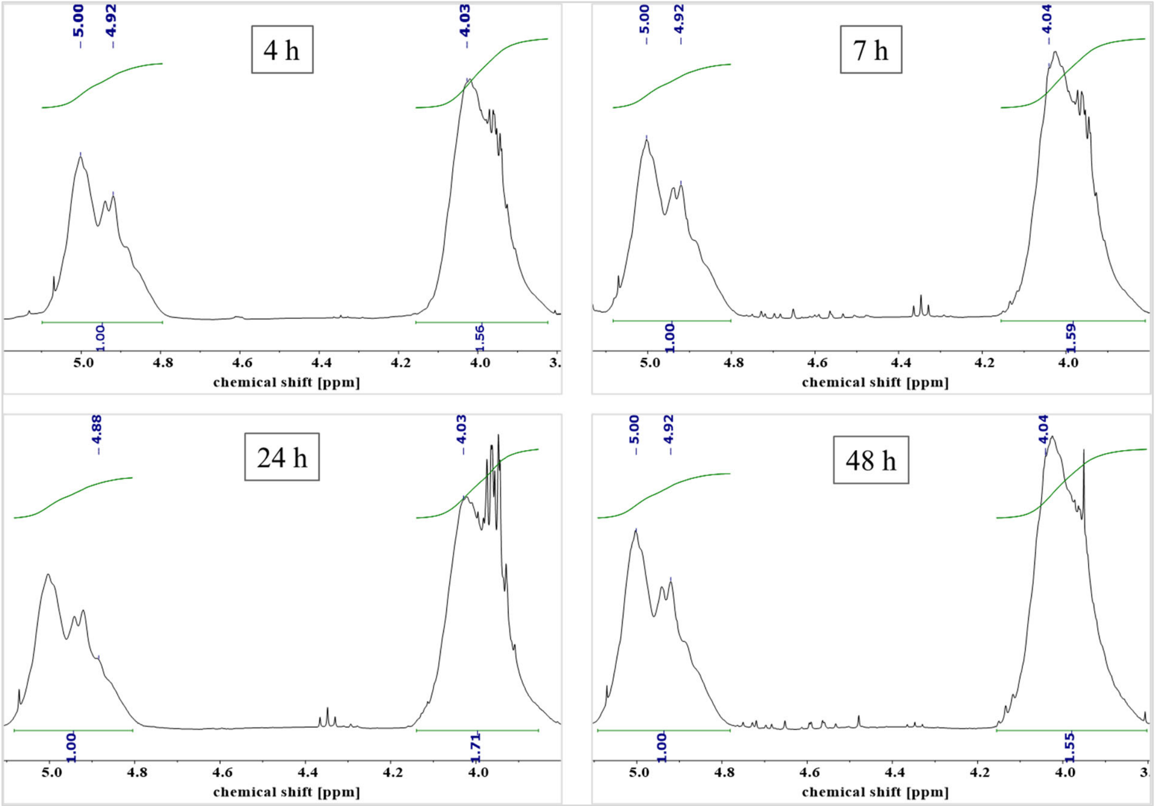

Figure S13. Sections of the ^{1}H-NMR spectra of the copolymer P(BA$_{60}$-*co*-FMA$_{40}$)[10] in $CDCl_3$. Shown are the respective peaks 4.97 and 3.97 ppm with their specific integral for the determination of the degree of functionalization over a period. The proportion of the two monomers used are the same over 48 h.

2.2 Free radical polymerization of copolymers bearing maleimide motifs

^{1}H-NMR spectra, SEC measurements, DSC measurements and determination of the degree of functionalization of the polymer bearing maleimide groups.

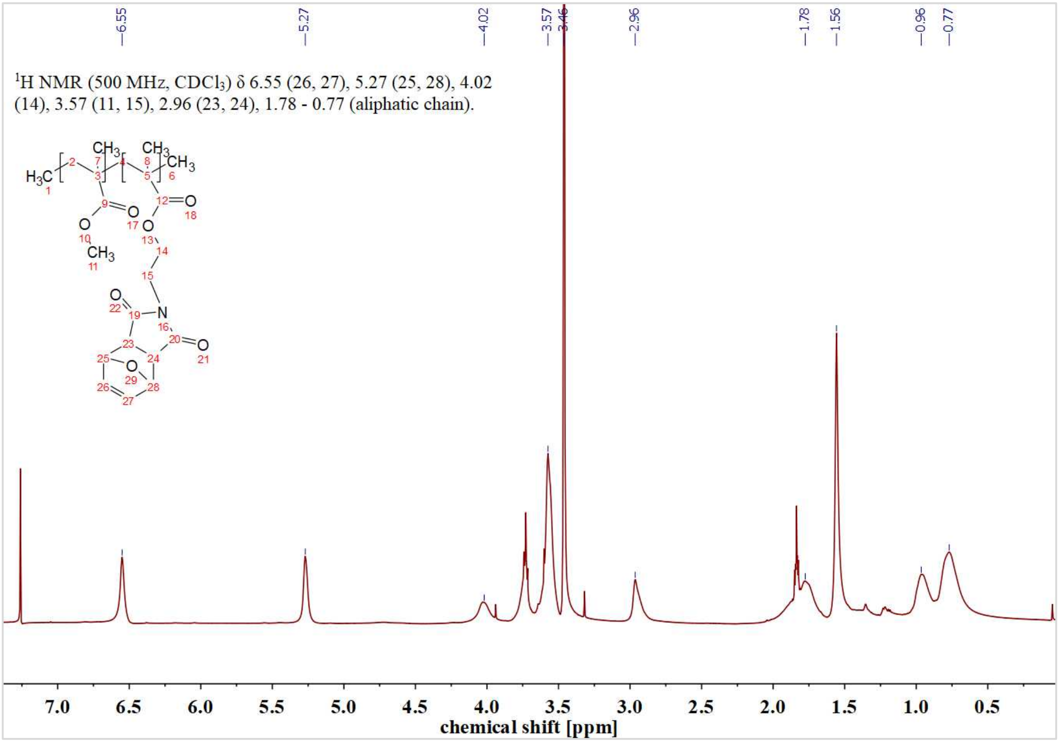

Figure S14. ^{1}H-NMR spectrum of the furan protected copolymer P(MMA$_{74}$-*co*-MIMA$_{26}$)[9] in $CDCl_3$.

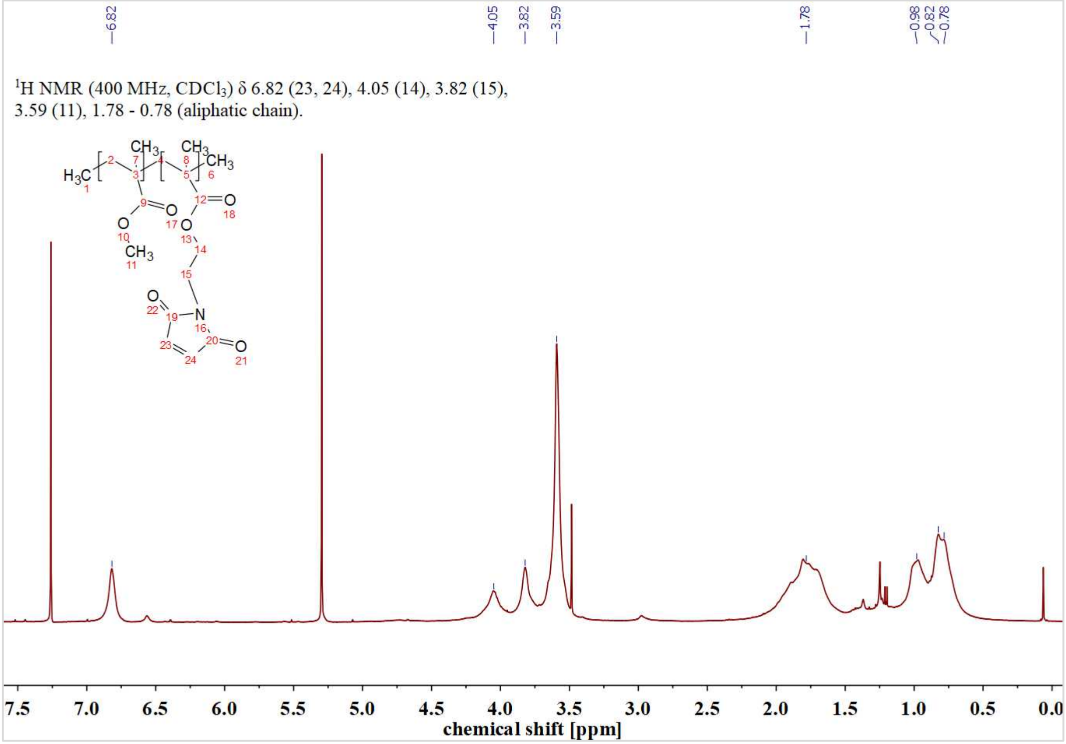

Figure S15. ^{1}H-NMR spectrum of the deprotected copolymer P(MMA$_{74}$-*co*-MIMA$_{26}$)[9] in $CDCl_3$.

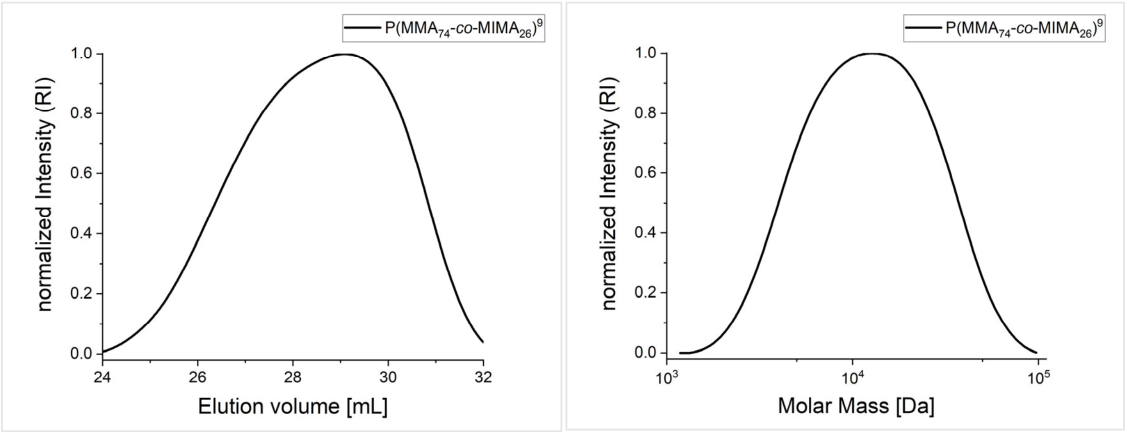

(a) (b)

Figure S16. SEC traces of the maleimide bearing copolymer. a) Elution volume and b) molar mass of P(MMA$_{74}$-*co*-MIMA$_{26}$)^{9}.

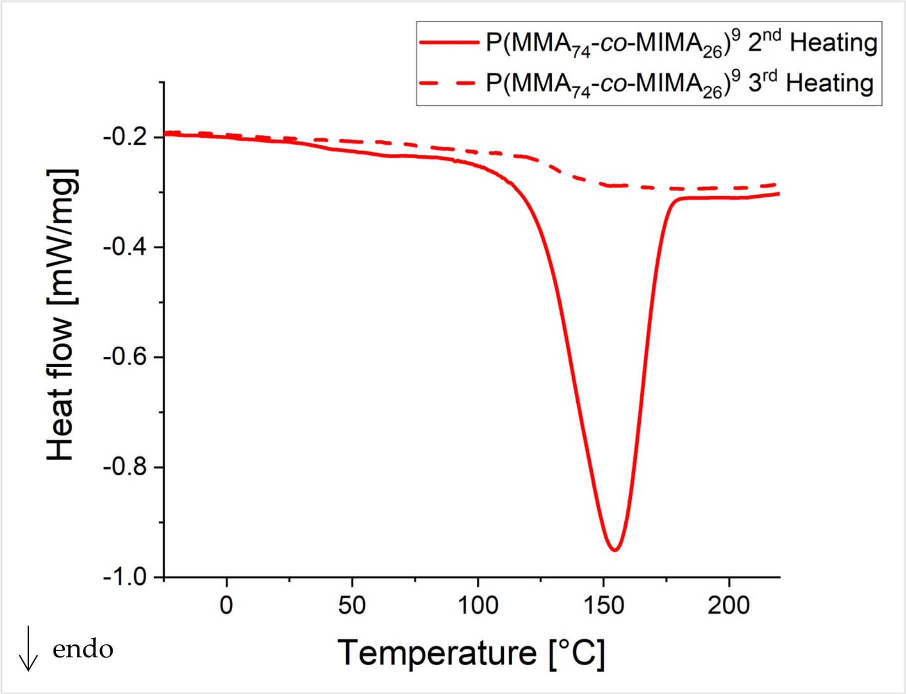

Figure S17. DSC traces of the maleimide bearing copolymer P(MMA$_{74}$-*co*-MIMA$_{26}$)^{9} for the determination of the glass transition temperature.

Table S2. Glass transition temperature (T_g) determined by onset and in the middle of the endothermic transition of the maleimide bearing copolymer.

Polymer	Tg onset [°C]	Tg middle [°C]
P(MMA$_{74}$-*co*-MIMA$_{26}$)[9]	120	135

Calculation of the degree of functionalization of the maleimide bearing polymers

To determine the degree of functionalization, the ratio of the peak area at a chemical shift of 6.51 ppm and 3.57 ppm was used (Fig. S18). The signal of 6.51 ppm can be assigned to the double bond of the oxanorbornene ring belonging to the monomer MIMA. The signal at 3.57 ppm corresponds to the methylene group of the methyl methacrylate monomer.

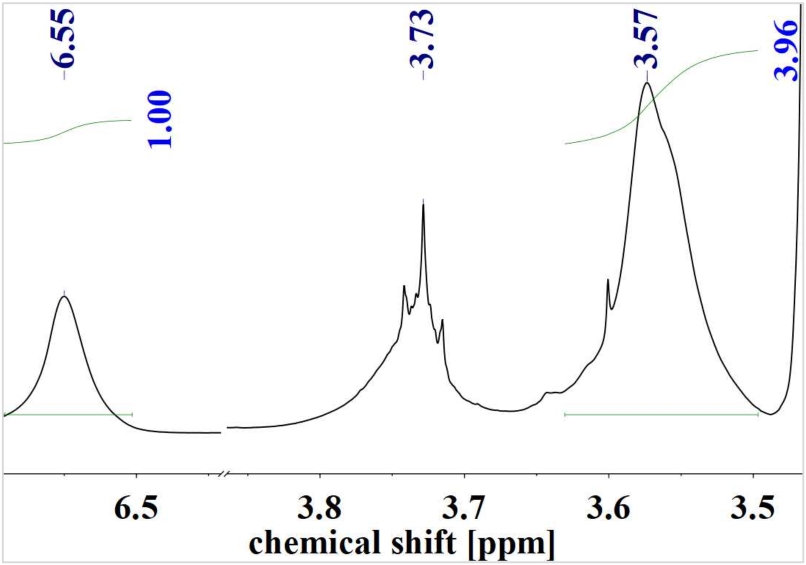

Figure S18. Section of the ^{1}H-NMR spectrum of the statistical Copolymer P(MMA$_{74}$-*co*-MIMA$_{26}$)[9] in $CDCl_3$ for the determination of the degree of functionalization. Shown are the respective peaks (6.51 and 3.57 ppm) with their specific integral. The peak at 3.76 ppm belongs to THF.

2.3 Deprotection of the maleimide bearing copolymer

When investigating the glass transition temperature by DSC, no glass transition can be observed in the second heating curve (Fig. S18; -50 °C – 225°C), at this is superimposed by an endothermic signal starting at 100 °C. This endothermic signal (22,.2 J/g) is the point at which furan as a protective group is split off by a retro-Diels-Alder reaction and leaves the system. This is due to the low boiling point of the furan (30°C). A glass transition can be seen in the subsequent cooling and heating curves. This starts at approx. 120 °C and shows an average glass transition temperature of 135 °C (Fig. S19).

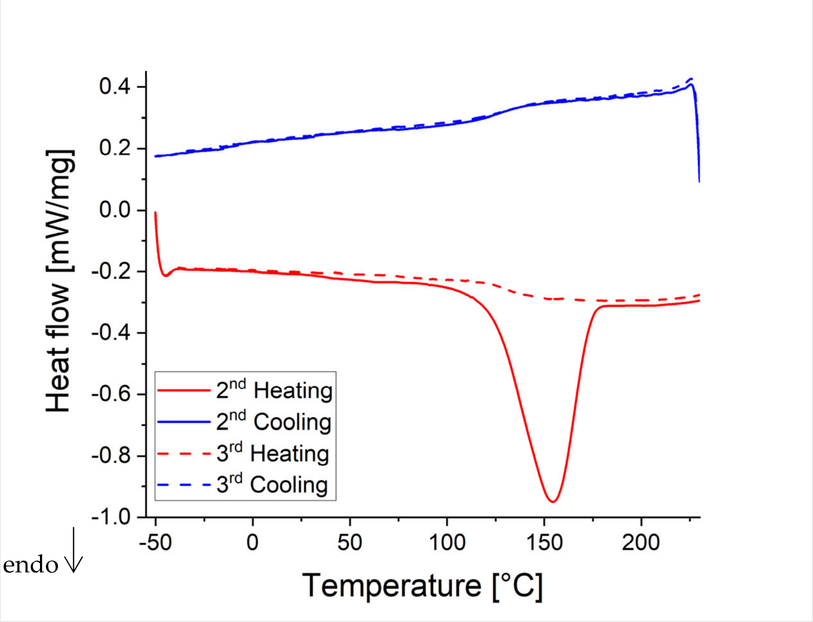

Figure S19. 2nd and 3rd heating (red) and cooling (blue) curve of the statistical copolymer P(MMA_{74}-*co*-$MIMA_{26}$)[9] measured with DSC for the determination of the glass transition temperature.

As shown by the DSC measurement the polymer is deprotected by using heat. The deprotection of the P(MMA_{74}-*co*-$MIMA_{26}$)[9] is needed for the subsequent crosslinking of the copolymer via the Diels-Alder reaction. The deprotected copolymer was analyzed by ^{1}H-NMR spectroscopy and FTIR spectroscopy (Fig. S20 & S21).

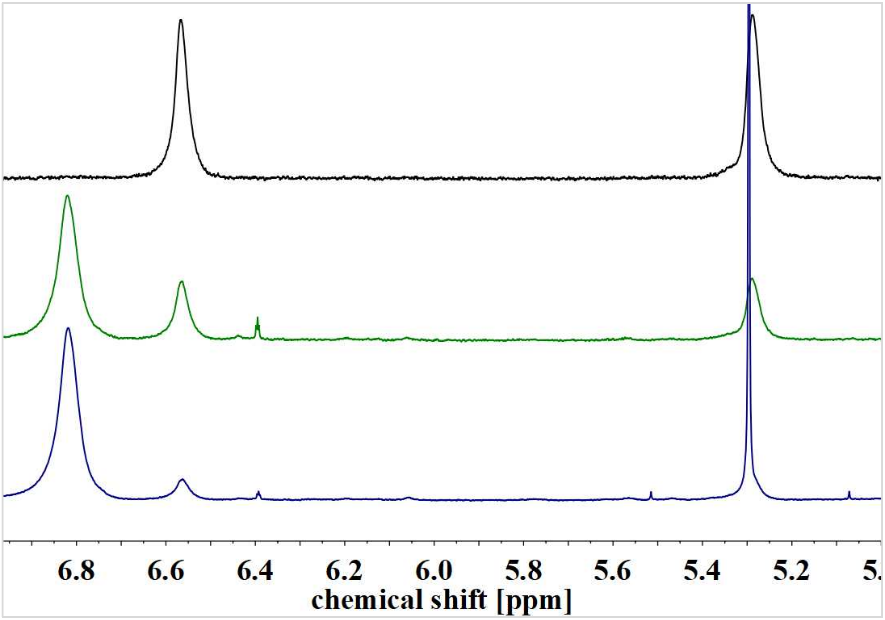

Figure S20. Sections of the ^{1}H-NMR spectra of the statistical Copolymer P(MMA$_{74}$-*co*-MIMA$_{26}$)[9] protected with furan (black) and after deprotection (green, 3 h at 125 °C and blue 6 h at 135 °C) in $CDCl_3$ for the determination of the conversion of the deprotection of the copolymer. Shown are the respective peaks 6.57 and 5.29 ppm for the protected copolymer and the respective peaks for the deprotected copolymer at 6.82 ppm. The peak at 5.30 ppm belongs to DCM.

In the ^{1}H-NMR spectrum, the cyclo-reversion is shown by the formation of a new signal at 6.82 ppm, which is attributed to the double bond of the maleimide ring. Furthermore, the signals at 6.57 ppm belonging to the double bond of the oxanorbornene and at 5.29 ppm belonging to the bridge heads of the oxanorbornene are disappearing. The comparison of the individual spectra shows that the deprotection was not completed at 125 °C for 3 h. With the help of the integral ratio of the signals at 6.57 ppm and 6.82 ppm the conversion of the deprotection could be determined. The deprotection at a temperature of 125 °C shows a conversion of 80%. To obtain a complete deprotection of the copolymer, the temperature was increased (135 °C) and the reaction time doubled. In this case the conversion of the deprotection was 93%.

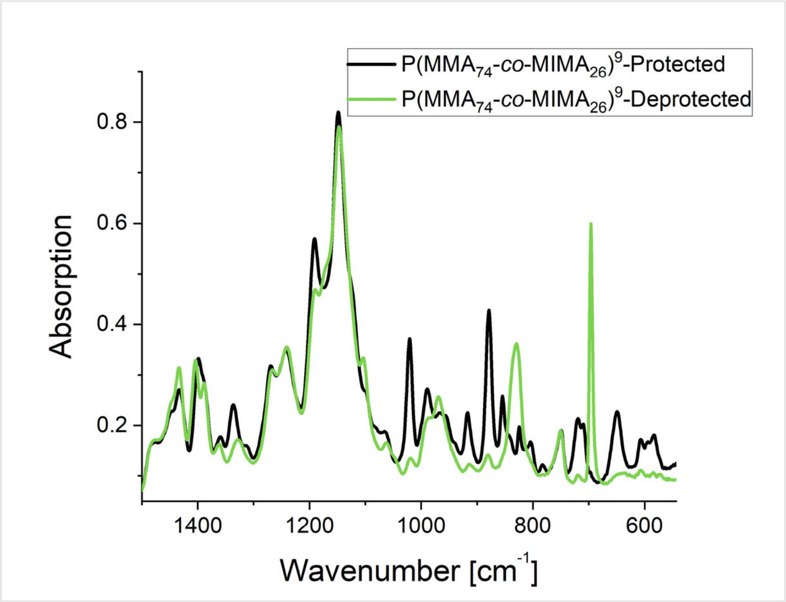

Figure S21. Section of the FTIR spectra of the statistical copolymer P(MMA$_{74}$-*co*-MIMA$_{26}$)^{9} protected (black) and deprotected (green).

3. Crosslinking of furfuryl bearing polymers

3.1 Visual observation of gelation

Crosslinked P(MMA$_{89}$-co-FMA$_{11}$)^{24}

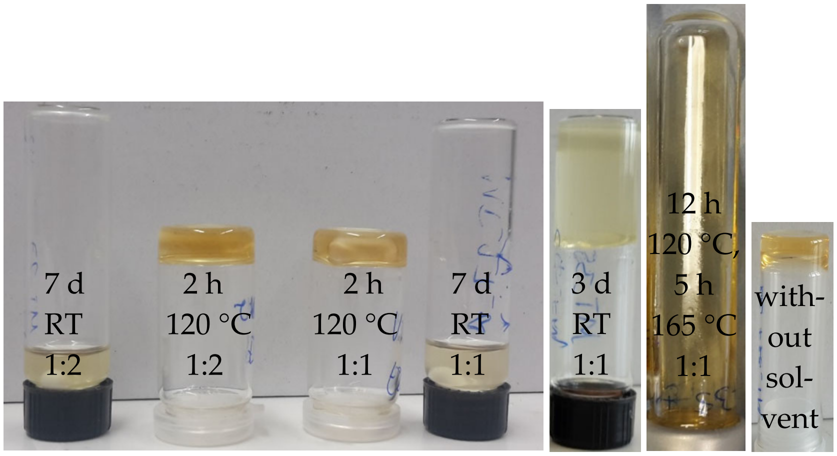

(a) (b)

Figure S22. Crosslinked P(MMA$_{89}$-*co*-FMA$_{11}$)^{24}. a) P(MMA$_{89}$-*co*-FMA$_{11}$)^{24} with BMI crosslinked at two different temperatures (room temperature (RT) and 120 °C) and two different molar ratios (1:2 and 1:1) in DCM. b) Crosslinked P(MMA$_{89}$-*co*-FMA$_{11}$)^{24} with P(MMA$_{74}$-*co*-MIMA$_{26}$)^{9} at two different temperatures (room temperature (RT) in DCM and 120 °C; 165 °C in anisole) and after solvent removal.

Crosslinked P(BA$_{60}$-co-FMA$_{40}$)^{10}

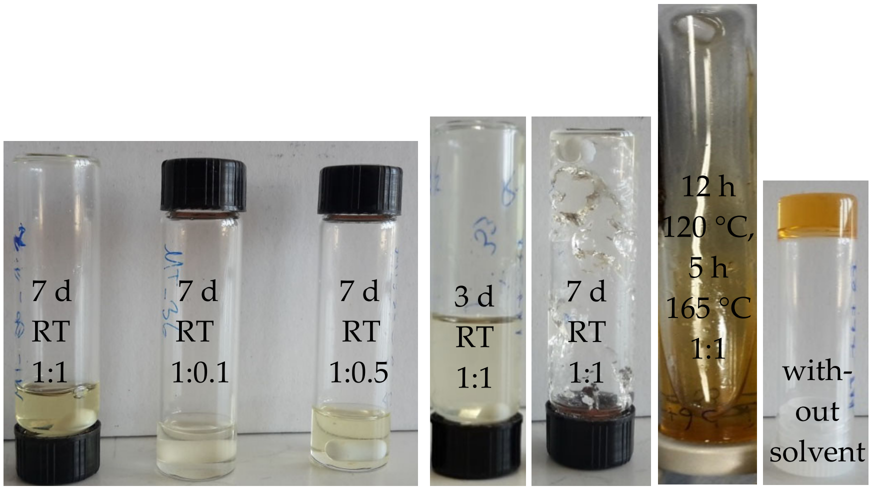

(a) (b)

Figure S23. Crosslinked P(BA$_{60}$-*co*-FMA$_{40}$)^{10}. a) P(BA$_{60}$-*co*-FMA$_{40}$)^{10} with BMI crosslinked with three different molar ratios (1:2; 1:1; 1:0.5) at the same temperature (room temperature (RT)) in DCM after one week. b) Crosslinked P(BA$_{60}$-*co*-FMA$_{40}$)^{10} with P(MMA$_{74}$-*co*-MIMA$_{26}$)^{9} at two different temperatures (room temperature (RT) in DCM and 120 °C; 165 °C in anisole) after solvent removal and the gelation process at room temperature after 3 days and 7 days.

3.2 Thermoreversibility of gelation

DSC and FTIR spectra of the crosslinked P(BA_{60}-*co*-FMA_{40})[10].

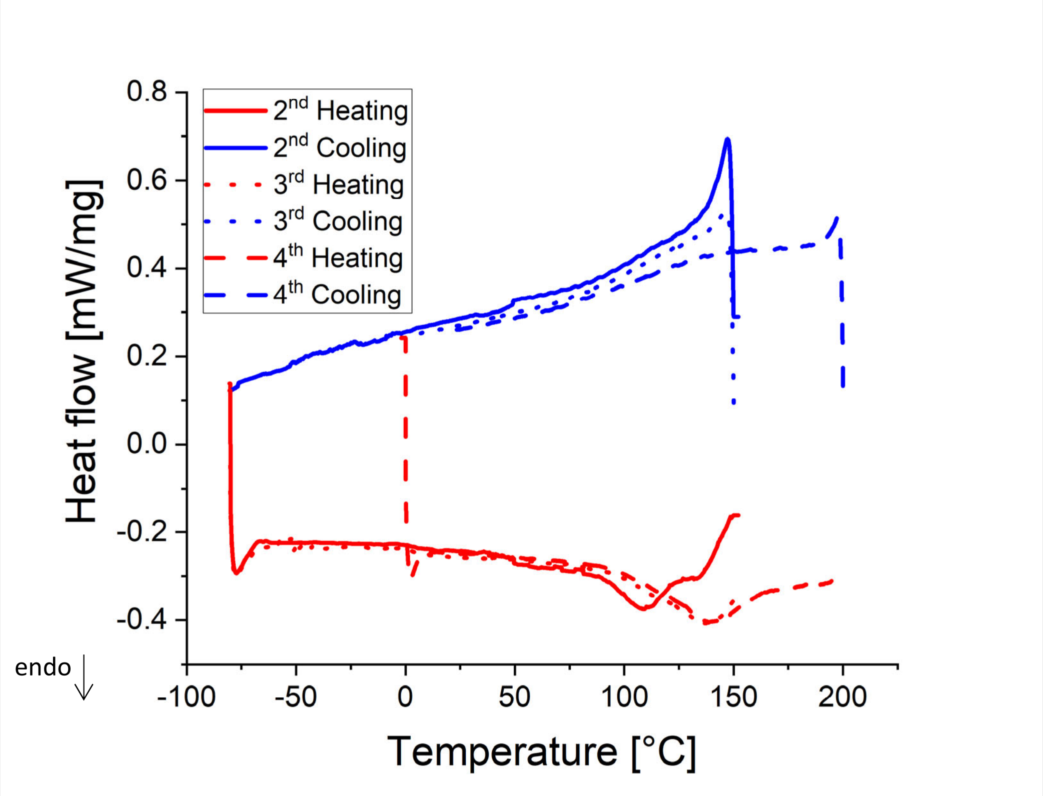

Figure S24. DSC heating (red) and cooling (blue) curves of the **c**rosslinked P(BA_{60}-*co*-FMA_{40})[10]. Crosslinked P(BA_{60}-*co*-FMA_{40})[10] with BMI in a motif ratio of 1:2. The endothermic peak indicates the retro-Diels Alder reaction at around 100 °C in all three heating curves.

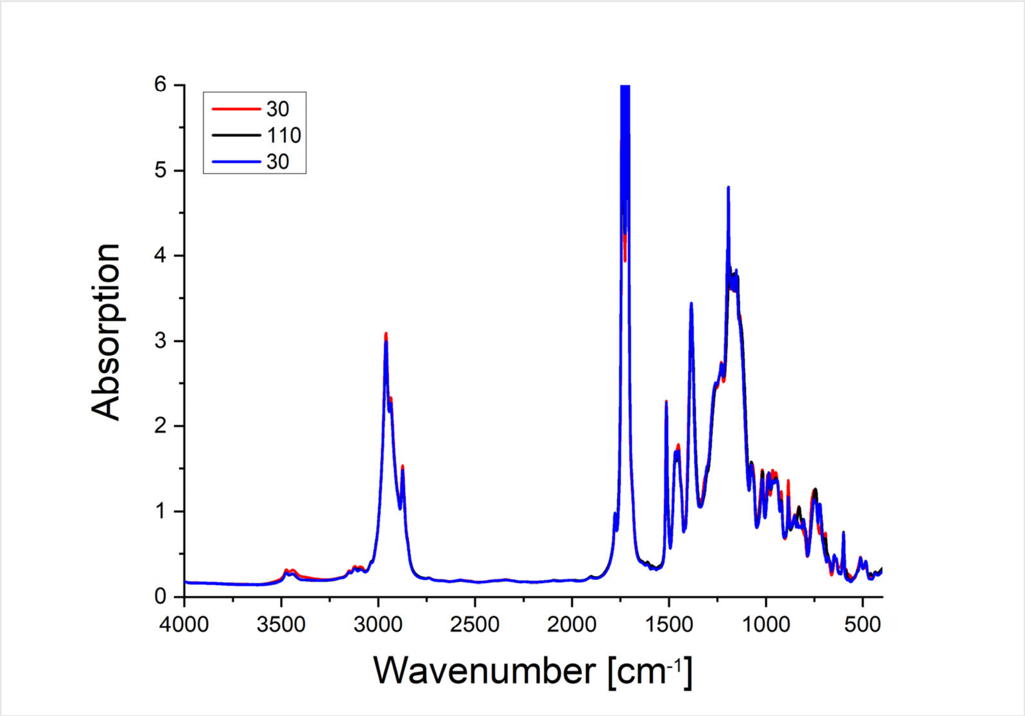

(a)

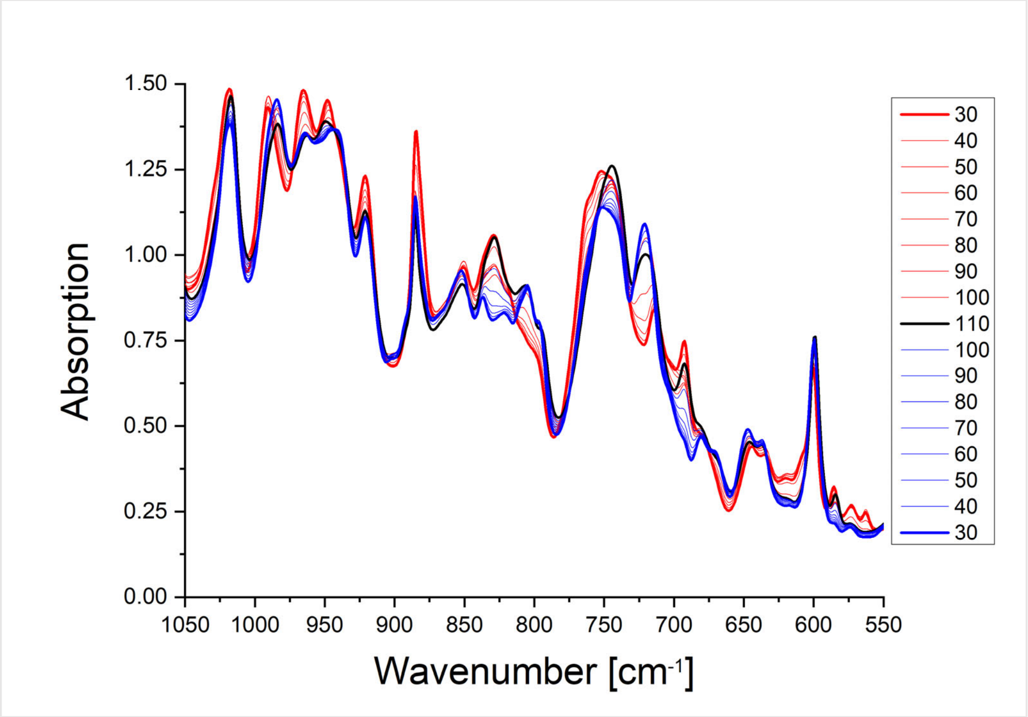

(b)

Figure S25. Temperature dependent FTIR spectra of the crosslinked P(BA$_{60}$-*co*-FMA$_{40}$)$_{10}$ with BMI at room temperature and with a motif ratio of 1:1. Spectra of the heating cycle (red), spectrum of the maximum temperature (black) and spectra of the cooling cycle (blue). The entire temperature cycle from 30-110-30 °C in the complete wavenumber range (a) and from 1050 to 550 cm^{-1} (b) is shown, as the ring deformation mode of the maleimide ring formed in the retro-Diels-Alder reaction can be observed.

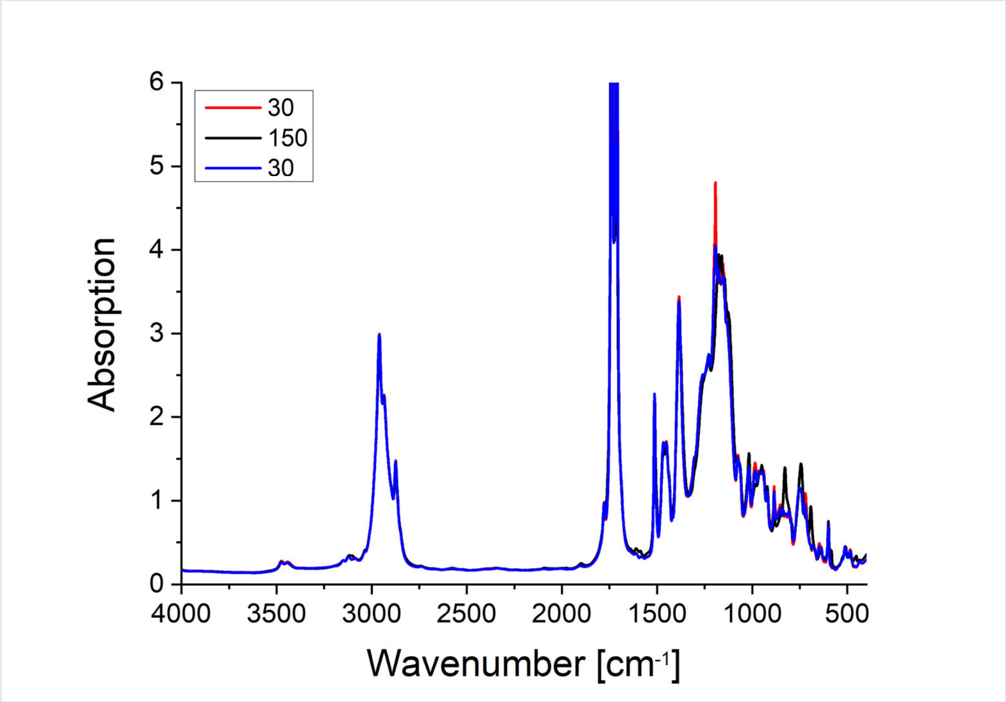

(a)

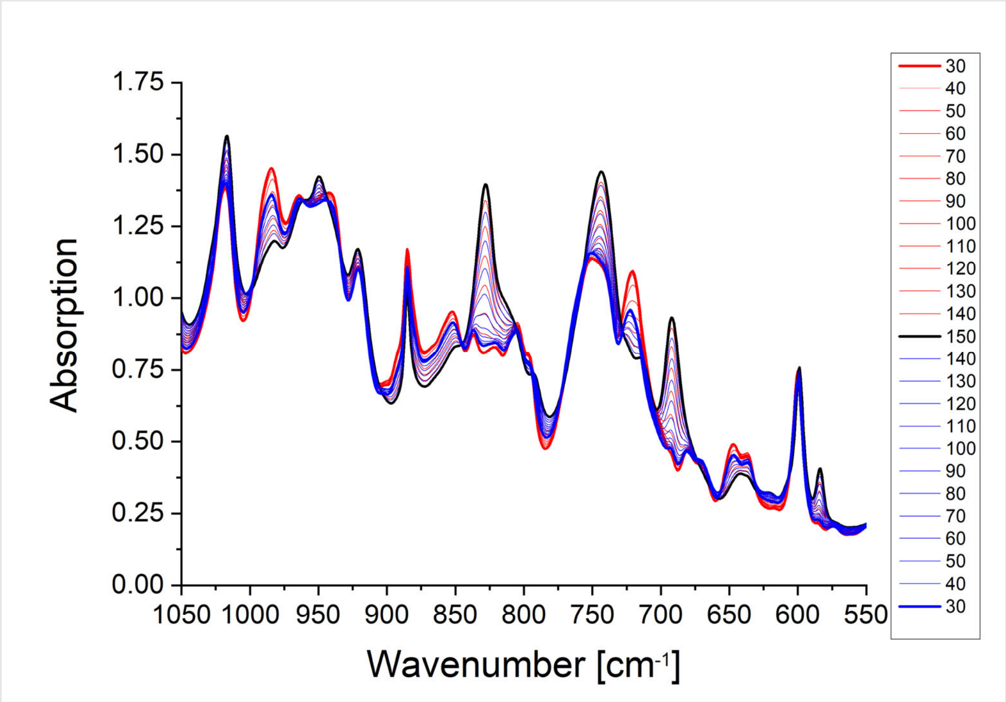

(b)

Figure S26. Temperature dependent FTIR spectra of the crosslinked P(BA_{60}-*co*-FMA_{40})^{10} with BMI at room temperature and with a motif ratio of 1:1. Spectra of the heating cycle (red), spectrum of the maximum temperature (black) and spectra of the cooling cycle (blue). The entire temperature cycle from 30-150-30 °C in the complete wavenumber range (a) and from 1050 to 550 cm^{-1} (b) is shown, as the ring deformation mode of the maleimide ring formed in the retro-Diels-Alder reaction can be easily observed.

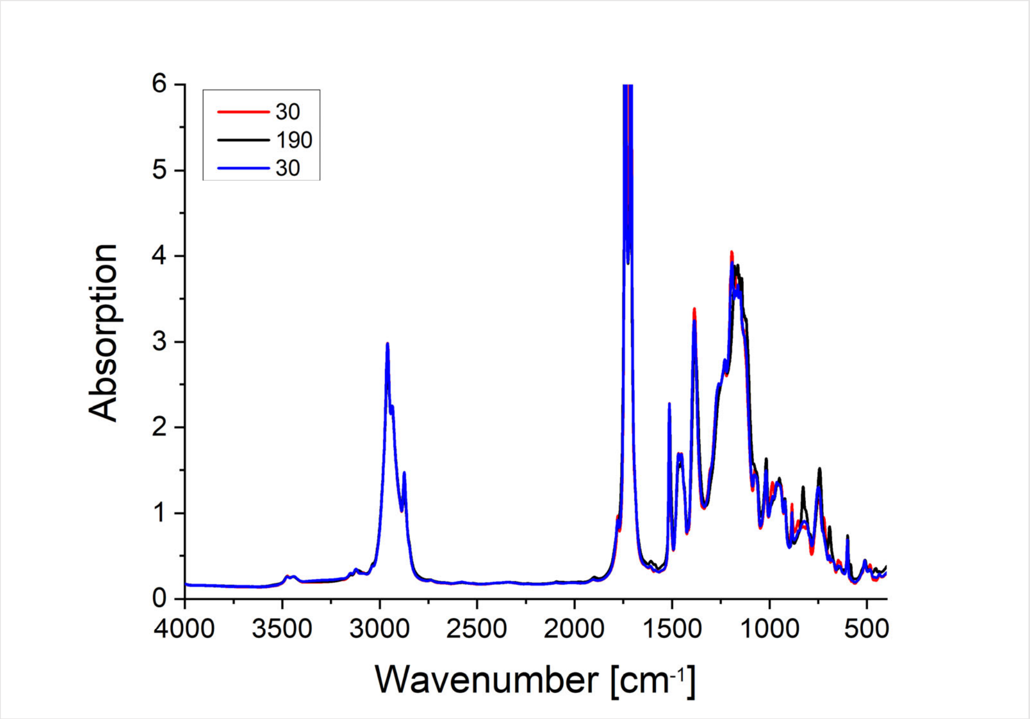

Figure S27. Temperature dependent FTIR spectra of the crosslinked P(BA$_{60}$-*co*-FMA$_{40}$)[10] with BMI at room temperature and with a motif ratio of 1:1. Spectra of the heating cycle (red), spectrum of the maximum temperature (black) and spectra of the cooling cycle (blue). The entire temperature cycle from 30-190-30 °C in the complete wavenumber range.

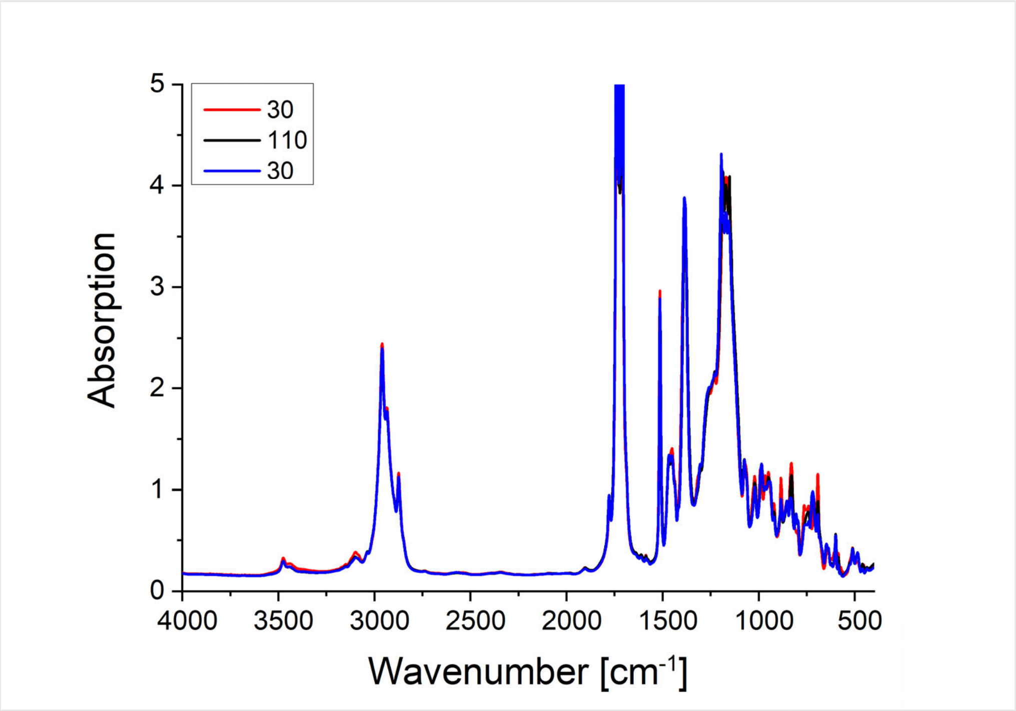

(a)

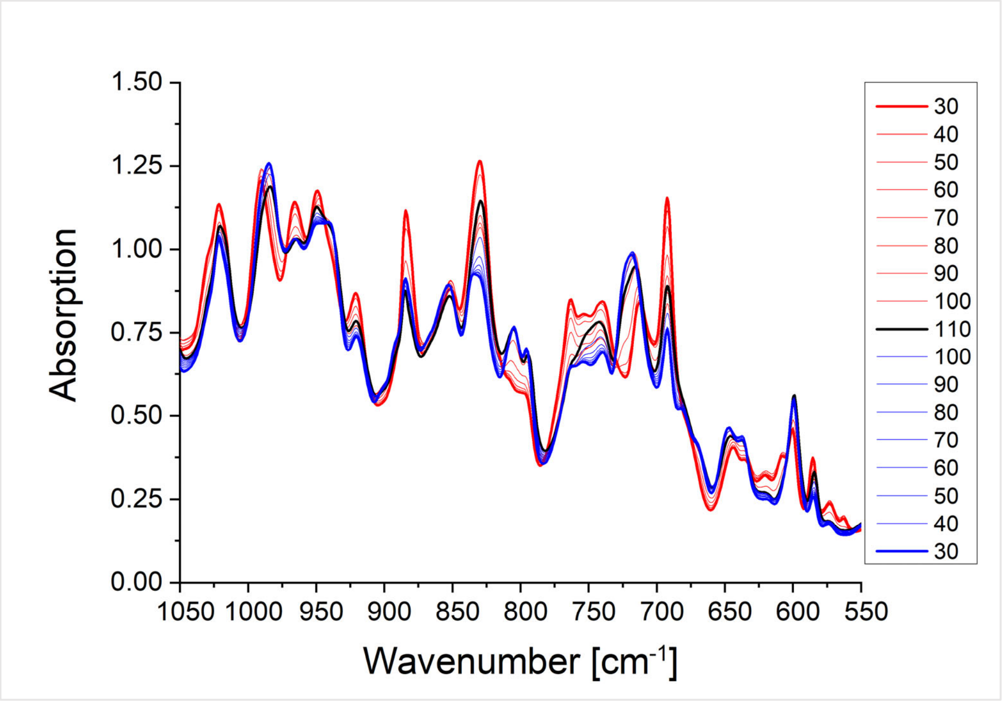

(b)

Figure S28. Temperature dependent FTIR spectra of the crosslinked P(BA_{60}-*co*-FMA_{40})[10] with BMI at room temperature and with a motif ratio of 1:2. Spectra of the heating cycle (red), spectrum of the maximum temperature (black) and spectra of the cooling cycle (blue). The entire temperature cycle from 30-110-30 °C in the complete wavenumber range (a) and from 1050 to 550 cm^{-1} (b) is shown, as the ring deformation mode of the maleimide ring formed in the retro-Diels-Alder reaction can be easily observed.

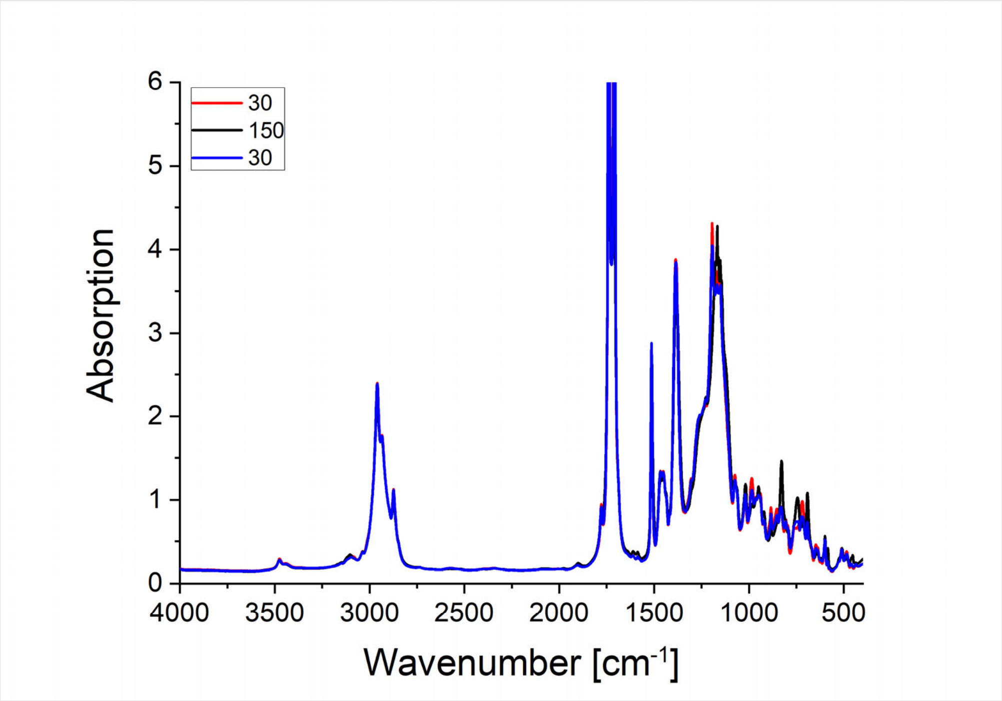

(a)

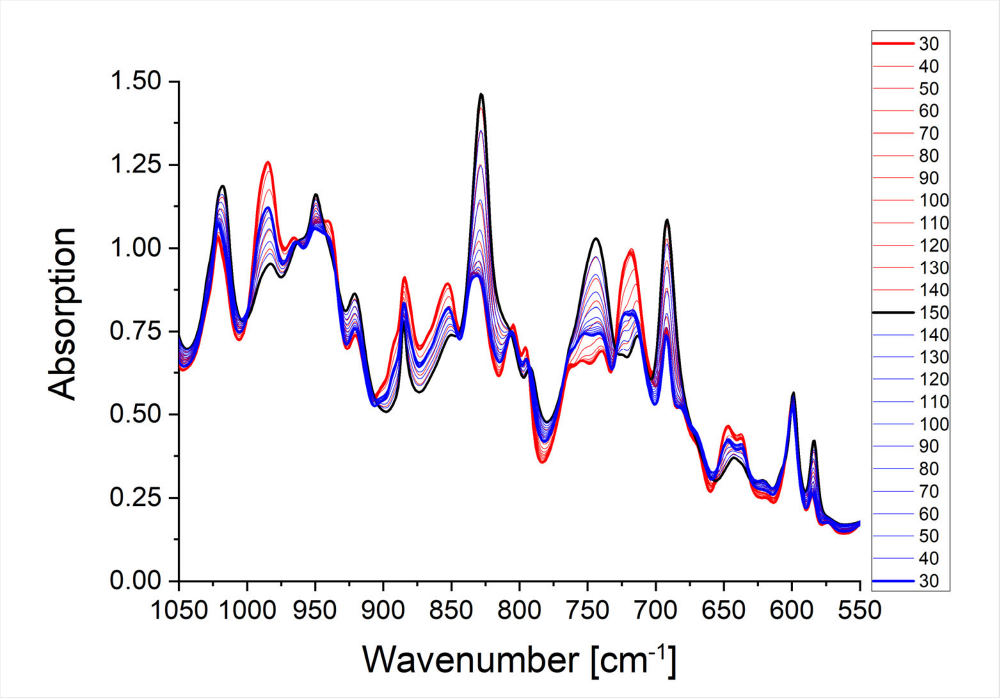

(b)

Figure S29. Temperature dependent FTIR spectra of the crosslinked $P(BA_{60}$-*co*-$FMA_{40})^{10}$ with BMI at room temperature and with a motif ratio of 1:2. Spectra of the heating cycle (red), spectrum of the maximum temperature (black) and spectra of the cooling cycle (blue). The entire temperature cycle from 30-150-30 °C in the complete wavenumber range (a) and from 1050 to 550 cm^{-1} (b) is shown, as the ring deformation mode of the maleimide ring formed in the retro-Diels-Alder reaction can be easily observed.

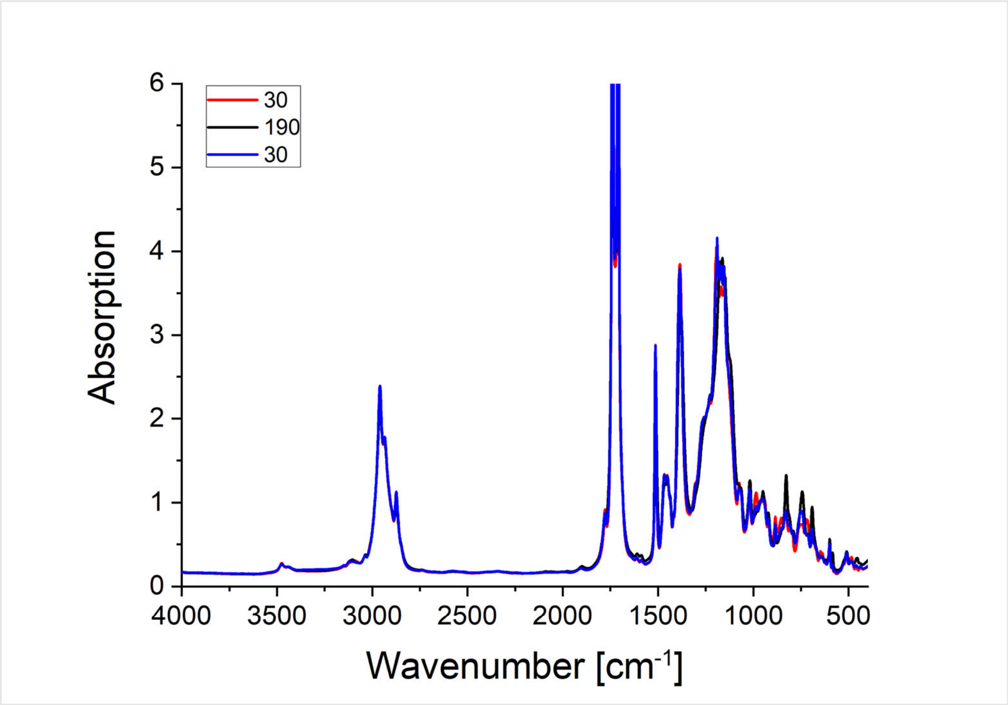

(a)

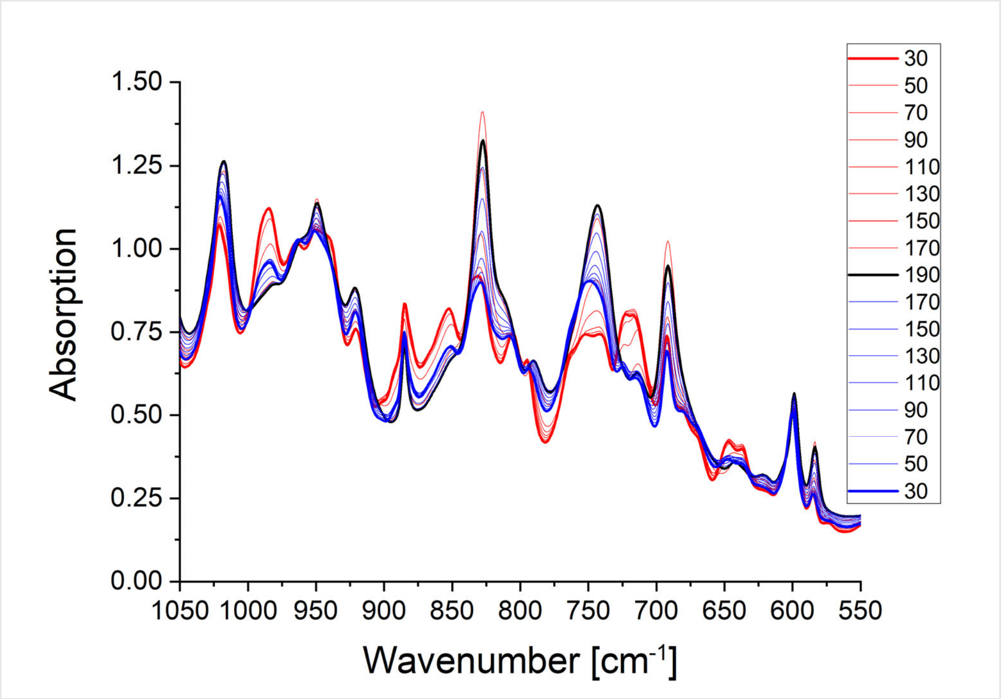

(b)

Figure S30. Temperature dependent FTIR spectra of the crosslinked P(BA_{60}-*co*-FMA_{40})[10] with BMI at room temperature and with a motif ratio of 1:2. Spectra of the heating cycle (red), spectrum of the maximum temperature (black) and spectra of the cooling cycle (blue). The entire temperature cycle from 30-190-30 °C in the complete wavenumber range (a) and from 1050 to 550 cm^{-1} (b) is shown, as the ring deformation mode of the maleimide ring formed in the retro-Diels-Alder reaction can be easily observed.

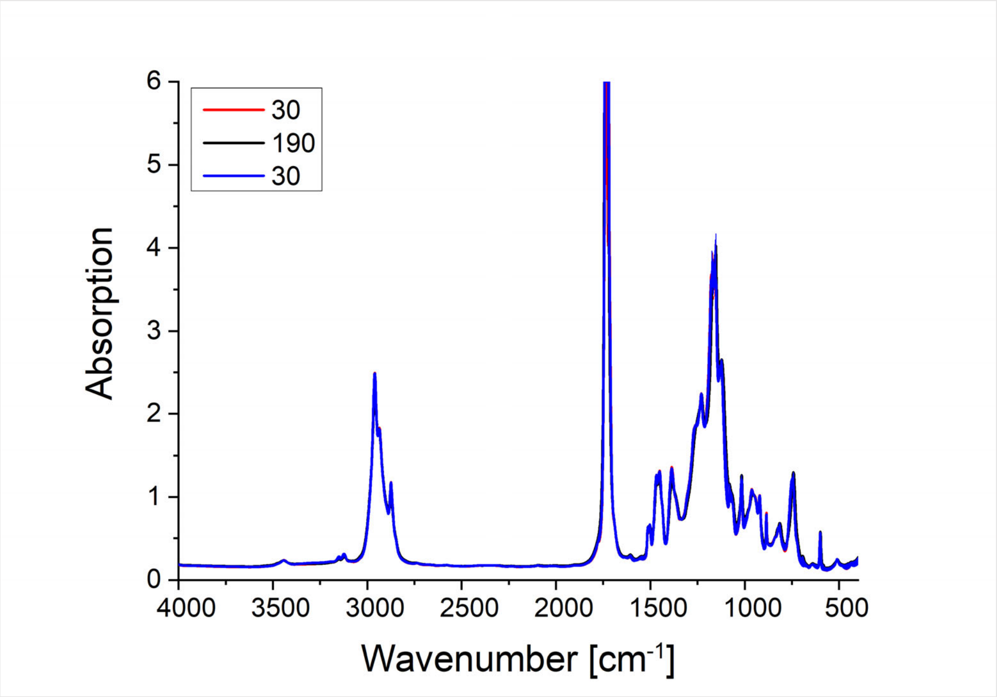

(a)

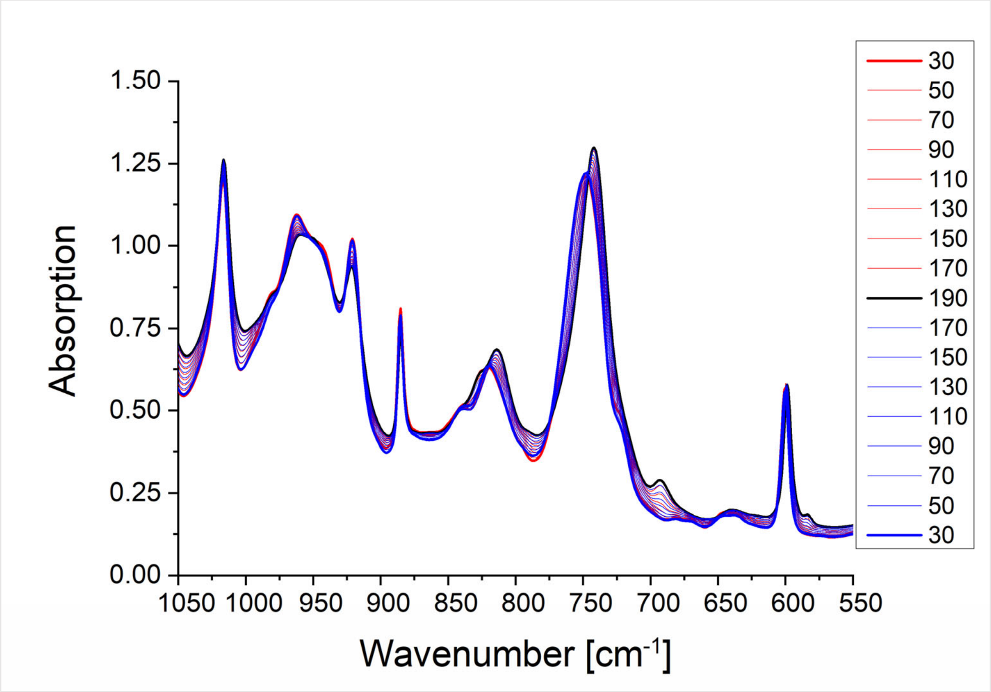

(b)

Figure S31. Temperature dependent FTIR spectra of the crosslinked P(BA_{60}-*co*-FMA_{40})[10] with BMI at room temperature and with a motif ratio of 1:0.5. Spectra of the heating cycle (red), spectrum of the maximum temperature (black) and spectra of the cooling cycle (blue). The entire temperature cycle from 30-190-30 °C in the complete wavenumber range (a) and from 1050 to 550 cm^{-1} (b) is shown, as the ring deformation mode of the maleimide ring formed in the retro-Diels-Alder reaction can be easily observed.

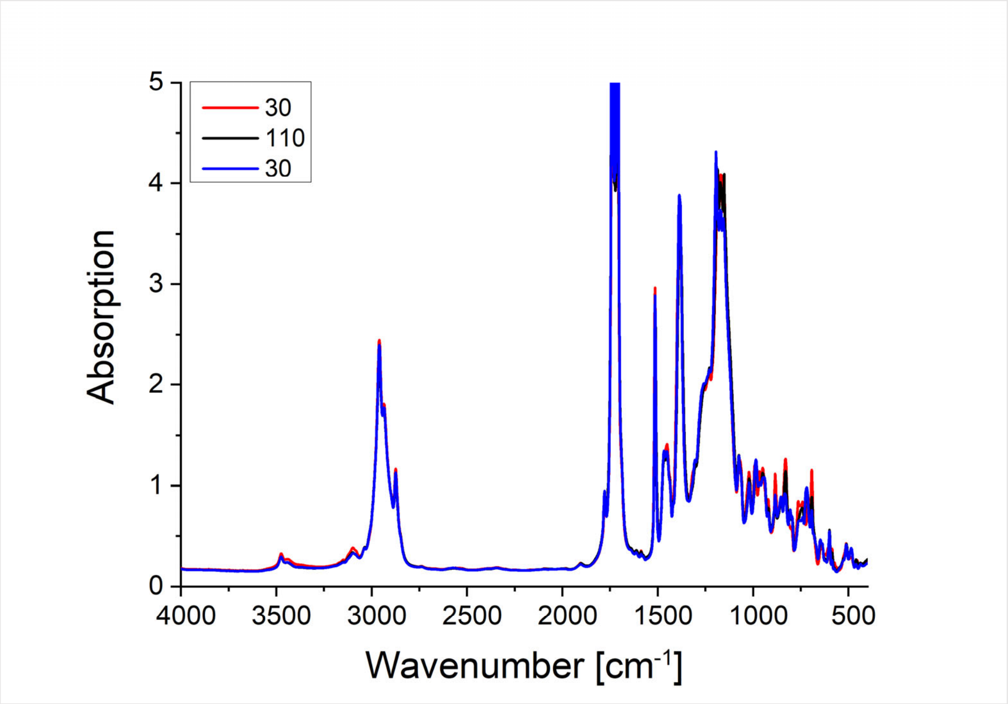

(a)

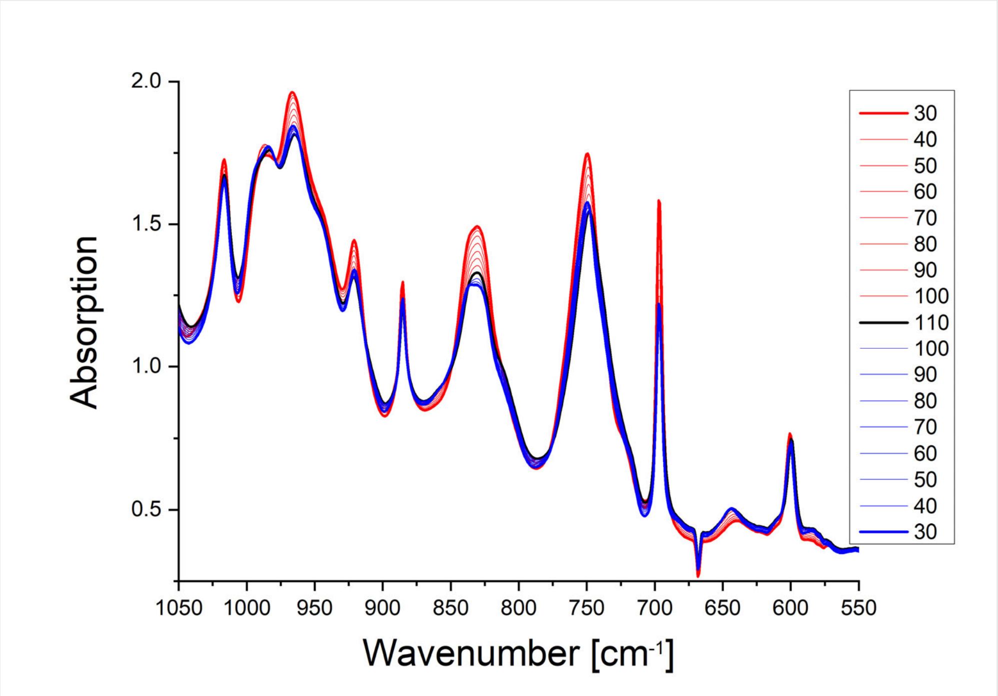

(b)

Figure S32. Temperature dependent FTIR spectra of the crosslinked P(BA_{60}-*co*-FMA_{40})[10] with P(MMA_{74}-*co*-$MIMA_{26}$)[9] at room temperature and with a motif ratio of 1:1. Spectra of the heating cycle (red), spectrum of the maximum temperature (black) and spectra of the cooling cycle (blue). The entire temperature cycle from 30-110-30 °C in the complete wavenumber range (a) and from 1050 to 550 cm^{-1} (b) is shown, as the ring deformation mode of the maleimide ring formed in the retro-Diels-Alder reaction can be easily observed.

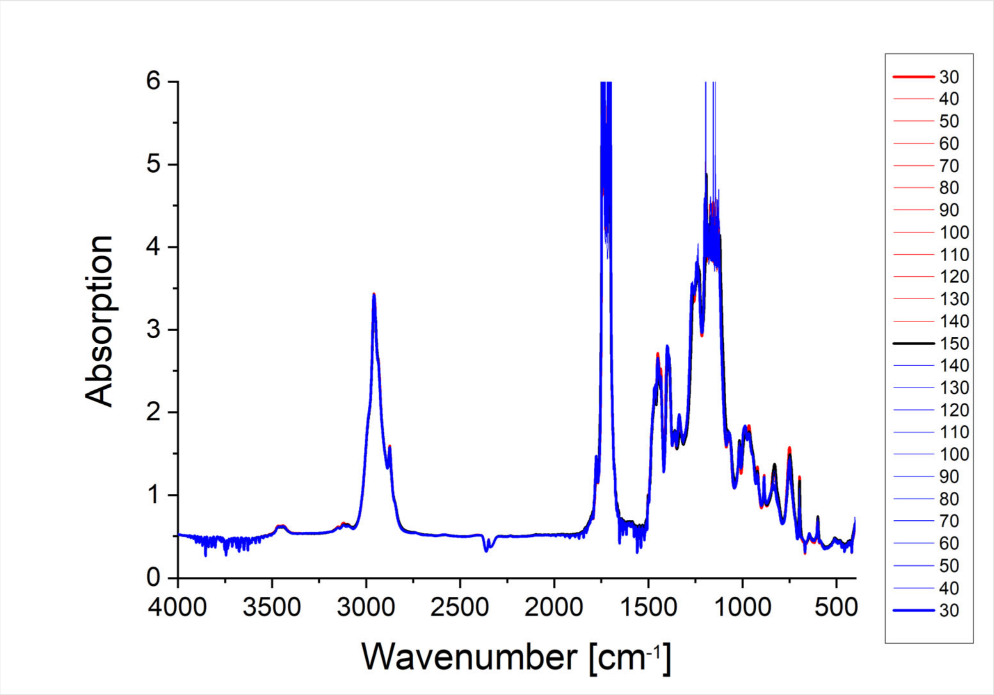

(a)

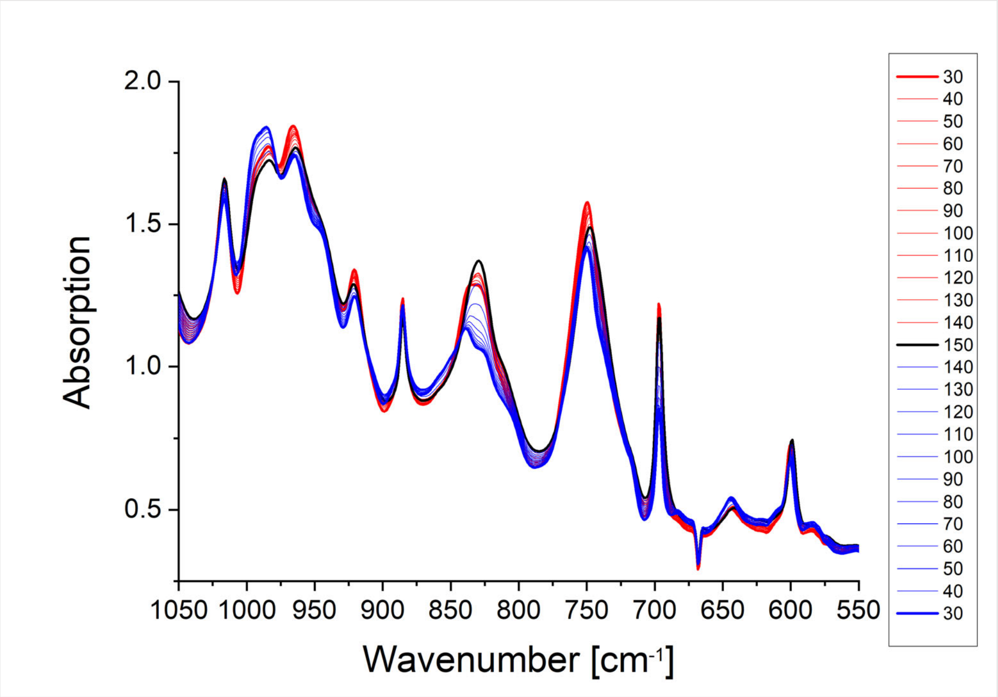

(b)

Figure S33. Temperature dependent FTIR spectra of the crosslinked P(BA_{60}-*co*-FMA_{40})[10] with P(MMA_{74}-*co*-$MIMA_{26}$)[9] at room temperature and with a motif ratio of 1:1. Spectra of the heating cycle (red), spectrum of the maximum temperature (black) and spectra of the cooling cycle (blue). The entire temperature cycle from 30-150-30 °C in the complete wavenumber range (a) and from 1050 to 550 cm^{-1} (b) is shown, as the ring deformation mode of the maleimide ring formed in the retro-Diels-Alder reaction can be easily observed.

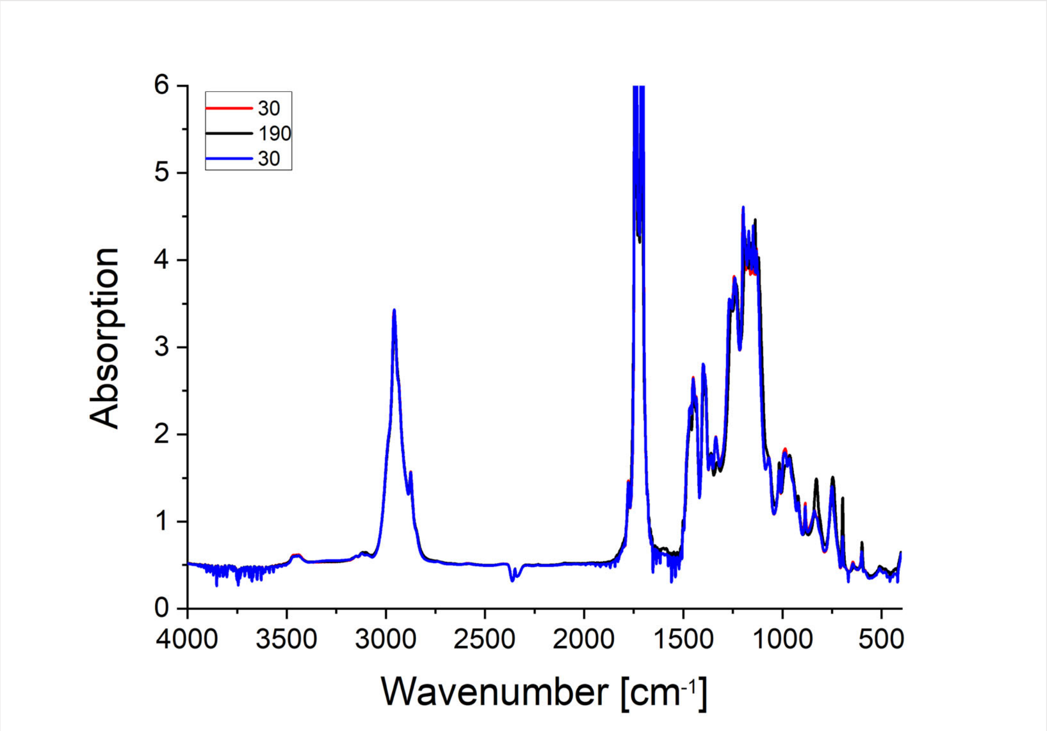

Figure S34. Temperature dependent FTIR spectra of the crosslinked P(BA_{60}-*co*-FMA_{40})[10] with P(MMA_{74}-*co*-$MIMA_{26}$)[9] at room temperature and with a motif ratio of 1:1. Spectra of the heating cycle (red), spectrum of the maximum temperature (black) and spectra of the cooling cycle (blue). The entire temperature cycle from 30-190-30 °C in the complete wavenumber range.

DSC and FTIR spectra of the crosslinked PFMA5.

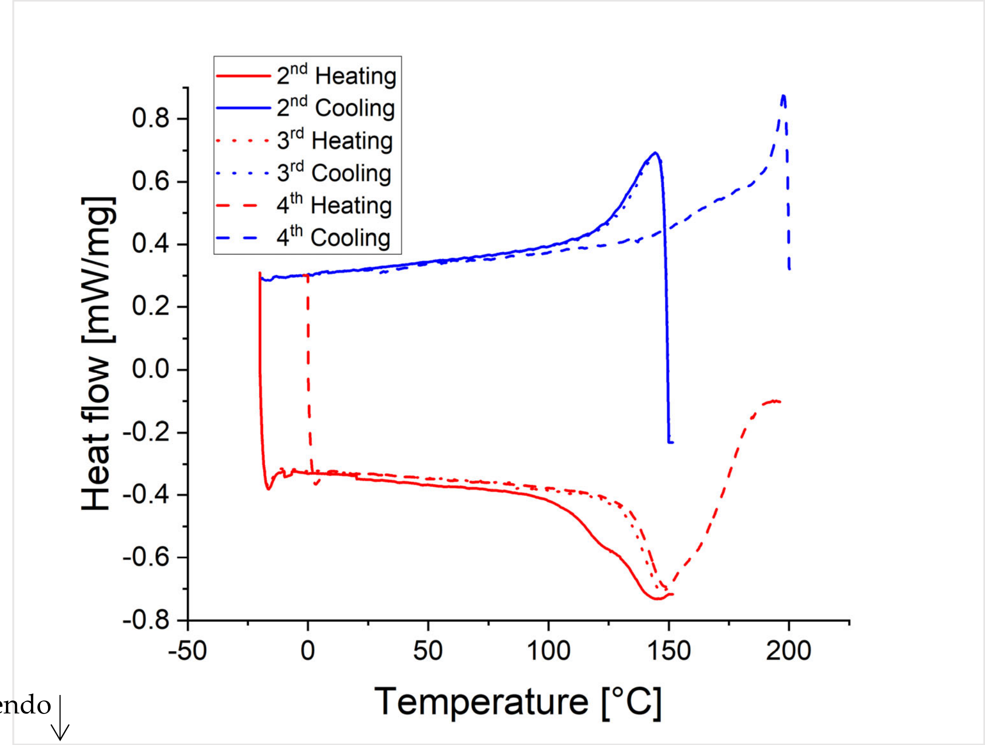

Figure S35. DSC heating (red) and cooling (blue) curves of the crosslinked PFMA[5] with BMI in a molar ratio of 1:1 at room temperature. The endothermic peak indicates the retro-Diels-Alder reaction.

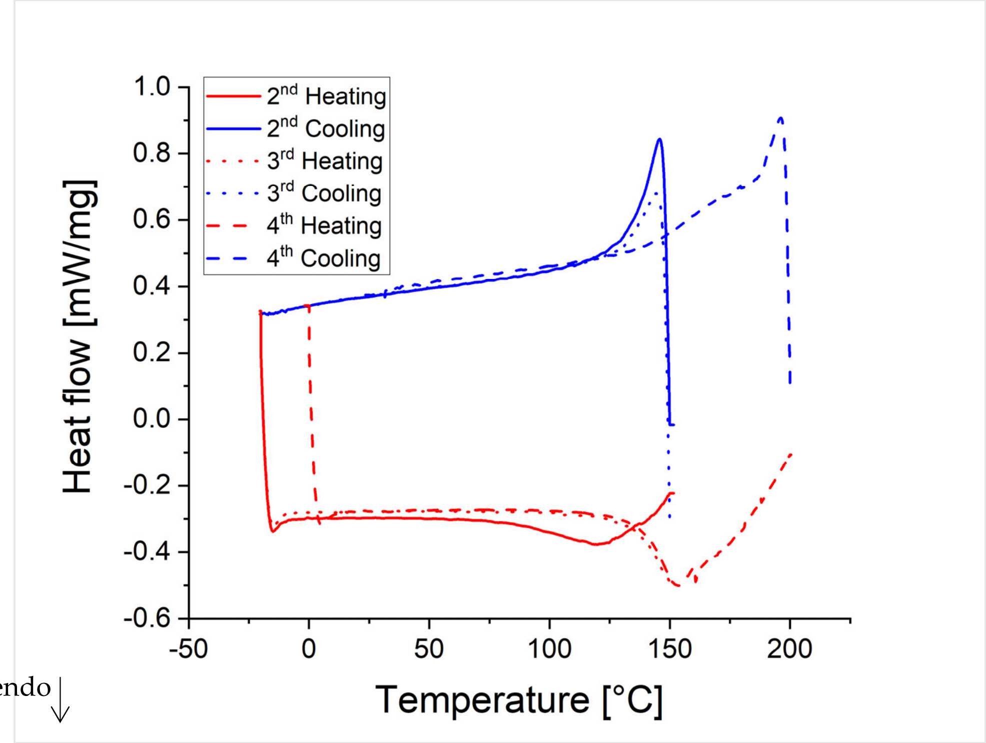

Figure S36. DSC heating (red) and cooling (blue) curves of the crosslinked PFMA[5] with BMI in a molar ratio of 1:1 at 120 °C. The endothermic peak indicates the retro-Diels-Alder reaction.

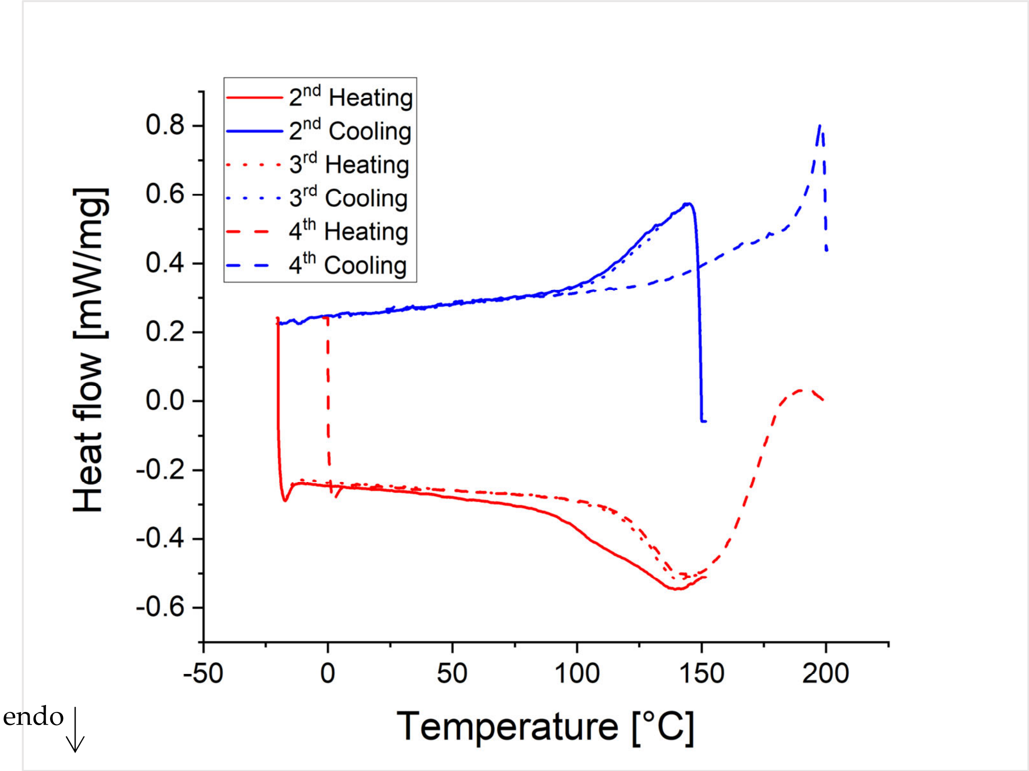

Figure S37. DSC heating (red) and cooling (blue) curves of the **c**rosslinked PFMA[5] with BMI in a molar ratio of 1:2 at room temperature. The endothermic peak indicates the retro-Diels-Alder reaction.

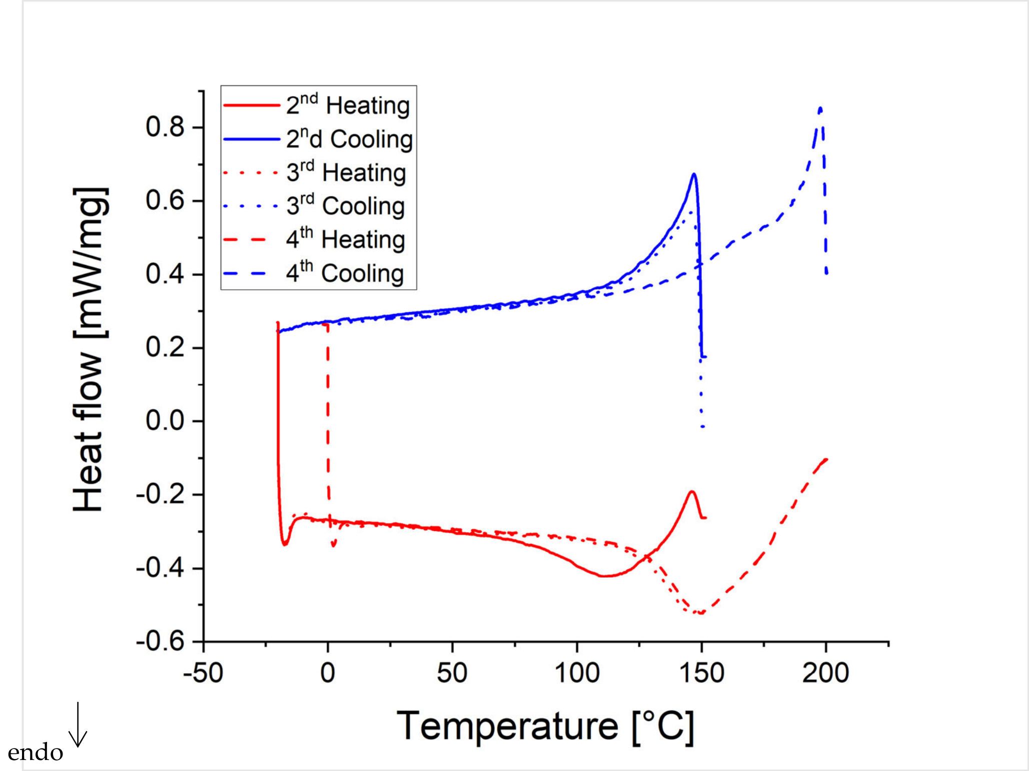

Figure S38. DSC heating (red) and cooling (blue) curves of the **c**rosslinked PFMA[5] with BMI in a molar ratio of 1:2 at 120 °C. The endothermic peak indicates the retro-Diels-Alder reaction.

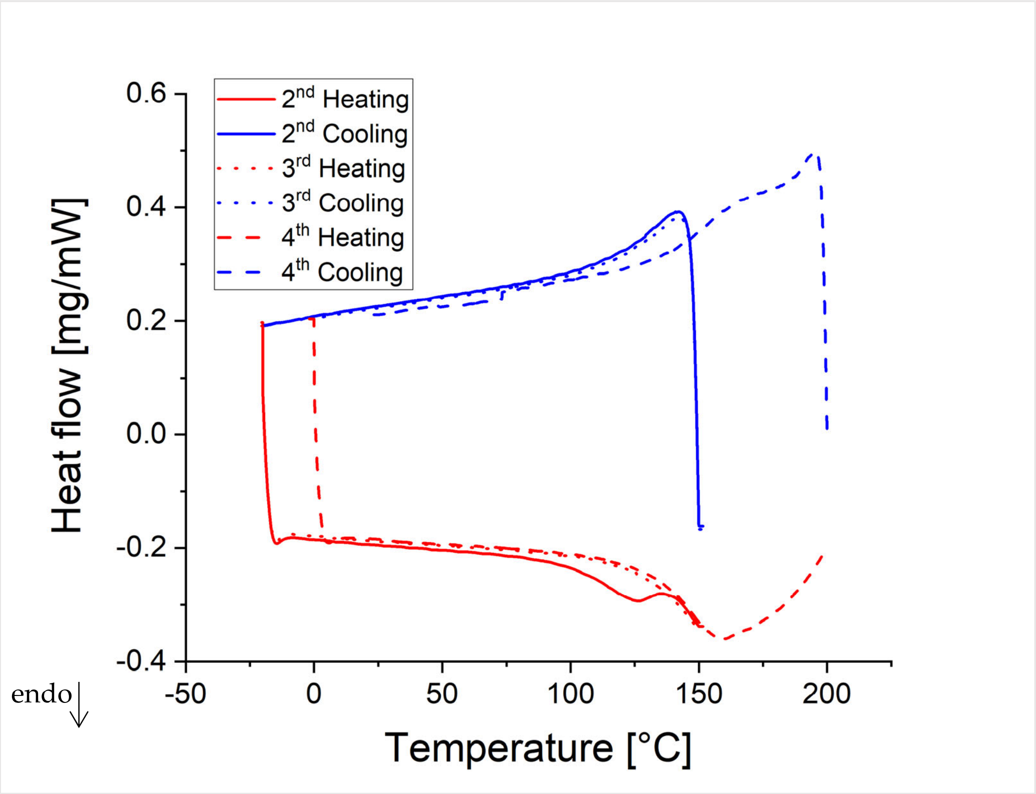

Figure S39. DSC heating (red) and cooling (blue) curves of the crosslinked PFMA[5] with P(MMA$_{74}$-*co*-MIMA$_{26}$)[9]. The endothermic peak indicates the retro-Diels-Alder reaction.

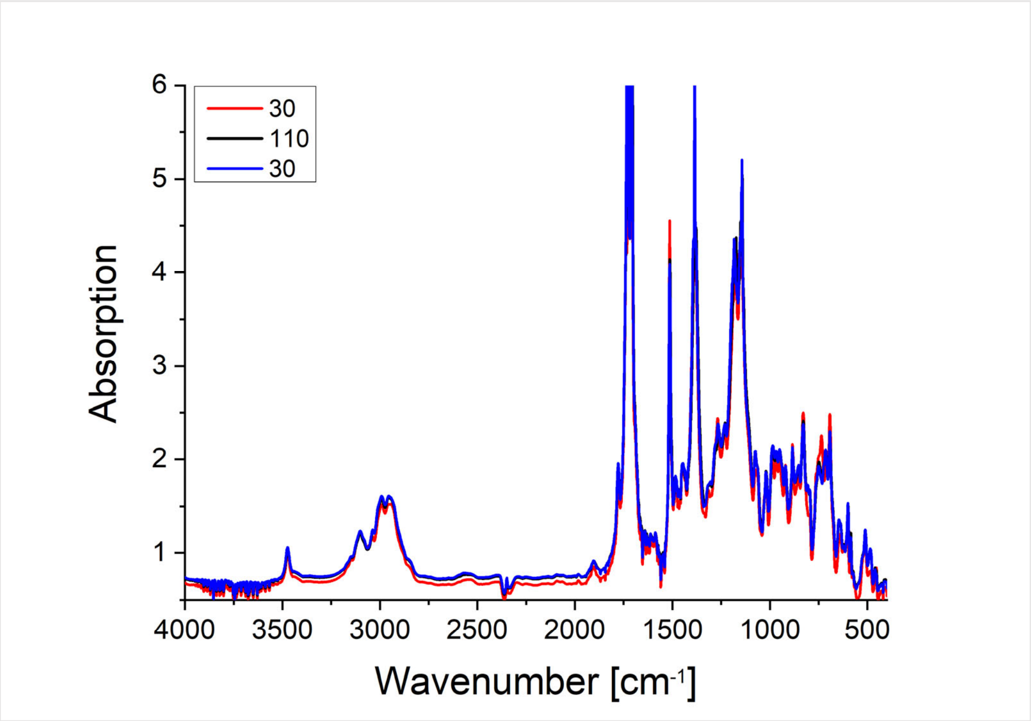

(a)

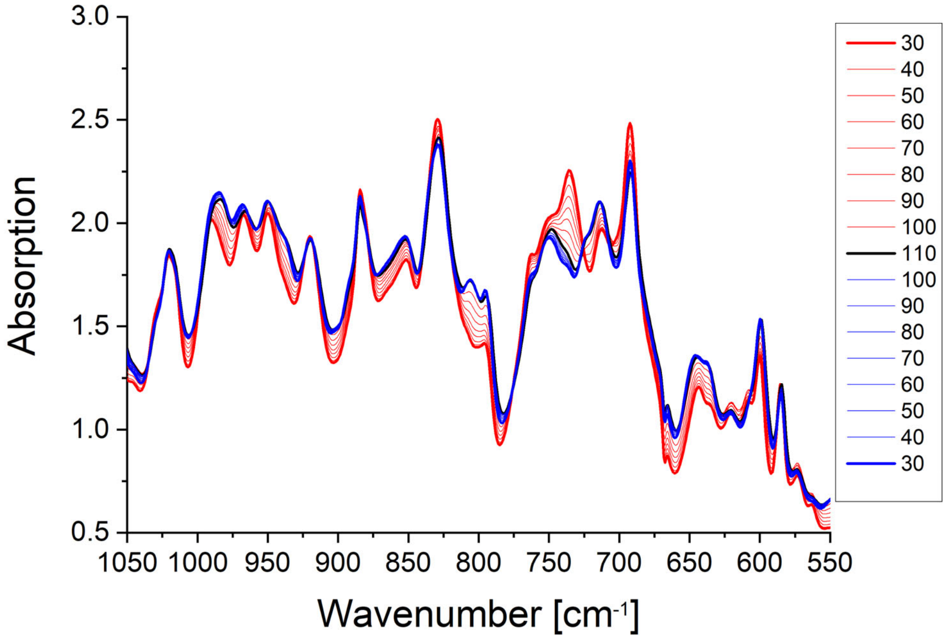

(b)

Figure S40. Temperature dependent FTIR spectra of the crosslinked PFMA[5] with BMI at room temperature and with a motif ratio of 1:1. Spectra of the heating cycle (red), spectrum of the maximum temperature (black) and spectra of the cooling cycle (blue). The entire temperature cycle from 30-110-30 °C in the complete wavenumber range (a) and from 1050 to 550 cm^{-1} (b) is shown, as the ring deformation mode of the maleimide ring formed in the retro-Diels-Alder reaction can be observed.

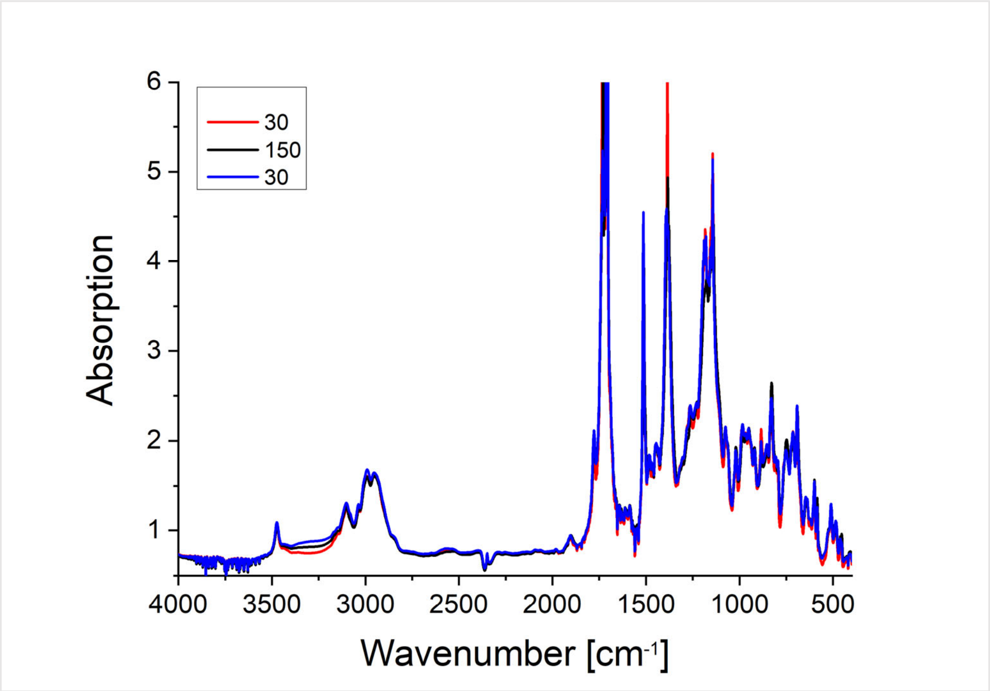

(a)

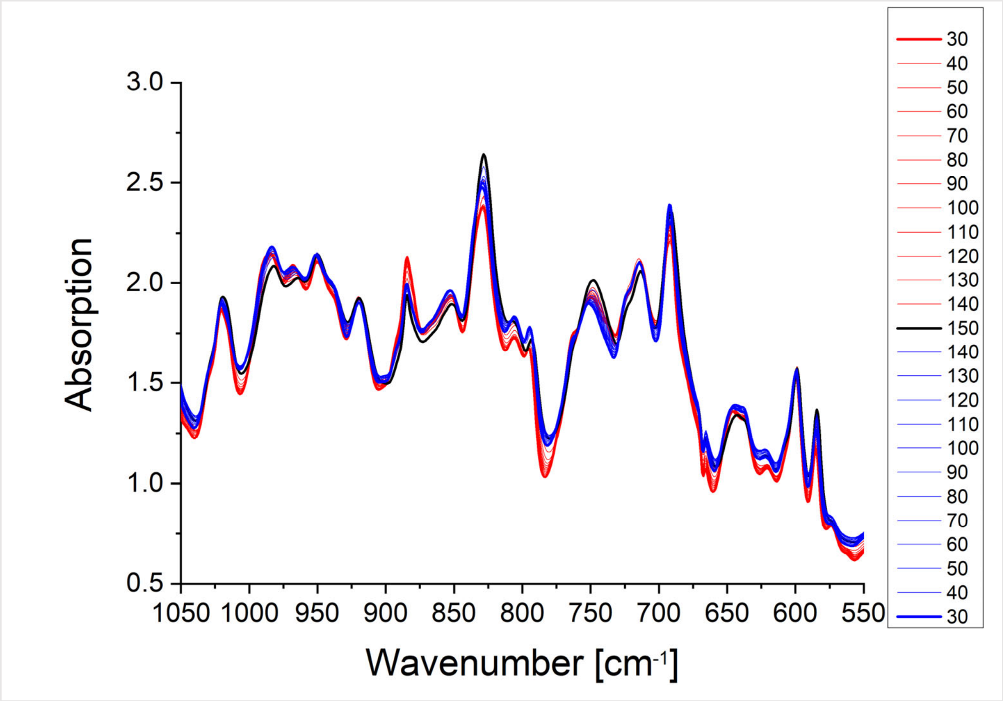

(b)

Figure S41. Temperature dependent FTIR spectra of the crosslinked PFMA[5] with BMI at room temperature and with a motif ratio of 1:1. Spectra of the heating cycle (red), spectrum of the maximum temperature (black) and spectra of the cooling cycle (blue). The entire temperature cycle from 30-150-30 °C in the complete wavenumber range (a) and from 1050 to 550 cm^{-1} (b) is shown, as the ring deformation mode of the maleimide ring formed in the retro-Diels-Alder reaction can be observed.

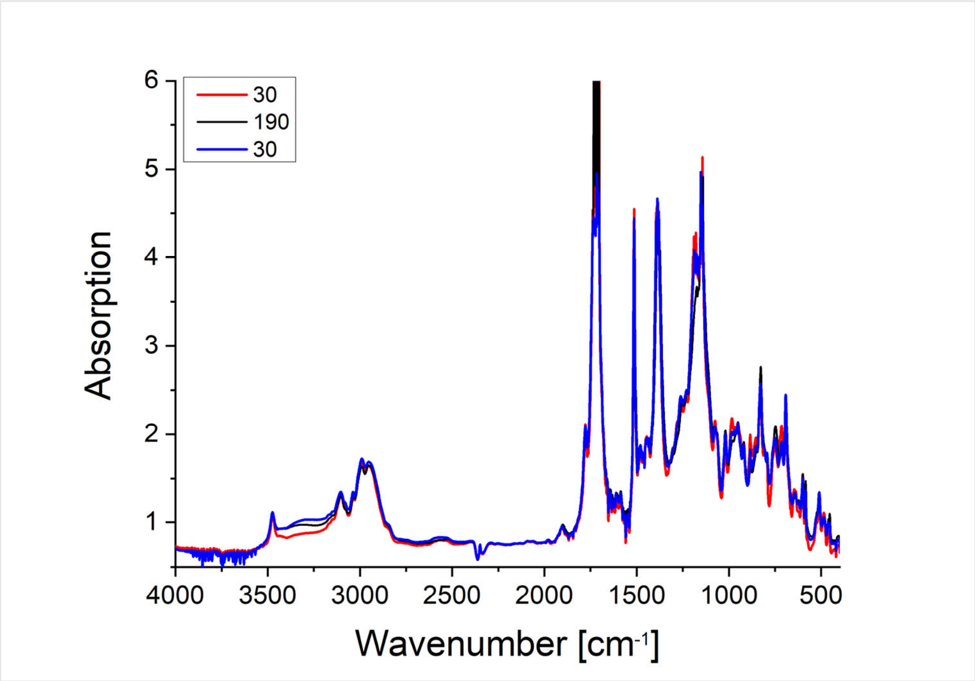

(a)

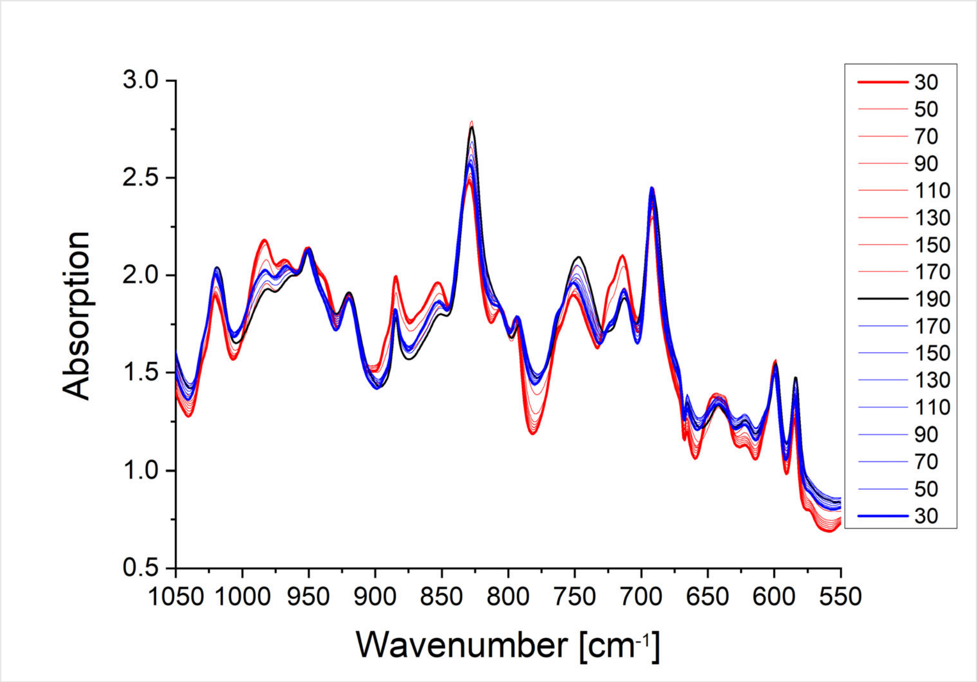

(b)

Figure S42. Temperature dependent FTIR spectra of the crosslinked PFMA[5] with BMI at room temperature and with a motif ratio of 1:1. Spectra of the heating cycle (red), spectrum of the maximum temperature (black) and spectra of the cooling cycle (blue). The entire temperature cycle from 30-190-30 °C in the complete wavenumber range (a) and from 1050 to 550 cm^{-1} (b) is shown, as the ring deformation mode of the maleimide ring formed in the retro-Diels-Alder reaction can be observed.

The crosslinked PFMA[5] with P(MMA_{74}-*co*-$MIMA_{26}$)[9] could not be measured by temperature dependent FTIR because no stable film could be formed on the KBr window.

DSC and FTIR spectra of the crosslinked P(MMA_{89}-*co*-FMA_{11})[24].

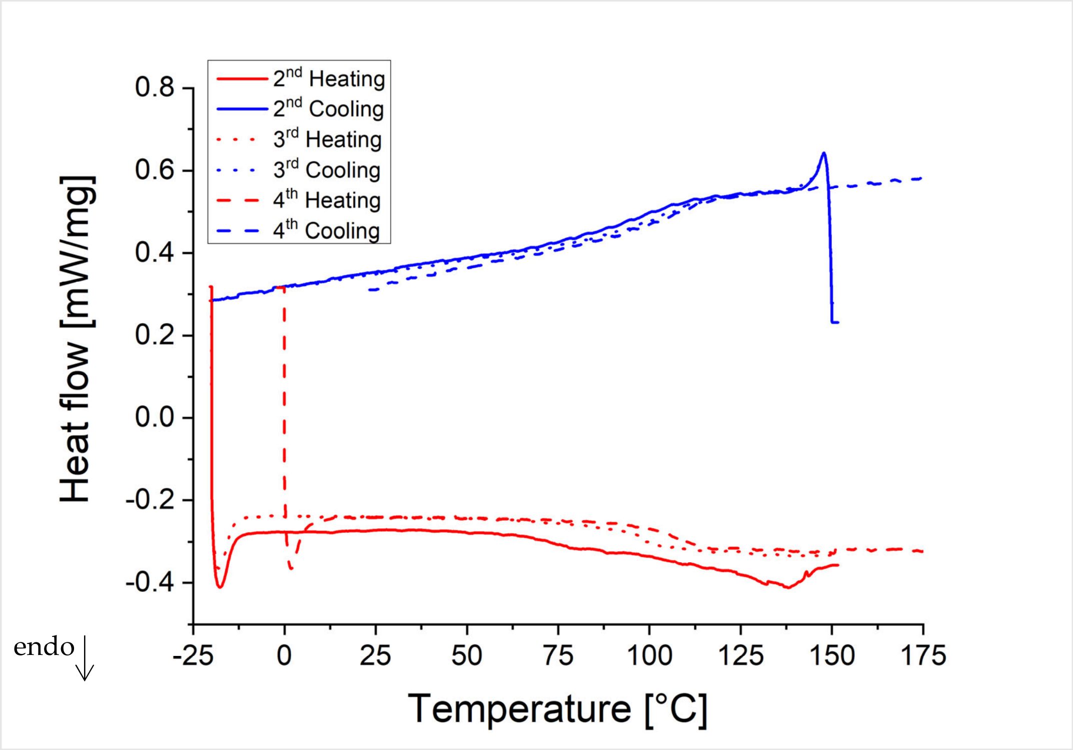

Figure S43. DSC heating (red) and cooling (blue) curves of the **c**rosslinked P(MMA_{89}-*co*-FMA_{11})[24] with BMI at room temperature with a molar ratio of 1:2. The endothermic peak indicates the retro-Diels-Alder reaction only in the second heating curve.

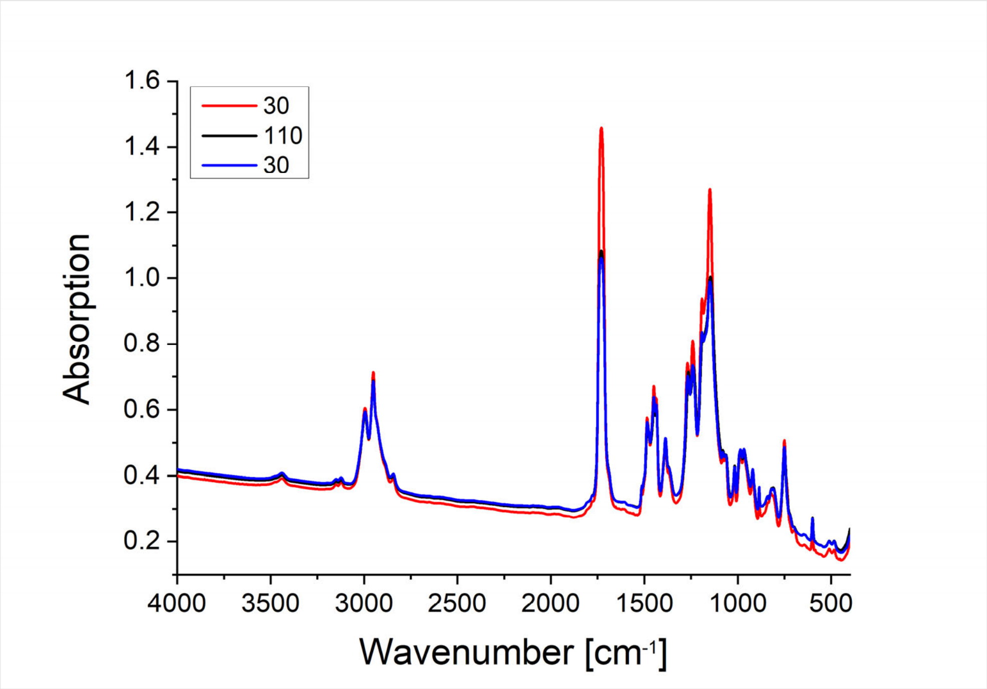

(a)

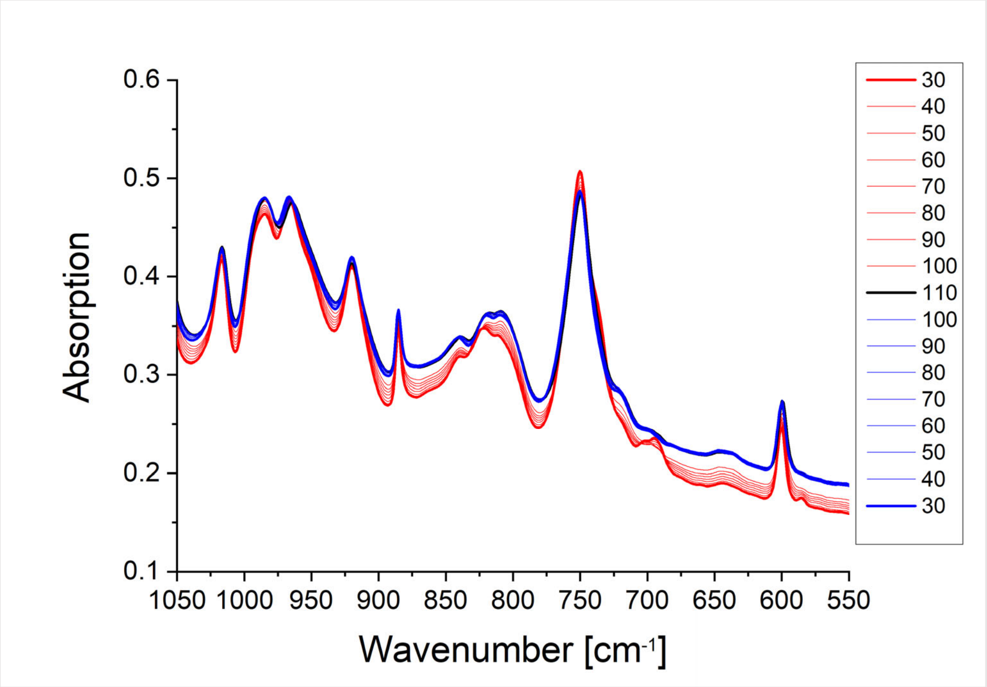

(b)

Figure S44. Temperature dependent FTIR spectra of the crosslinked P(MMA_{89}-*co*-FMA_{11})[24] with BMI at room temperature and with a motif ratio of 1:1. Spectra of the heating cycle (red), spectrum of the maximum temperature (black) and spectra of the cooling cycle (blue). The entire temperature cycle from 30-110-30 °C in the complete wavenumber range (a) and from 1050 to 550 cm^{-1} (b) is shown, as the ring deformation mode of the maleimide ring formed in the retro-Diels-Alder reaction cannot be observed.

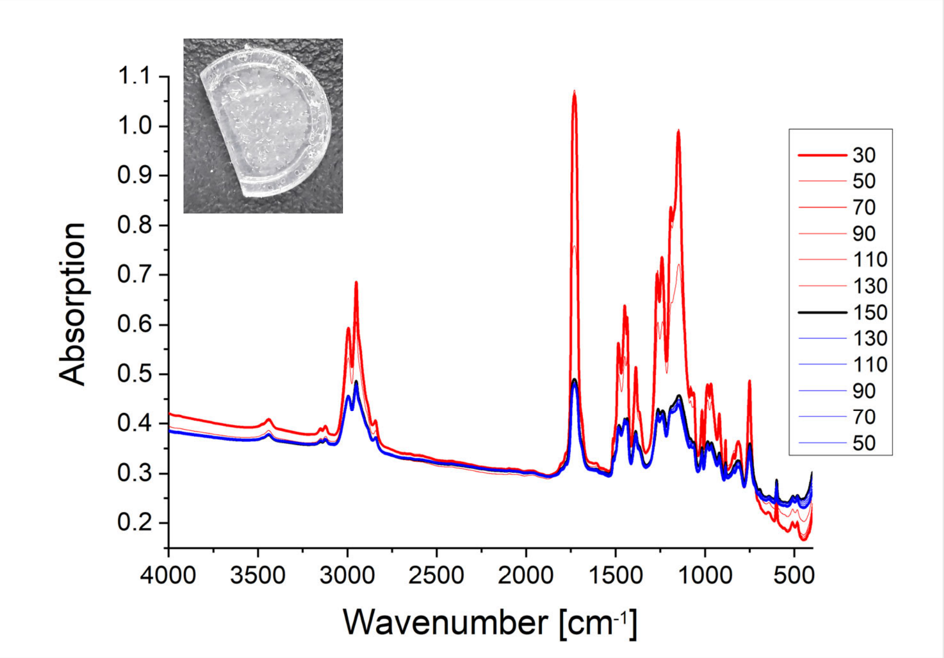

(a)

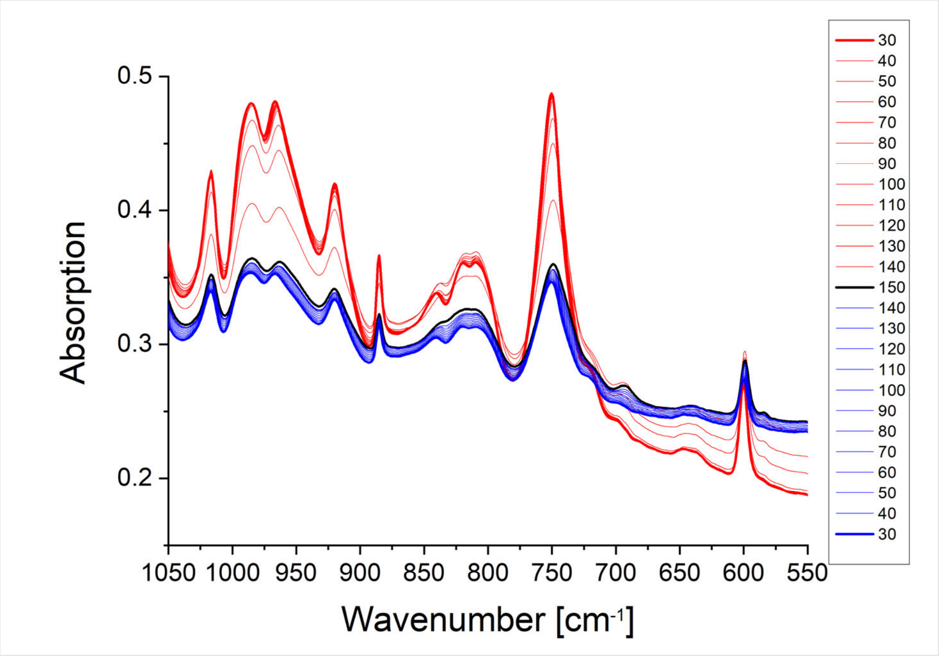

(b)

Figure S45. Temperature dependent FTIR spectra of the crosslinked P(MMA$_{89}$-*co*-FMA$_{11}$)$_{24}$ with BMI at room temperature and with a motif ratio of 1:1. Spectra of the heating cycle (red), spectrum of the maximum temperature (black) and spectra of the cooling cycle (blue). The entire temperature cycle from 30-10-30 °C in the complete wavenumber range (a) and from 1050 to 550 cm^{-1} (b) is shown, as the ring deformation mode of the maleimide ring formed in the retro-Diels-Alder reaction cannot be observed. The decrease of the absorption in the heating cycle indicates a change of the polymer film on the KBr window, as can be seen in the upper picture, de-wetting occurs.

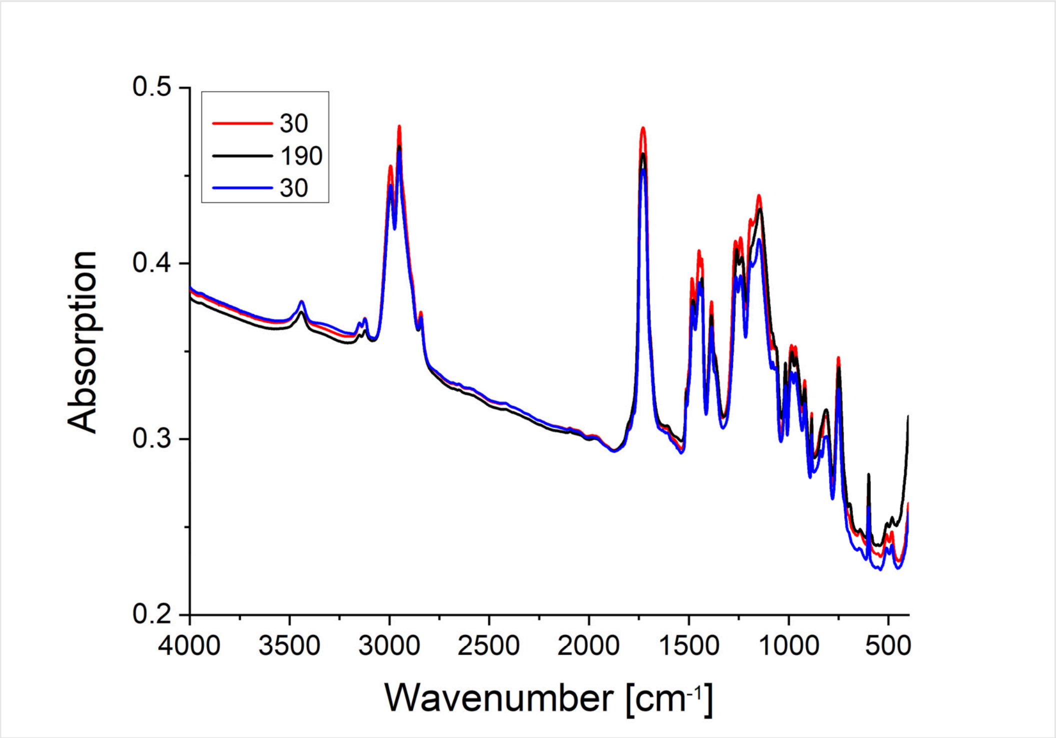

Figure S46. Temperature dependent FTIR spectra of the crosslinked P(MMA_{89}-*co*-FMA_{11})[24] with BMI at room temperature and with a motif ratio of 1:1. Spectra of the heating cycle (red), spectrum of the maximum temperature (black) and spectra of the cooling cycle (blue). The entire temperature cycle from 30-10-30 °C in the complete wavenumber range (a) and from 1050 to 550 cm^{-1} (b) is shown, as the ring deformation mode of the maleimide ring formed in the retro-Diels-Alder reaction cannot be observed well.

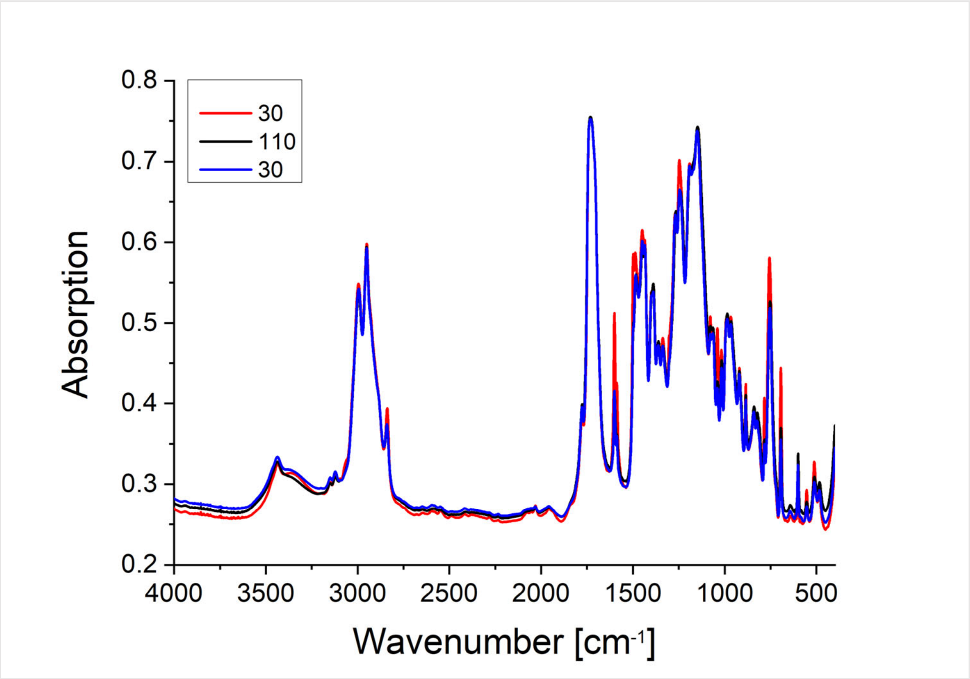

(a)

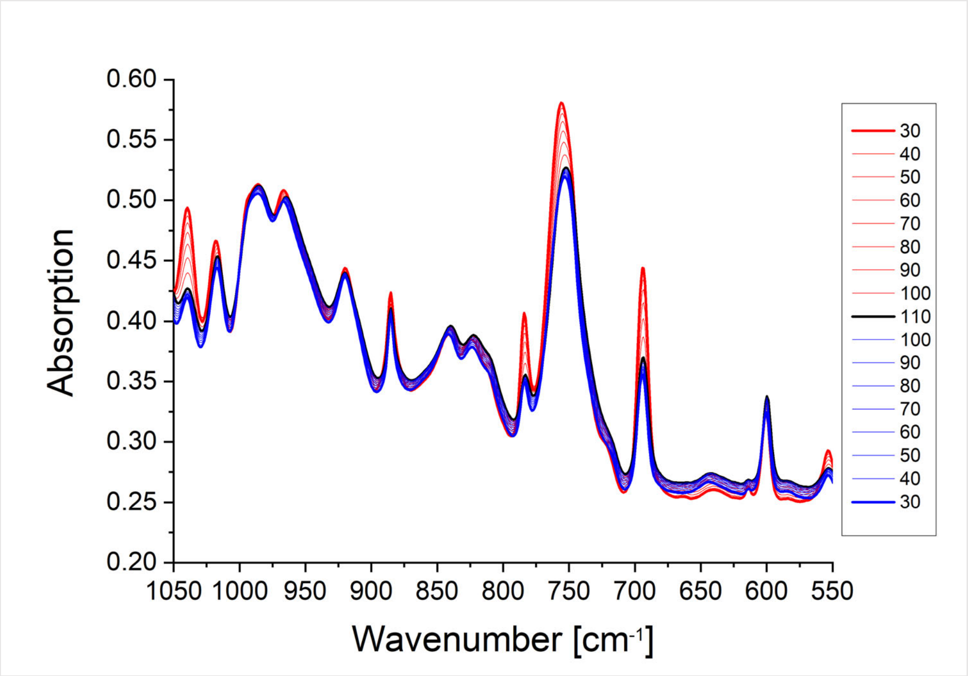

(b)

Figure S47. Temperature dependent FTIR spectra of the crosslinked P(MMA$_{89}$-*co*-FMA$_{11}$)[24]with P(MMA$_{74}$-*co*-MIMA$_{26}$)[9] at room temperature and with a motif ratio of 1:1. Spectra of the heating cycle (red), spectrum of the maximum temperature (black) and spectra of the cooling cycle (blue). The entire temperature cycle from 30-110-30 °C in the complete wavenumber range (a) and from 1050 to 550 cm^{-1} (b) is shown, as the ring deformation mode of the maleimide ring formed in the retro-Diels-Alder reaction can be observed.

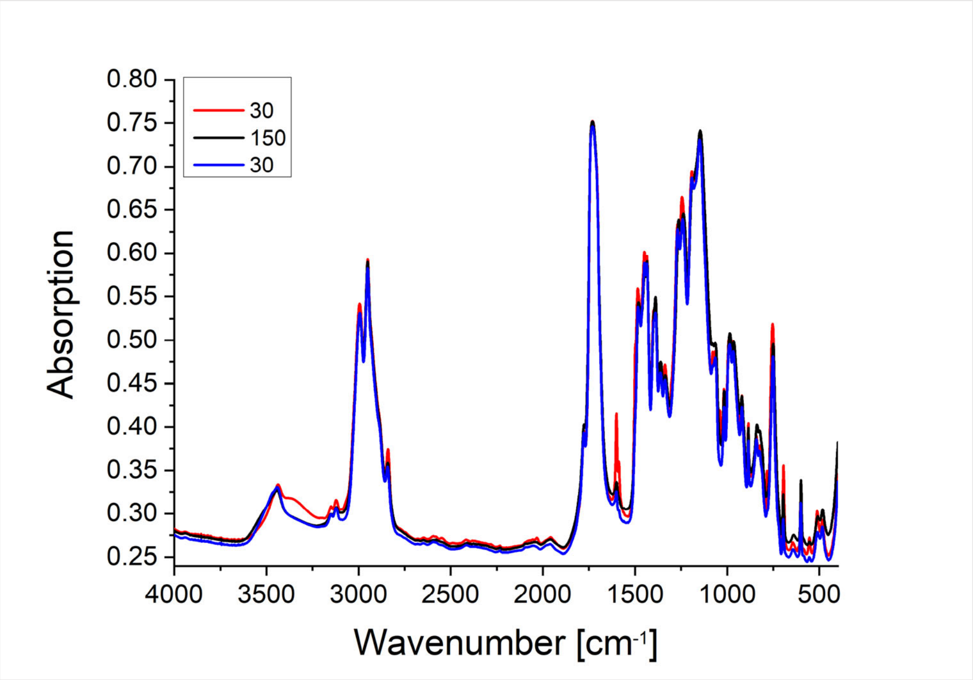

(a)

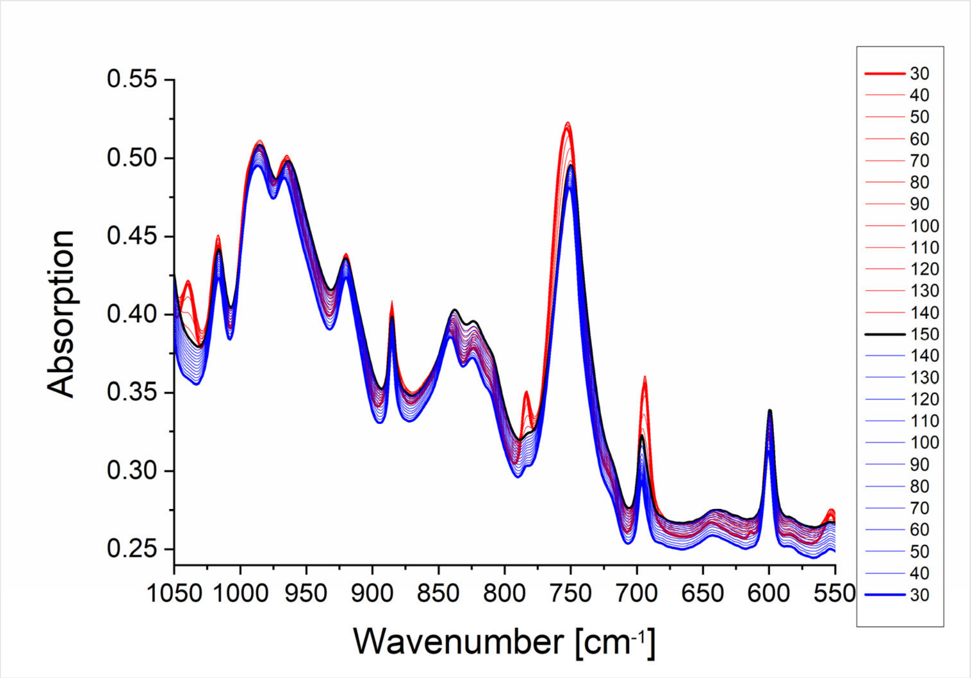

(b)

Figure S48. Temperature dependent FTIR spectra of the crosslinked P(MMA$_{89}$-*co*-FMA$_{11}$)[24]with P(MMA$_{74}$-*co*-MIMA$_{26}$)[9] at room temperature and with a motif ratio of 1:1. Spectra of the heating cycle (red), spectrum of the maximum temperature (black) and spectra of the cooling cycle (blue). The entire temperature cycle from 30-150-30 °C in the complete wavenumber range (a) and from 1050 to 550 cm^{-1} (b) is shown, as the ring deformation mode of the maleimide ring formed in the retro-Diels-Alder reaction can be observed.

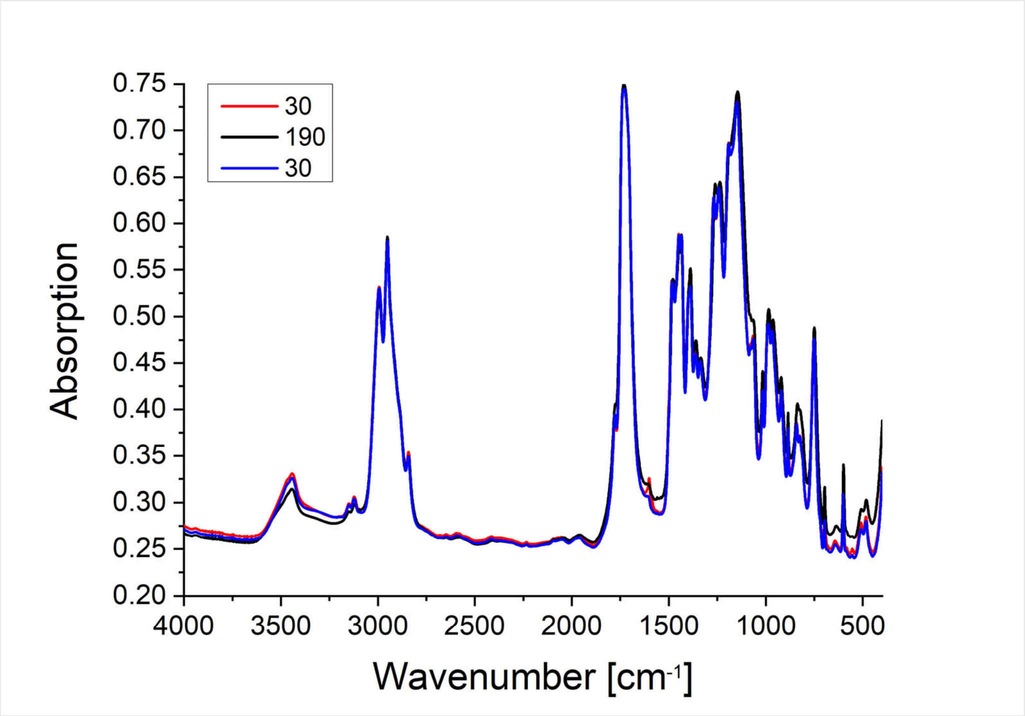

Figure S49. Temperature dependent FTIR spectra of the crosslinked P(MMA_{89}-*co*-FMA_{11})[24] with P(MMA_{74}-*co*-$MIMA_{26}$)[9] at room temperature and with a motif ratio of 1:1. Spectra of the heating cycle (red), spectrum of the maximum temperature (black) and spectra of the cooling cycle (blue). The entire temperature cycle from 30-150-30 °C in the complete wavenumber range.

Swelling test with the plasticizer anisole

In order to even observe thermoreversibility in the cross-linked P(MMA$_{89}$-*co*-FMA$_{11}$)[24] with a high glass transition temperature, a plasticizer in form of a solvent was added and the swollen samples were measured in the DSC. In this case, anisole was used as plasticizer because it has a boiling point of 154 °C and it is a solvent for the initial polymer. The boiling temperature was crucial in this case, as the swollen polymer in the DSC is heated to 130 °C to exceed the retro-Diels-Alder reaction temperature of 120 °C. The next Fig. S50 shows the DSC measurements of the crosslinked P(MMA$_{89}$-*co*-FMA$_{11}$)[24] without plasticizer and only the plasticizer anisole.

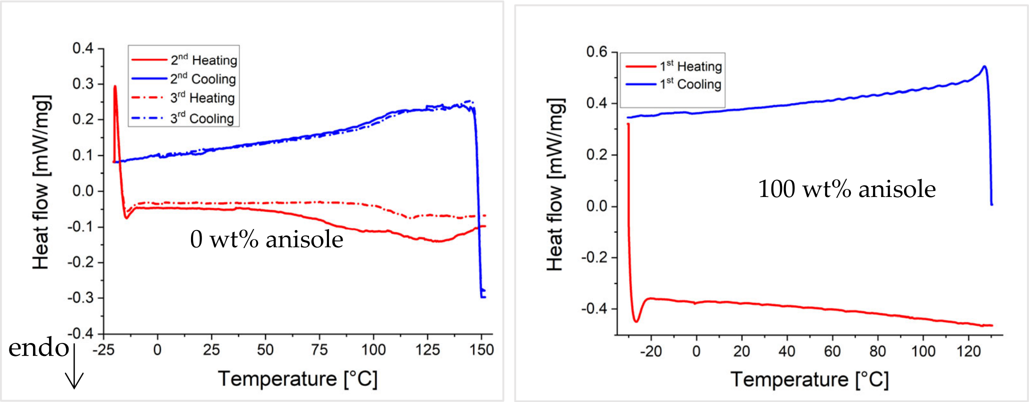

Figure S50. DSC heating (red) and cooling (blue) curves of the **c**rosslinked P(MMA$_{89}$-*co*-FMA$_{11}$)[24] with BMI and anisole.

The next Figure S51 shows the DSC measurement series of the swollen P(MMA$_{89}$-*co*-FMA$_{11}$)[24]. For this purpose, the crosslinked P(MMA$_{89}$-*co*-FMA$_{11}$)[24] was weighed in together with the following weight percentages of anisole a) 5 wt%; b) 10 wt%; c) 25 wt%; d) 50 wt%, the crucible was closed and allowed to swell for one hour at room temperature.

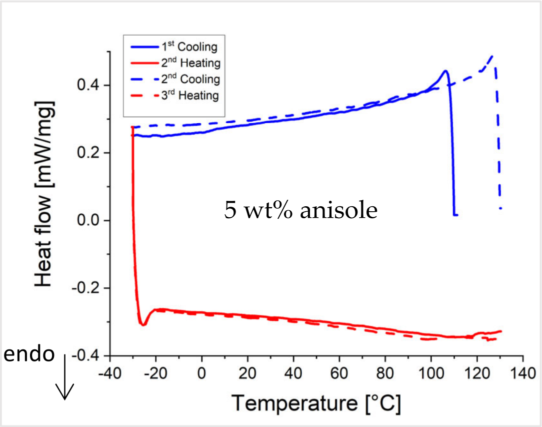

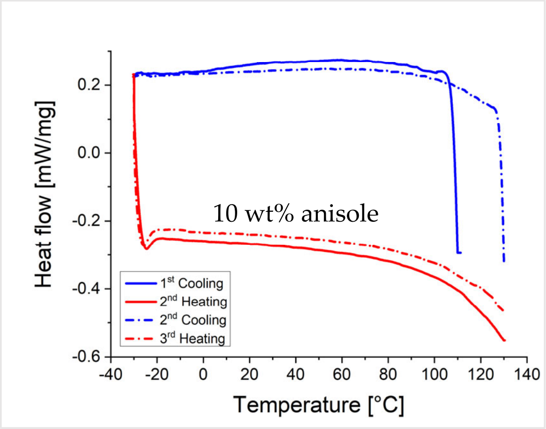

(a)

(b)

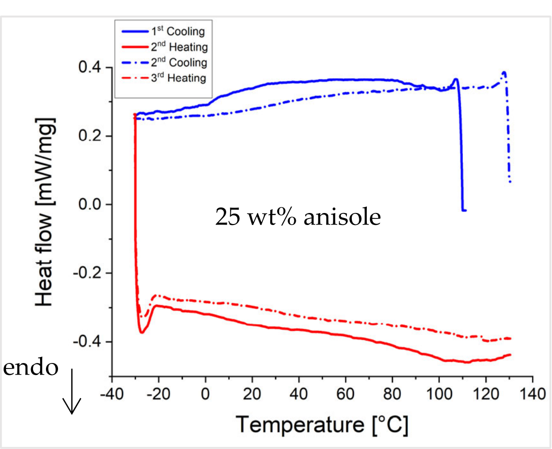

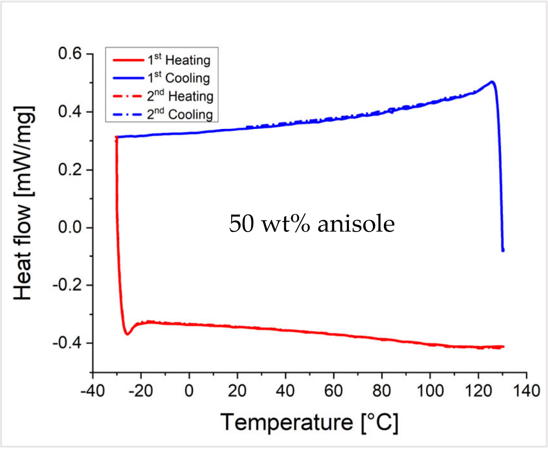

(c)

(d)

Figure S51. DSC heating (red) and cooling (blue) curves of the **c**ross-linked P(MMA$_{89}$-*co*-FMA$_{11}$)[24] with BMI and different weight percentages of anisole (a) 5 wt%; b) 10 wt%; c) 25 wt%; d) 50 wt%).
